# Correction: The effectiveness of mindfulness alone compared to exercise and mindfulness on fatigue in women with gynaecology cancer (GEMS): Protocol for a randomised feasibility trial

**DOI:** 10.1371/journal.pone.0340148

**Published:** 2026-01-02

**Authors:** The PLOS One Staff

## Notice of Republication

An incomplete, earlier version of this article was published in error. The publisher apologises for the error. This article was republished on December 17, 2025, to correct for this error. Please download the article again to view the correct version. The originally published, uncorrected article and the republished, corrected article are provided here for reference.

## Supporting information

S1 File Originally published, uncorrected article. (PDF)

S2 File Republished, corrected article. (PDF)

## References

[1] McCloy K, Hughes C, Dunwoody L, Marley J, Cleland I, Cruciani F, et al. The effectiveness of mindfulness alone compared to exercise and mindfulness on fatigue in women with gynaecology cancer (GEMS): Protocol for a randomised feasibility trial. PLoS One. 2023;18(10):e0278252. doi: 10.1371/journal.pone.0278252 37883461 PMC10602305

