# Supplementary material for: The effectiveness of mindfulness alone compared to exercise and mindfulness on fatigue in women with gynaecology cancer (GEMS): Protocol for a randomised feasibility trial
Source: PLoS One. 2023 Oct 26;18(10):e0278252. doi: 10.1371/journal.pone.0278252 (PMC10602305; doi:10.1371/journal.pone.0278252)
Supplement: S1 File — Study protocol. (DOCX) [file pone.0278252.s002.docx]

# Study Protocol

# Title: Randomised controlled feasibility trial evaluating the effectiveness of mindfulness compared to exercise and mindfulness on fatigue in women with gynaecology cancer.

Gynaecology Exercise and Mindfulness Study (GEMS)

Intervention protocol

Section 1 Background and rationale.

Introduction

Gynaecology cancer encompasses ovarian, endometrial, cervical, vaginal, vulva, fallopian tube, and placenta (Lin *et al.* 2016). In the United Kingdom (UK) 21,493 new cases of gynaecological cancer are diagnosed every year (*Gynaecological Cancer Research Charity.* 2020). Treatment modalities can be effective in managing and treating these cancers but may lead to unwanted long term side effects. One side effect is fatigue, the prevalence of which, for all types of cancer, was identified as 52% and for the gynaecology cancer population, this varied between 17-33% with many reporting to experience cancer related fatigue (CRF) many years after treatment (Sekse *et al.* 2015; Ma *et al.* 2020; Sheehan *et al.* 2020a). It has been described not only as a physical sensation but also has emotional and cognitive symptoms suggesting that it is multi-dimensional (McMillan and Newhouse 2011; Coleman *et al.* 2012; de Raaf *et al.* 2013; Dennett *et al.* 2016). To date, few studies evaluate interventions that target all dimensions of fatigue suggesting a multi-modal approach that addresses all aspects is required.

Mechanisms.

Risk factors for CRF that have been described, include depression and insomnia suggesting that the presence of these are not only possibly correlated but may also have an impact on the levels and severity of CRF (Bower 2014). Indeed, the term ‘symptom cluster’ has recently been adopted to describe this and the most common symptoms in this cluster have been identified as depression, insomnia, and fatigue (Payne *et al.* 2008; Oh and Seo 2011; Joly *et al.* 2019). Which suggests that managing fatigue would require the incorporation of interventions that deal with the multiple symptoms of depression, insomnia, and fatigue collectively (Bower 2014; Naraphong *et al.* 2015; Poort *et al.* 2020).

Existing Knowledge

There has been much research conducted that has shown a positive effect of exercise with regards to CRF (Wang *et al.* 2011; Keogh and MacLeod 2012; Cramp and Byron‐Daniel 2012; Zou *et al.* 2014; Tian *et al.* 2016; Juvet *et al.* 2017; Schuler *et al.* 2017; Mugele *et al.* 2019). A number of randomised controlled trials have shown that exercise can result in improvements in CRF (Mock *et al.* 2005; Donnelly *et al.* 2011; Wenzel *et al.* 2013; Zhang, Qi *et al.* 2018). However, the effect of exercise on CRF and quality of life (QOL) remains ambiguous (Velthuis *et al.* 2010; Lin *et al.* 2016). Some cancer studies have reported that the impact of interventions, such as exercise, have shown a positive outcome on CRF and QOL and others have demonstrated no change in either CRF or QoL (von Gruenigen *et al.* 2009; Donnelly *et al.* 2011; Moonsammy *et al.* 2013; Smits *et al.* 2014; Basen-Engquist *et al.* 2014; Mizrahi *et al.* 2015; Al Maqbali 2019). This concept that exercise may not improve all dimensions of QOL or CRF suggests that exercise alone may not be enough to ameliorate fatigue and improve QOL and a multidimensional approach may be required.

Despite the evidence of the positive effect of exercise on CRF, adherence to exercise remains a problem with less than 20% of women with ovarian cancer exercising (Joly *et al.* 2019). Barriers that have been identified for non-participation include time, cost, weather, side effects of treatment or exercise, social aspects, not being aware of guidelines, fatigue, and psychological barriers such as motivation (Hardcastle *et al.* 2018; Lavallee *et al.* 2018). In contrast, the facilitators to exercise that are identified were improved physical and mental wellbeing, decreased feelings of stress, enjoyment, satisfaction, and control over health (Blaney *et al.* 2013; Gho *et al.* 2014; Clifford *et al.* 2018). Understanding what barriers and facilitators are perceived by patients may be required to engage patients with exercise and change lifestyle behaviours. The current behavioural change methods follow models such as the COM-B (Capability, Opportunity, Motivation, and Behaviour). This model denotes that for a behaviour to change 3 aspects are needed; capability, opportunity, and motivation are required. The COM-B is a hub that sits within the Behaviour Change Wheel (BCW) which was synthesised from previous frameworks and enhances the development of behavioural interventions (Cowdell and Dyson 2019; Howlett *et al.* 2019). Interventions that may have begun to identify an approach that addresses some of these psychological barriers such as motivation and that may open a path to consider changing lifestyles, are mindfulness-based (Lucas, A. R. ( 1 ) *et al.* 2017; Cohen *et al.* 2019).

Mindfulness is the practice of intentional awareness in the present moment without judgement. Its practice encourages participants to develop an awareness of current emotions and thoughts with compassion and kindness and will lead to better control of cognition, emotion, and behaviour (Park *et al.* 2020). Research in cancer demonstrates the positive effects of mindfulness on both CRF and QOL and recommend it as an intervention (Leydon *et al.* 2012; Van Der Lee, and Garssen 2012; Lengacher *et al.* 2012; Bruggeman-Everts *et al.* 2015; Johns, S. A. *et al.* 2015; Huang, H. -. *et al.* 2016; Johns, S. A. *et al.* 2016; Lengacher *et al.* 2017; Reich *et al.* 2017a; Zhang, Qiuxiang *et al.* 2019; Xie *et al.* 2020; Xunlin *et al.* 2020; Park *et al.* 2020). Though some of the effect sizes shown in studies were small and did not reach minimal clinical significance, there was still however an effect on psychological distress, which is defined as an emotion that is not pleasant (Cramer *et al.* 2012; Haller *et al.* 2017; Cillessen *et al.* 2019). This effect on the psychological distress and particularly on the symptoms of anxiety, depression, sleep, stress, and quality of life have been shown to significantly improve following the introduction of mindfulness (Carlson and Garland 2005; Huang, J. and Shi 2016; Reich *et al.* 2017b). Some of these symptoms are also present within the ‘cluster symptoms’ previously identified suggesting that there may also be an improvement in fatigue alongside these symptoms.

Need for a trial

Most of the current studies that involve exercise or mindfulness for the management of CRF and psychological distress tend to be in the breast cancer population making it difficult to generalise findings to other cancer populations (Brandenbarg *et al.* 2018; Stelten *et al.* 2020). It would appear that both exercise and mindfulness have individually as interventions demonstrated positive effects on CRF, anxiety, depression, and sleep. Whether mindfulness and indeed adding mindfulness to an exercise regime for managing fatigue in women with gynaecology cancer is of benefit, is yet to be explored. Therefore, this study will aim to assess how feasible and acceptable the interventions, mindfulness, and exercise, will be and if there is any effect on fatigue for the gynaecology cancer patient.

Primary Aim

To explore the feasibility of conducting an 8- week online mindfulness and home-based walking and strength training programme for the management of cancer related fatigue in women with gynaecology cancer. The following objectives will assess feasibility outcomes:

1. To assess participant intervention adherence, study retention and attrition rate.
2. Explore the acceptability of using an online platform for the delivery and data collection of the exercise and mindfulness programme.
3. Explore the perspectives of participants concerning satisfaction (eg the intervention and its’ delivery) perceived value, barriers and facilitators of the online programme.

Secondary aim

To evaluate the effect of an 8 week exercise and mindfulness programme on cancer related fatigue as a primary clinical outcome and the effect on anxiety, depression, sleep and QoL as a secondary clinical outcome in an online setting.

Section 2 Methods

Study design

The study design and development have been reviewed and feedback considered from 2 patient and public involvement representatives.

This design will involve 2 parts firstly, an intervention part which is designed as an open, randomised, control trial with 1:1 allocation to either the mindfulness only group or the exercise and mindfulness group.

Following the intervention the second part will be online focus groups which will be conducted with participants once they have completed the intervention.

Study setting

Both parts of this study, intervention and focus groups, will be conducted online via a web-based, mobile application interface. Participants will be notified that data usage with this app will be minimal and battery usage of mobile devices may increase requiring additional charging of device. Therefore, participants should not incur any additional financial burden.

Participants & Eligibility

Eligibility criteria:

Inclusion criteria:

1. Women >18 years old and able to speak and read English.
2. Diagnosed with stage I-IV gynaecology cancer including endometrial, ovarian, cervical, vaginal, vulva, fallopian tube.
3. A minimum of 3 months post-treatment and up to 5 years.
4. Experiencing fatigue and at screening have a numerical rating score of $\geq$4 on a scale of 0-10.
5. Currently sedentary (exercising < once a week for 20 minutes at a vigorous intensity or, two times per week for 30 minutes at moderate intensity or < 20 minutes three times per week, for the past 6 months).
6. Ambulatory and without the use of a walking aid.
7. Capacity to give informed consent.
8. Access to computer/tablet/smartphone.
9. Access to internet.

Exclusion criteria:

1. Currently actively and regularly practising mindfulness.
2. Have a confirmed diagnoses of Schizophrenia spectrum disorder, Bipolar disorder, Post-traumatic stress disorder, or risk factors for psychosis (eg personality disorder) as participation in a mindfulness program is contraindicated and maybe detrimental to well-being (Van Dam *et al.* 2018).
3. Have an existing medical condition that may inhibit safe participation in the exercise part of the intervention study.
4. Previously diagnosed with a fatigue-related co-morbid medical condition (i.e. Fibromyalgia, Chronic Fatigue Syndrome, Multiple Sclerosis (MS), Myalgic Encephalopathy (ME), Lupus or Arthritis)
5. Have no e-mail address.
6. Participating in other intervention trials.

Sample size

The sample size for this study is based upon feasibility and review of similar studies. The literature demonstrates that feasibility studies with similar interventions and methodologies within the cancer population recruit on average 40 participants in total (Eyles *et al.* 2015a; Victorson *et al.* 2017; Price-Blackshear *et al.* 2020). Therefore it is anticipated that this study will recruit a total of 40 participants with 20 in each arm of the study.

Procedure & Materials

Recruitment.

This study will adopt the approach of rolling recruitment and once participants have been screened and consented will commence the intervention they have been randomised to. Recruitment will take place through the following charities: Cancer Focus, Macmillan and Action Cancer. These organisations have agreed to contact their service users through social media (Facebook, Twitter, Instagram), leaflet drops and other service providers the organisation provide such as counsellors, clinical nurse specialist, support workers etc (appendix 1). They have also provided written confirmation of their commitment to assist in recruitment for this study (appendix 2).

If participants are interested in the study they can self-refer and a contact number for the researcher will be made available through the leaflets, social media or service providers. Participants can call the researcher for further information and if willing and interested screening for eligibility will be performed by the researcher (appendix 3). Once this is complete and it is established that participants meet the criteria participants will be e-mailed participant information sheet by the researcher (appendix 4) and time given to read and ask any questions regarding this. Participants can contact the research team if they have any further questions. Consent (Appendix 5) will be obtained after participants have been given one week to consider the study. An Qualtrics link to an electronic version of the consent will be emailed to participants.

Randomisation

Participants will be randomly allocated to either group through 1:1 allocation. Participants will be randomly assigned using computer-generated random number tables. A researcher/statistician not involved in treatment delivery or collection of outcomes will generate the random allocation sequence. Concealment of treatment allocation will be by placing individual random assignments into serially numbered sealed envelopes and be held by researcher/statistician not involved in treatment or outcome measure collection.

Participants will be notified of which group they have been allocated to via the e-mail they have supplied. A member of the research team will e-mail the participant with this information.

Data collection methods

Outcome measures

Data collection for outcome measures will be self-report measures using valid and reliable tools for measuring these outcomes. Participants will be asked to complete these assessments following randomisation at baseline and on completion of the programme. At baseline participants will also complete demographic information such as age, cancer type, education, income, and education (appendix 6). All of this will be done via Qualtrics (appendix 7), an e-mail reminder will be sent 2 weeks after the initial invitation if completion has not occurred. Access to intervention will only happen once all baseline assessments have been completed.

The following self-report measures will be used to assess outcomes at baseline and post-intervention (copies and links to Qualtrics of all can be found in appendix 7).

Fatigue- The Functional Assessment of Chronic Therapy- Fatigue (FACIT-F) subscale will be used it is a 13- item questionnaire that uses a 5 point Likert scale and has been validated for use with various types of cancer and treatments (Al Maqbali *et al.* 2019; Carlson *et al.* 2019). The lower the score on this scale indicates the greater the fatigue with scores less than 20 representing severe fatigue and scores greater than 45 as normal (Sheehan *et al.* 2020b)

Quality of life- The Functional Assessment of Cancer Therapy General (FACT-G) is a 28- item rated scale. It assesses physical, social, emotional and functional wellbeing. (Cella *et al.* 1993). Higher scores in this questionnaire indicate better quality of life (Park *et al.* 2020)

The psychological outcomes will be assessed using the Hospital Anxiety and Depression Scale (HADS). (Zigmond and Snaith 1983). This questionnaire has 14 questions, 7 each for assessing anxiety and depression (Spahn *et al.* 2013). A HADS score of greater than 11 is more defining of anxiety and depression, with a reduction of 3 points indicating a clinical improvement (Eyles *et al.* 2015b).

Sleep- The Pittsburgh Sleep Quality Index (PSQI) a 19 items questionnaire that measures 7 domains of sleep scored on a 0-3 scale over 1 month (Buysse *et al.* 1989). The questionnaire gives a score that ranges from 0-21 with the higher the score the worse the sleep quality (Yu *et al.* 2020).

Mindfulness- The 24 item Five Facet Mindfulness Questionnaire Short Form (FFMQ-SF) is a 5 point Likert Scale which assess mindfulness in daily life. It measures 5 sub-scales: observing, describing, acting with awareness, non-judgement of inner experience and non-reacting to inner experience. The higher the score the greater the mindfulness (Compen *et al.* 2015; Johns *et al.* 2016)

Participants exercise behaviour will be assessed using the International physical activity questionnaire short form (IPAR-Q -SF). This questionnaire has 7 questions which assess the intensity, frequency and duration of exercise that participants engaged in over the last week.

Interventions

A brief description of the interventions of exercise and mindfulness can be found in (appendix 8). The operationalise of the COM-B model with the interventions for this study can also be found in (appendix 9).

Outcomes

Primary Outcome

The feasibility and acceptability of the study will be assessed in line with the MRC guidance on Developing and evaluating complex interventions (Craig 2019). These outcomes will assess participants that have completed or take part in the intervention part of the study and will be the following:

1. Adherence- interventions will run over 8 weeks. The completion of 4 weeks for mindfulness and 30min of home-based exercise weekly for two thirds (67%) of the duration of the trial will be regarded as adherence to the program. These levels of adherence are based on previous studies that have used the same levels (Mock *et al.* 2005; Pinto *et al.* 2005; Kim *et al.* 2019; Park *et al.* 2020) This will be assessed through the electronic diaries/logs that participants will be encouraged to complete along with the website/app data information- on frequency and duration of participant engagement with each session.
2. Attrition-We will estimate, following a review of web-based mindfulness intervention studies that the attrition rate will be approximately 20% with 10% to 15% of missing data (Carlson *et al.* 2019; Subnis *et al.* 2020).
3. Time to recruit, recruitment and retention rates, reasons for non-participation and acceptability of data collection. Time to recruit, recruitment and retention rates will be gathered through administrative data during (pre)screening, enrolment, allocation and follow-up. Reasons for non-participation, intervention/study dropouts will be collected when possible. Acceptability of data collection will be retrieved through missing data within questionnaires.
4. Acceptability of the programme will be assessed using focus groups this will include participants experience on the virtual delivery and data collection along with their experience and reflections on the exercise and mindfulness components of the intervention it will be achieved in the following way:

Design

Qualitative data collection utilising online focus groups will be facilitated via the Microsoft Teams (MS Teams) interface. This format for focus group has advantages including less dropout, more relaxed participants resulting in better communication, a more diverse group as able to include participants that are further away and cost-effective (Halliday *et al.* 2021)

Participants

Group sizes can vary and can be between 4 and 12 for focus groups (Barbour 2008). For online focus groups, a more realistic number that has been suggested by the literature would be a maximum of 6 per group (Holloway 2005; Halliday *et al.* 2021). The number and size of groups will also be dependent on trial recruitment numbers and continue until data saturation, which will be reached when no new information is generated (Goei *et al.* 2020). Separate groups will take place for each intervention group to ensure that each group can share and reflect on their similar experience of the intervention they took part in.

Materials

Once participants have completed the 8 weeks of the intervention, they will be invited to take part in online focus groups. An e-mail invitation (Appendix 10) will be sent along with a participant information sheet (Appendix 11) about the focus group. A follow-up phone call will be made by the researcher. Interested participants will be sent a confirmation e-mail outlining a suitable time and date. Confirmation of attendance will be requested along with consent prior to the focus group taking part online. If consent (Appendix 12) has not been received before the focus group, it will still be possible to achieve consent on the day of the focus group. This will be achieved by allowing the participants to enter the online focus group on Microsoft Teams (MS Teams) into a separate room and the consent process will occur before allowing participants to be allowed into the main focus group. A semi-structured interview guide (Appendix 13) shaped by relevant literature was developed which contained open-ended questions that reflected the objectives of the study. Probes will be used to elicit additional information when required. As the groups progress, the prompts will be developed to ensure that the phenomenon is thoroughly explored.

Procedure

Participants who agree to take part will be allocated to groups, these will be mixed groups of age, cancer type and stage but all participants will have completed the same intervention. To ensure that groups retain participants from the same intervention, participants will be asked by the research team through e-mail to provide the research team with information of which group they were randomised to. The allocation of groups will be made by the research team prior to focus groups taking place. If group numbers are too large, small or unevenly distributed then a decision within the research group will be reached regarding on reallocation of participants to groups. Groups will take place as close to the completion of the intervention as is practical. The groups will be facilitated by 3 researchers one of whom will be a facilitator and will ensure that the discussion stays on topic and equal time is shared amongst participants. The other will be responsible for technical support and will support participants during the session, record the session and take notes. The 3^rd^ researcher will be present in case any participant becomes distressed and will need to be removed from the focus group. If participation in these focus groups causes distress for any participants, a distress protocol (appendix 15) is in place to manage this. The PhD researcher, Kairen McCloy, has undertaken training with Ulster University on conducting focus groups but will be supported by Dr Jackie Gracey and team in undertaking these focus groups. Participants will be informed that the sessions will be recorded and if possible asked to keep audio and video on as this will aid the flow of conversation and will allow the facilitator to take field notes to capture both verbal and non-verbal communication. All focus group discussion will be audio and video recorded, then transcribed verbatim for analysis (Barbour 2008)

Analysis of data from focus groups will be done using Thematic Analysis. This type of analysis is flexible and allows for the in-depth identification and interpretation of themes. (Holloway and Galvin 2016; Castleberry and Nolen 2018) The phases of the analysis will include: familiarising with data, generating initial codes, searching for themes, reviewing themes, defining and naming themes and integrating these themes into a final report for publication (Braun and Clarke 2006).

Secondary Outcomes

Will include fatigue, QOL, sleep, anxiety and depression and mindfulness all of which will be assessed using self-report measures as described earlier.

Participant timeline

Please see Appendix 14.

Timeline for Study

| Task | Months 2021-2023 | | | | | | | | | | | | | |
| --- | --- | --- | --- | --- | --- | --- | --- | --- | --- | --- | --- | --- | --- | --- |
|  | Sep-Dec | | | | Jan-Mar | | Apr- Jun | | Jul-Sep | Oct-Dec | | | Jan-Mar | Apr-Jun |
| Peer review |  |  | | |  | |  | |  |  | | |  |  |
| Filter committee |  |  | |  |  | |  | |  |  | | |  |  |
| UU REC |  | |  | |  | |  | |  |  | |  | |  |
| Mobile App Development |  | | | |  |  |  | |  |  | |  | |  |
| Intervention group recruitment |  | | | |  |  |  | |  |  | |  | |  |
| Focus groups |  | | | |  | |  |  |  |  | |  | |  |
| Data analysis |  | | | |  | |  | |  |  |  |  | |  |

Ethics and dissemination

Ethical approval will be sought from the Nursing/Health Science filter Committee and University of Ulster Research Ethics Committee (UREC).

The findings of the research will be communicated with those who participated in the study either via email or a local event. The researcher will disseminate the projects findings and outputs via oral and poster presentations at local, national and international conferences and publications in peer reviewed journal papers.

Confidentiality

Governance of Data for intervention part of study:

Data will be anonymised, and stored securely. Databases will be encrypted, as will any transferred data. The key to the participant IDs will be held by the Chief Investigator. Access to the anonymised data will use authentication and be traceable by login details.

If a person withdraws from the study their data will kept for 10 years, participants will consent to this (appendix 5). Personal data will only be retained for the duration of the study. Further information on data handling can be found at:

<https://www.ulster.ac.uk/__data/assets/pdf_file/0003/75639/DataHandling.pdf>

This is a self-management project. As such, access to the app will be via download. This app is a protype that will be developed in collaboration with the School of Computing at Ulster University. Pilot testing of this app will take place with the 2 Patient and Public Representatives and an additional 3 service users resulting in 5 testers altogether.

Data from the app will be uploaded securely to a secure server housed in the Data centre at Ulster University Coleraine Campus.

**Care of Personal Information**

Participants will be allocated a unique code so that anonymity is protected**. All data and questionnaire responses will be anonymised.**

Data will be kept on password-protected computers that can only be accessed by the research team.

If permission is granted and the data is useful then anonymised data will be made available to other researchers via an ‘open’ repository at the end of the study.

Data security: Data will be stored for 10 years. Anonymised data (e.g. participant ID of the form A0001) will be held securely on an Ulster-based repository. Further information on this repository can be found at:

<http://uir-data.ulster.ac.uk/policy.html>

Governance of data for the focus groups part of the study:

Participants will be made aware that confidentiality will be maintained but the research team will be unable to prevent other members that participate in online focus groups and to adhere to this confidentiality agreement. Participants will also be informed that if they withdraw from this part of the study that once data has been anonymised it may be difficult to remove it.

Further information can be found at:

https://www.ulster.ac.uk/about/governance/compliance/gdpr

References.

*Gynaecological Cancer Research Charity.* (2020) Available at: <https://eveappeal.org.uk/> [Accessed Apr 22, 2021].

Al Maqbali, M. (2019) Exercise Interventions to Manage Fatigue in Women With Gynecologic Cancer: A Systematic Review. *Oncology Nursing Forum,* 46(1), 71-82.

Al Maqbali, M., Hughes, C., Gracey, J., Rankin, J., Dunwoody, L. and Hacker, E. (2019) Quality assessment criteria: psychometric properties of measurement tools for cancer related fatigue. *Acta Oncologica,* 58(9), 1286-1297.

Barbour, R.S. (2008) *Doing Focus Groups.* London: SAGE Publications. Available at: <http://ebookcentral.proquest.com/lib/ulster/detail.action?docID=783519>

Basen-Engquist, K., Carmack, C., Brown, J., Jhingran, A., Baum, G., Song, J., Scruggs, S., Swartz, M.C., Cox, M.G. and Lu, K.H. (2014) Response to an exercise intervention after endometrial cancer: Differences between obese and non-obese survivors. *Gynecologic Oncology,* 133(1), 48-55.

Blaney, J.M., Lowe-Strong, A., Rankin-Watt, J., Campbell, A. and Gracey, J.H. (2013) Cancer survivors' exercise barriers, facilitators and preferences in the context of fatigue, quality of life and physical activity participation: a questionnaire-survey. *Psycho-Oncology,* 22(1), 186-194.

Bower, J.E. (2014) Cancer-related fatigue—mechanisms, risk factors, and treatments. *Nature Reviews Clinical Oncology,* 11(10), 597.

Brandenbarg, D., Korsten, J. H. W. M., Berger, M.Y. and Berendsen, A.J. (2018) The effect of physical activity on fatigue among survivors of colorectal cancer: a systematic review and meta-analysis. *Supportive Care in Cancer,* 26(2), 393-403.

Braun and Clarke. (2006) Using thematic analysis in psychology. *Qualitative Research in Psychology,* 3(2), 77-101.

Bruggeman-Everts, F., Wolvers, M.D.J., van de Schoot, R., Vollenbroek-Hutten, M. and Van der Lee, Marije L. (2015) Effectiveness of Two Web-Based Interventions for Chronic Cancer-Related Fatigue Compared to an Active Control Condition: Results of the "Fitter na kanker" Randomized Controlled Trial. *Journal of Medical Internet Research,* 19(10)

Buysse, D.J., Reynolds III, C.F., Monk, T.H., Berman, S.R. and Kupfer, D.J. (1989) The Pittsburgh Sleep Quality Index: a new instrument for psychiatric practice and research. *Psychiatry Research,* 28(2), 193-213.

Carlson, L.E. and Garland, S.N. (2005) Impact of Mindfulness-Based Stress Reduction (MBSR) on sleep, mood, stress and fatigue symptoms in cancer outpatients. *International Journal of Behavioral Medicine,* 12(4), 278-285.

Carlson, L.E., Subnis, U. B. ( 1 ), Piedalue, K. -A. L. ( 1 ), Vallerand, J. ( 1 ), Speca, M. ( 1 ), Lupichuk, S. ( 1 ), Tang, P. ( 1 ), Faris, P. ( 2 ) and Wolever, R. Q. ( 3 ). (2019) The ONE-MIND Study: Rationale and protocol for assessing the effects of ONlinE MINDfulness-based cancer recovery for the prevention of fatigue and other common side effects during chemotherapy. *European Journal of Cancer Care,* 28(4)

Castleberry, A. and Nolen, A. (2018) Thematic analysis of qualitative research data: Is it as easy as it sounds? *Currents in Pharmacy Teaching and Learning,* 10(6), 807-815.

Cella, D.F., Tulsky, D.S., Gray, G., Sarafian, B., Linn, E., Bonomi, A., Silberman, M., Yellen, S.B., Winicour, P. and Brannon, J. (1993) The Functional Assessment of Cancer Therapy scale: development and validation of the general measure. *J Clin Oncol,* 11(3), 570-579.

Cillessen, L., Johannsen, M., Speckens, A.E.M. and Zachariae, R. (2019) Mindfulness-based interventions for psychological and physical health outcomes in cancer patients and survivors: A systematic review and meta-analysis of randomized controlled trials. *Psycho-Oncology,* 28(12), 2257-2269.

Clifford, B.K., Mizrahi, D., Sandler, C.X., Barry, B.K., Simar, D., Wakefield, C.E. and Goldstein, D. (2018) Barriers and facilitators of exercise experienced by cancer survivors: a mixed methods systematic review. *Supportive Care in Cancer,* 26(3), 685-700.

Cohen, J.A., Shumay, D.M., Chesney, M.A., Goyal, N., Barulich, M. and Levin, A.O. (2019) Survivorship Wellness: Insights from an Interdisciplinary Group-Based Survivorship Pilot Program at a Comprehensive Cancer Center. *Journal of Alternative and Complementary Medicine,* 25(7), 678-680.

Coleman, E.A., Goodwin, J.A., Kennedy, R., Coon, S.K., Richards, K., Enderlin, C., Stewart, C.B., McNatt, P., Lockhart, K. and Anaissie, E.J. (2012) Effects of Exercise on Fatigue, Sleep, and Performance: A Randomized Trial. *Oncology Nursing Forum,* 39(5), 468-477.

Compen, F.R., Bisseling, E.M. ( 1,2 ), Speckens, A. E. M. ( 1 ), Van der Lee, M. L. ( 2 ), Adang, E. M. M. ( 3 ) and Donders, A. R. T. ( 3 ). (2015) Study protocol of a multicenter randomized controlled trial comparing the effectiveness of group and individual internet-based Mindfulness-Based Cognitive Therapy with treatment as usual in reducing psychological distress in cancer patients: The BeMind study. *BMC Psychology,* 3(1)

Cowdell, F. and Dyson, J. (2019) How is the theoretical domains framework applied to developing health behaviour interventions? A systematic search and narrative synthesis. *BMC Public Health,* 19(1), 1-10.

Craig, P.e.a. (2019) Medical Research Council Developing and
evaluating complex
interventions.

Cramer, H., Lauche, R., Paul, A. and Dobos, G. (2012) Mindfulness-based stress reduction for breast cancer-a systematic review and meta-analysis. *Current Oncology,* 19(5), E343-E352.

Cramp, F. and Byron‐Daniel, J. (2012) Exercise for the management of cancer‐related fatigue in adults. *Cochrane Database of Systematic Reviews,* (11)

de Raaf, P.J., de Klerk, C. and van der Rijt, C. C. D. (2013) Elucidating the behavior of physical fatigue and mental fatigue in cancer patients: a review of the literature. *Psycho-Oncology,* 22(9), 1919-1929.

Dennett, A.M., Peiris, C.L., Shields, N., Prendergast, L.A. and Taylor, N.F. (2016) Moderate-intensity exercise reduces fatigue and improves mobility in cancer survivors: a systematic review and meta-regression. *Journal of Physiotherapy,* 62(2), 68-82.

Donnelly, C.M., Blaney, J.M., Lowe-Strong, A., Rankin, J.P., Campbell, A., McCrum-Gardner, E. and Gracey, J.H. (2011) A randomised controlled trial testing the feasibility and efficacy of a physical activity behavioural change intervention in managing fatigue with gynaecological cancer survivors. *Gynecologic Oncology,* 122(3), 618-624.

Eyles, C., Leydon, G.M., Hoffman, C.J., Copson, E.R., Prescott, P., Chorozoglou, M. and Lewith, G. (2015a) Mindfulness for the Self-Management of Fatigue, Anxiety, and Depression in Women With Metastatic Breast Cancer: A Mixed Methods Feasibility Study. *Integrative Cancer Therapies,* 14(1), 42-56.

Eyles, C., Leydon, G.M., Hoffman, C.J., Copson, E.R., Prescott, P., Chorozoglou, M. and Lewith, G. (2015b) Mindfulness for the Self-Management of Fatigue, Anxiety, and Depression in Women With Metastatic Breast Cancer: A Mixed Methods Feasibility Study. *Integrative Cancer Therapies,* 14(1), 42-56.

Gho, S.A., Munro, B.J., Jones, S.C. and Steele, J.R. (2014) Perceived Exercise Barriers Explain Exercise Participation in Australian Women Treated for Breast Cancer Better Than Perceived Exercise Benefits. *Physical Therapy,* 94(12), 1765-1774.

Goei, L.P.S., Lopez, V. and Klainin-Yobas, P. (2020) *Exploring the perceptions of cancer survivors of a mindfulness intervention at a tertiary hospital in Singapore: a descriptive qualitative study.*

Haller, H., Winkler, M.M., Klose, P., Dobos, G., Kümmel, S. and Cramer, H. (2017) Mindfulness-based interventions for women with breast cancer: an updated systematic review and meta-analysis. *Acta Oncologica,* 56(12), 1665-1676.

Halliday, M., Mill, D., Johnson, J. and Lee, K. (2021) Let's talk virtual! Online focus group facilitation for the modern researcher. *Research in Social and Administrative Pharmacy,*

Hardcastle, S.J., Maxwell-Smith, C., Kamarova, S., Lamb, S., Millar, L. and Cohen, P.A. (2018) Factors influencing non-participation in an exercise program and attitudes towards physical activity amongst cancer survivors. *Supportive Care in Cancer,* 26(4), 1289-1295.

Holloway, I. (2005) *EBOOK: Qualitative Research in Health Care.* Maidenhead: McGraw-Hill Education. Available at: <http://ebookcentral.proquest.com/lib/ulster/detail.action?docID=290402>

Holloway, I. and Galvin, K. (2016) *Qualitative Research in Nursing and Healthcare.* Hoboken: John Wiley & Sons, Incorporated.

Howlett, N., Schulz, J. ( 1 ), Trivedi, D. ( 1 ), Troop, N. ( 1 ) and Chater, A. ( 1,2,3 ). (2019) A prospective study exploring the construct and predictive validity of the COM-B model for physical activity. *Journal of Health Psychology,* 24(10), 1378-1391.

Huang, H.-., He, M., Wang, H.-. and Zhou, M. (2016) A meta-analysis of the benefits of mindfulness-based stress reduction (MBSR) on psychological function among breast cancer (BC) survivors. *Breast Cancer,* 23(4), 568-576.

Huang, J. and Shi, L. (2016) The effectiveness of mindfulness-based stress reduction (MBSR) for survivors of breast cancer: Study protocol for a randomized controlled trial. *Trials,* 17(1)

Johns, S.A., Brown, L.F., Beck-Coon, K., Monahan, P.O., Tong, Y., de Groot, M., Schmidt, K., Monceski, D., Danh, M., Kroenke, K., Talib, T.L., Carpenter, J.S., Von Ah, D., Giesler, R.B., Wilhelm, L., Wagner, C.D., Alyea, J.M. and Miller, K.D. (2016) Randomized controlled pilot trial of mindfulness-based stress reduction compared to psychoeducational support for persistently fatigued breast and colorectal cancer survivors. *Supportive Care in Cancer,* 24(10), 4085-4096.

Johns, S.A., Brown, L.F., Monahan, P.O., Tong, Y., Kroenke, K. and Beck-Coon, K. (2015) Randomized controlled pilot study of mindfulness-based stress reduction for persistently fatigued cancer survivors. *Psycho-Oncology,* 24(8), 885-893.

Johns, Von Ah, D., Brown, L.F., Beck-Coon, K., Talib, T.L., Alyea, J.M., Monahan, P.O., Tong, Y., Wilhelm, L. and Giesler, R.B. (2016) Randomized controlled pilot trial of mindfulness-based stress reduction for breast and colorectal cancer survivors: effects on cancer-related cognitive impairment. *Journal of Cancer Survivorship,* 10(3), 437‐448. Available at: <https://www.cochranelibrary.com/central/doi/10.1002/central/CN-01307710/full>

Joly, F., Ahmed-Lecheheb, D., Kalbacher, E., Heutte, N., Clarisse, B., Grellard, J.M., Gernier, F., Berton-Rigaud, D., Tredan, O., Fabbro, M., Savoye, A.M., Kurtz, J.E., Alexandre, J., Follana, P., Delecroix, V., Dohollou, N., Roemer-Becuwe, C., De Rauglaudre, G., Lortholary, A., Prulhiere, K., Lesoin, A., Zannetti, A., N'Guyen, S., Trager-Maury, S., Chauvenet, L., Lacourtoisie, S.A., Gompel, A., Lhomme, C., Floquet, A. and Pautier, P. (2019) Long-term fatigue and quality of life among epithelial ovarian cancer survivors: a GINECO case/control VIVROVAIRE I study. *Annals of Oncology,* 30(5), 845-852.

Juvet, L.K., Thune, I., Elvsaas, I.K.Ø, Fors, E.A., Lundgren, S., Bertheussen, G., Leivseth, G. and Oldervoll, L.M. (2017) The effect of exercise on fatigue and physical functioning in breast cancer patients during and after treatment and at 6 months follow-up: A meta-analysis. *The Breast,* 33, 166-177.

Keogh, J.W.L. and MacLeod, R.D. (2012) Body Composition, Physical Fitness, Functional Performance, Quality of Life, and Fatigue Benefits of Exercise for Prostate Cancer Patients: A Systematic Review. *Journal of Pain and Symptom Management,* 43(1), 96-110.

Kim, J.Y., Lee, M.K., Min, J.H., Jeon, J.Y., Lee, D.H., Kang, D.W., Lee, J.W., Chu, S.H., Cho, M.S. and Kim, N.K. (2019) Effects of a 12-week home-based exercise program on quality of life, psychological health, and the level of physical activity in colorectal cancer survivors: a randomized controlled trial. *Supportive Care in Cancer,* 27(8), 2933-2940.

Lavallee, J.F., Abdin, S., Faulkner, J. and Husted, M. (2018) Barriers and facilitators to participating in physical activity for adults with breast cancer receiving adjuvant treatment: A qualitative metasynthesis. *Psycho-Oncology,* 28(3), 468-476.

Lengacher, Reich, Post-White, Moscoso, Shelton, Barta, Le and Budhrani. (2012) Mindfulness based stress reduction in post-treatment breast cancer patients: an examination of symptoms and symptom clusters. *Journal of Behavioral Medicine,* 35(1), 86-94.

Lengacher, Reich, Ramesar, Alinat, Moscoso, Cousin, Marino, Elias, Paterson, Pleasant, Rodriguez, Wang, Kip, Meng and Park. (2017) Feasibility of the mobile mindfulness-based stress reduction for breast cancer (mMBSR(BC)) program for symptom improvement among breast cancer survivors. *Psycho-Oncology,* 27(2), 524-531.

Leydon, G.M., Eyles, C. and Lewith, G.T. (2012) A mixed methods feasibility study of mindfulness meditation for fatigue in women with metastatic breast cancer. *European Journal of Integrative Medicine,* 4(4), e429-e435.

Lin, K., Frawley, H.C., Denehy, L., Feil, D. and Granger, C.L. (2016) Exercise interventions for patients with gynaecological cancer: a systematic review and meta-analysis. *Physiotherapy,* 102(4), 309-319.

Lucas, A. R. ( 1 ), Focht, B. C. ( 2 ), Cohn, D. E. ( 3 ), Klatt, M. D. ( 3 ) and Buckworth, J. ( 4 ). (2017) A Mindfulness-Based Lifestyle Intervention for Obese, Inactive Endometrial Cancer Survivors: A Feasibility Study. *Integrative Cancer Therapies,* 16(3), 263-275.

Ma, Y., He, B., Jiang, M., Yang, Y., Wang, C., Huang, C. and Han, L. (2020) Prevalence and risk factors of cancer-related fatigue: A systematic review and meta-analysis. *International Journal of Nursing Studies,* 111

McMillan, E.M. and Newhouse, I.J. (2011) Exercise is an effective treatment modality for reducing cancer-related fatigue and improving physical capacity in cancer patients and survivors: a meta-analysis. *Applied Physiology, Nutrition & Metabolism,* 36(6), 892-903.

Mizrahi, D., Broderick, C., Friedlander, M., Ryan, M., Harrison, M., Pumpa, K. and Naumann, F. (2015) An Exercise Intervention During Chemotherapy for Women With Recurrent Ovarian Cancer. *International Journal of Gynecological Cancer,* 25(6), 985-992.

Mock, V., Davidson, N.E., Cameron, L., Zawacki, K., Stewart, K.J., Frangakis, C., Podewils, L.J., Ropka, M.E., Pickett, M., Poniatowski, B., Cohen, G. and McCorkle, R. (2005) Exercise manages fatigue during breast cancer treatment: A randomized controlled trial. *Psycho-Oncology,* 14(6), 464-477.

Moonsammy, S.H., Guglietti, C.L., Kuk, J.L., Ritvo, P., Mina, D.S., Ferguson, S., Urowitz, S. and Wiljer, D. (2013) A pilot study of an exercise & cognitive behavioral therapy intervention for epithelial ovarian cancer patients. *Journal of Ovarian Research,* 6(1)

Mugele, H., Freitag, N., Wilhelmi, J., Yang, Y., Cheng, S., Bloch, W. and Schumann, M. (2019) High-intensity interval training in the therapy and aftercare of cancer patients: a systematic review with meta-analysis. *Journal of Cancer Survivorship,* 13(2), 205-223.

Naraphong, W., Lane, A., Schafer, J., Whitmer, K. and Wilson, B.R.A. (2015) Exercise intervention for fatigue-related symptoms in Thai women with breast cancer: A pilot study. *Nursing and Health Sciences,* 17(1), 33-41.

Oh, H.S. and Seo, W.S. (2011) Systematic Review and Meta-Analysis of the Correlates of Cancer-Related Fatigue. *Worldviews on Evidence-Based Nursing,* 8(4), 191-201.

Park, S., Sato, Y., Takita, Y., Tamura, N., Ninomiya, A., Kosugi, T., Sado, M., Nakagawa, A., Takahashi, M., Hayashida, T. and Fujisawa, D. (2020) Mindfulness-Based Cognitive Therapy for Psychological Distress, Fear of Cancer Recurrence, Fatigue, Spiritual Well-Being, and Quality of Life in Patients With Breast Cancer—A Randomized Controlled Trial. *Journal of Pain and Symptom Management,* 60(2), 381-389.

Payne, J.K., Held, J., Thorpe, J. and Shaw, H. (2008) Effect of exercise on biomarkers, fatigue, sleep disturbances, and depressive symptoms in older women with breast cancer receiving hormonal therapy. *Oncology Nursing Forum,* 35(4), 635-642.

Pinto, B.M., Frierson, G.M., Rabin, C., Trunzo, J.J. and Marcus, B.H. (2005) *Home-based physical activity intervention for breast cancer patients.*

Poort, H., de Rooij, B.H., Ezendam, N.P.M., van de Poll-Franse, L., Uno, H., Weng, S. and Wright, A.A. (2020) Patterns and predictors of cancer-related fatigue in ovarian and endometrial cancers: 1-year longitudinal study. *Cancer,* 126(15), 3526-3533.

Price-Blackshear, M., Pratscher, S. D. ( 1 ), Oyler, D. L. ( 1 ), Ann Bettencourt, B. ( 1 ), Armer, J. M. ( 2 ), Udmuangpia, T. ( 2 ), Cheng, A. -L. ( 3 ), Cheng, M. X. ( 4 ), Records, K. ( 5 ) and Carson, J. W. ( 6 ). (2020) Online couples mindfulness-based intervention for young breast cancer survivors and their partners: A randomized-control trial. *Journal of Psychosocial Oncology,* 38(5), 592-611.

Reich, R.R., Lengacher, C.A., Alinat, C.B., Kip, K.E., Paterson, C., Ramesar, S., Han, H.S., Ismail-Khan, R., Johnson-Mallard, V., Moscoso, M., Budhrani-Shani, P., Shivers, S., Cox, C.E., Goodman, M. and Park, J. (2017a) Mindfulness-Based Stress Reduction in Post-treatment Breast Cancer Patients: Immediate and Sustained Effects Across Multiple Symptom Clusters. *Journal of Pain & Symptom Management,* 53(1), 85-95.

Reich, R.R., Lengacher, C.A., Alinat, C.B., Kip, K.E., Paterson, C., Ramesar, S., Han, H.S., Ismail-Khan, R., Johnson-Mallard, V., Moscoso, M., Budhrani-Shani, P., Shivers, S., Cox, C.E., Goodman, M. and Park, J. (2017b) Mindfulness-Based Stress Reduction in Post-treatment Breast Cancer Patients: Immediate and Sustained Effects Across Multiple Symptom Clusters. *Journal of Pain & Symptom Management,* 53(1), 85-95.

Schuler, M.K., Hentschel, L., Kisel, W., Kramer, M., Lenz, F., Hornemann, B., Hoffmann, J., Richter, S., Ehninger, G., Bornhäuser, M. and Kroschinsky, F. (2017) Impact of Different Exercise Programs on Severe Fatigue in Patients Undergoing Anticancer Treatment—A Randomized Controlled Trial. *Journal of Pain and Symptom Management,* 53(1), 57-66.

Sekse, R.J.T., Hufthammer, K.O. and Vika, M.E. (2015) Fatigue and quality of life in women treated for various types of gynaecological cancers: a cross-sectional study. *Journal of Clinical Nursing,* 24(3-4), 546-555.

Sheehan, P., Denieffe, S., Murphy, N.M. and Harrison, M. (2020a) Exercise is more effective than health education in reducing fatigue in fatigued cancer survivors. *Supportive Care in Cancer : Official Journal of the Multinational Association of Supportive Care in Cancer,* 28(10), 4953-4962.

Sheehan, P., Denieffe, S., Murphy, N.M. and Harrison, M. (2020b) Exercise is more effective than health education in reducing fatigue in fatigued cancer survivors. *Supportive Care in Cancer : Official Journal of the Multinational Association of Supportive Care in Cancer,* 28(10), 4953-4962.

Smits, A., Lopes, A., Das, N., Bekkers, R. and Galaal, K. (2014) The impact of BMI on quality of life in obese endometrial cancer survivors: Does size matter? *Gynecologic Oncology,* 132(1), 137-141.

Spahn, G., Choi, K., Kennemann, C., Lüdtke, R., Franken, U., Langhorst, J., Paul, A. and Dobos, G.J. (2013) Can a Multimodal Mind–Body Program Enhance the Treatment Effects of Physical Activity in Breast Cancer Survivors With Chronic Tumor-Associated Fatigue? A Randomized Controlled Trial. *Integrative Cancer Therapies,* 12(4), 291-300.

Stelten, S., Hoedjes, M., Kenter, G.G., Kampman, E., Huijsmans, R.J., van Lonkhuijzen, L.R. and Buffart, L.M. (2020) Rationale and study protocol of the Physical Activity and Dietary intervention in women with OVArian cancer (PADOVA) study: a randomised controlled trial to evaluate effectiveness of a tailored exercise and dietary intervention on body composition, physical function and fatigue in women with ovarian cancer undergoing chemotherapy. *BMJ Open,* 10(11), e036854.

Subnis, U.B., Farb, N.A.S., Piedalue, K.L., Speca, M., Lupichuk, S., Tang, P.A., Faris, P., Thoburn, M., Saab, B.J. and Carlson, L.E. (2020) A Smartphone App–Based Mindfulness Intervention for Cancer Survivors: Protocol for a Randomized Controlled Trial. *JMIR Research Protocols,* 9(5), e15178.

Tian, L., Lu, H., Lin, L., Hu, Y. and Lu, H.J. (2016) Effects of aerobic exercise on cancer-related fatigue: a meta-analysis of randomized controlled trials. *Supportive Care in Cancer,* 24(2), 969-983.

Van Dam, N.,T., van Vugt, M.,K., Vago, D.R., Schmalzl, L., Saron, C.D., Olendzki, A., Meissner, T., Lazar, S.W., Kerr, C.E., Gorchov, J., Fox, K.C.R., Field, B.A., Britton, W.B., Brefczynski-Lewis, J. and Meyer, D.E. (2018) Mind the Hype: A Critical Evaluation and Prescriptive Agenda for Research on Mindfulness and Meditation. *Perspectives on Psychological Science : A Journal of the Association for Psychological Science,* 13(1), 36-61.

Van Der Lee, and Garssen. (2012) Mindfulness-based cognitive therapy reduces chronic cancer-related fatigue: A treatment study. *Psycho-Oncology,* 21(3), 264-272.

Velthuis, M.J., Agasi-Idenburg, S., Aufdemkampe, G. and Wittink, H.M. (2010) The Effect of Physical Exercise on Cancer-related Fatigue during Cancer Treatment: a Meta-analysis of Randomised Controlled Trials. *Clinical Oncology,* 22(3), 208-221.

Victorson, D., Hankin, V., Burns, J., Weiland, R., Maletich, C., Sufrin, N., Schuette, S., Gutierrez, B. and Brendler, C. (2017) Feasibility, acceptability and preliminary psychological benefits of mindfulness meditation training in a sample of men diagnosed with prostate cancer on active surveillance: results from a randomized controlled pilot trial. *Psycho-Oncology,* 26(8), 1155-1163.

von Gruenigen, V.E., Gibbons, H.E. ( 1,2 ), Janata, J.W. ( 1,2,4 ), Kavanagh, M. B. ( 3 ), Lerner, E. ( 3 ) and Courneya, K. S. ( 5 ). (2009) A randomized trial of a lifestyle intervention in obese endometrial cancer survivors: Quality of life outcomes and mediators of behavior change. *Health and Quality of Life Outcomes,* 7

Wang, Y.-., Boehmke, M., Wu, Y.-.B., Dickerson, S.S. and Fisher, N. (2011) Effects of a 6-week walking program on Taiwanese women newly diagnosed with early-stage breast cancer. *Cancer Nursing,* 34(2), E1-E13.

Wenzel, J.A., Griffith, K.A., Shang, J., Thompson, C.B., Hedlin, H., Stewart, K.J., DeWeese, T. and Mock, V. (2013) Impact of a home-based walking intervention on outcomes of sleep quality, emotional distress, and fatigue in patients undergoing treatment for solid tumors. *The Oncologist,* 18(4), 476.

Xie, C., Dong, B., Wang, L., Jing, X., Wu, Y., Lin, L. and Tian, L. (2020) Mindfulness-based stress reduction can alleviate cancer- related fatigue: A meta-analysis. *Journal of Psychosomatic Research,* 130

Xunlin, N.G., Lau, Y. and Klainin-Yobas, P. (2020) The effectiveness of mindfulness-based interventions among cancer patients and survivors: a systematic review and meta-analysis. *Supportive Care in Cancer,* 28(4), 1563-1578.

Yu, C.-., Wang, T.-., Liang, S.-., Wu, S.-., Lu, Y.Y., Chang, C.-. and Liu, C.-. (2020) Healthy life styles, sleep and fatigue in endometrial cancer survivors: A cross-sectional study. *Journal of Clinical Nursing,* 29(7-8), 1372-1380.

Zhang, Q., Li, F., Zhang, H., Yu, X. and Cong, Y. (2018) Effects of nurse-led home-based exercise & cognitive behavioral therapy on reducing cancer-related fatigue in patients with ovarian cancer during and after chemotherapy: A randomized controlled trial. *International Journal of Nursing Studies,* 78, 52-60.

Zhang, Q., Zhao, H. and Zheng, Y. (2019) Effectiveness of mindfulness-based stress reduction (MBSR) on symptom variables and health-related quality of life in breast cancer patientsa systematic review and meta-analysis. *Supportive Care in Cancer,* 27(3), 771-781.

Zigmond, A.S. and Snaith, R.P. (1983) The hospital anxiety and depression scale. *Acta Psychiatrica Scandinavica,* 67(6), 361-370.

Zou, L.-., Yang, L., He, X.-., Sun, M. and Xu, J.-. (2014) Effects of aerobic exercise on cancer-related fatigue in breast cancer patients receiving chemotherapy: A meta-analysis. *Tumor Biology,* 35(6), 5659-5667.

stylefix

**Statistical Model**

**Outcome measures**

Primary Outcome

The feasibility and acceptability of the study will be assessed in line with the MRC guidance on Developing and evaluating complex interventions (Craig 2019). These outcomes will assess participants that have completed or take part in the intervention part of the study and will be the following:

1. Adherence- interventions will run over 8 weeks. The completion of 4 weeks for mindfulness and 30min of home-based exercise weekly for two thirds (67%) of the duration of the trial will be regarded as adherence to the program. These levels of adherence are based on previous studies that have used the same levels (Mock *et al.* 2005; Pinto *et al.* 2005; Kim *et al.* 2019; Park *et al.* 2020) This will be assessed through the electronic diaries/logs that participants will be encouraged to complete along with the website/app data information- on frequency and duration of participant engagement with each session.
2. Attrition-We will estimate, following a review of web-based mindfulness intervention studies that the attrition rate will be approximately 20% with 10% to 15% of missing data (Carlson *et al.* 2019; Subnis *et al.* 2020).
3. Time to recruit, recruitment and retention rates, reasons for non-participation and acceptability of data collection. Time to recruit, recruitment and retention rates will be gathered through administrative data during (pre)screening, enrolment, allocation and follow-up. Reasons for non-participation, intervention/study dropouts will be collected when possible. Acceptability of data collection will be retrieved through missing data within questionnaires.
4. Acceptability of the programme will be assessed using focus groups this will include participants experience on the virtual delivery and data collection along with their experience and reflections on the exercise and mindfulness components of the intervention. A detailed description of these focus groups can be found in the protocol page 8,9 & 10.

Secondary Outcomes

Will include fatigue, QOL, sleep, anxiety and depression and mindfulness all of which will be assessed using self-report measures which are described in detail in the protocol page 6 & 7 and appendix 6.

**Sample size**

The sample size for this study is based upon feasibility and review of similar studies. The literature demonstrates that feasibility studies with similar interventions and methodologies within the cancer population recruit on average 40 participants in total (Eyles *et al.* 2015; Victorson *et al.* 2017; Price-Blackshear *et al.* 2020). Therefore it is anticipated that this study will recruit a total of 40 participants with 20 in each arm of the study.

**Statistical methods**

Descriptive statistics will be performed to produce mean values and standard deviations. Groups will be compared at baseline using independent t-test (two independent samples) for continuous data, and the chi-square test for independence for categorical data.

Primary analyses will be performed using the intention to treat on all data and using imputation method where outcomes are missing. Alpha will be set a priori at a level of P = .05. Repeated-measures ANOVAs will then be used to determine the effects of exercise and mindfulness and mindfulness intervention on changes in all outcomes of interest.

As this is a feasibility study and preliminary efficacy study, effect sizes (Cohen’s d) will be calculated by taking the mean difference and dividing by the pooled standard deviation to better estimate the meaningfulness of change for each observed outcome following the intervention. The values and meanings for effect size estimates are a small effect size (d = .2), a moderate effect size (d = .5), and a large effect size (d = .8).

Corelation analysis will be performed to determine whether changes in mindfulness/exercise are associated with changes in fatigue, anxiety, depression or sleep.

References.

Carlson, L.E., Subnis, U. B. ( 1 ), Piedalue, K. -A. L. ( 1 ), Vallerand, J. ( 1 ), Speca, M. ( 1 ), Lupichuk, S. ( 1 ), Tang, P. ( 1 ), Faris, P. ( 2 ) and Wolever, R. Q. ( 3 ). (2019) The ONE-MIND Study: Rationale and protocol for assessing the effects of ONlinE MINDfulness-based cancer recovery for the prevention of fatigue and other common side effects during chemotherapy. *European Journal of Cancer Care,* 28(4)

Craig, P.e.a. (2019) Medical Research Council Developing and
evaluating complex
interventions.

Eyles, C., Leydon, G.M., Hoffman, C.J., Copson, E.R., Prescott, P., Chorozoglou, M. and Lewith, G. (2015) Mindfulness for the Self-Management of Fatigue, Anxiety, and Depression in Women With Metastatic Breast Cancer: A Mixed Methods Feasibility Study. *Integrative Cancer Therapies,* 14(1), 42-56.

Kim, J.Y., Lee, M.K., Min, J.H., Jeon, J.Y., Lee, D.H., Kang, D.W., Lee, J.W., Chu, S.H., Cho, M.S. and Kim, N.K. (2019) Effects of a 12-week home-based exercise program on quality of life, psychological health, and the level of physical activity in colorectal cancer survivors: a randomized controlled trial. *Supportive Care in Cancer,* 27(8), 2933-2940.

Mock, V., Davidson, N.E., Cameron, L., Zawacki, K., Stewart, K.J., Frangakis, C., Podewils, L.J., Ropka, M.E., Pickett, M., Poniatowski, B., Cohen, G. and McCorkle, R. (2005) Exercise manages fatigue during breast cancer treatment: A randomized controlled trial. *Psycho-Oncology,* 14(6), 464-477.

Park, S., Sato, Y., Takita, Y., Tamura, N., Ninomiya, A., Kosugi, T., Sado, M., Nakagawa, A., Takahashi, M., Hayashida, T. and Fujisawa, D. (2020) Mindfulness-Based Cognitive Therapy for Psychological Distress, Fear of Cancer Recurrence, Fatigue, Spiritual Well-Being, and Quality of Life in Patients With Breast Cancer—A Randomized Controlled Trial. *Journal of Pain and Symptom Management,* 60(2), 381-389.

Pinto, B.M., Frierson, G.M., Rabin, C., Trunzo, J.J. and Marcus, B.H. (2005) *Home-based physical activity intervention for breast cancer patients.*

Price-Blackshear, M., Pratscher, S. D. ( 1 ), Oyler, D. L. ( 1 ), Ann Bettencourt, B. ( 1 ), Armer, J. M. ( 2 ), Udmuangpia, T. ( 2 ), Cheng, A. -L. ( 3 ), Cheng, M. X. ( 4 ), Records, K. ( 5 ) and Carson, J. W. ( 6 ). (2020) Online couples mindfulness-based intervention for young breast cancer survivors and their partners: A randomized-control trial. *Journal of Psychosocial Oncology,* 38(5), 592-611.

Subnis, U.B., Farb, N.A.S., Piedalue, K.L., Speca, M., Lupichuk, S., Tang, P.A., Faris, P., Thoburn, M., Saab, B.J. and Carlson, L.E. (2020) A Smartphone App–Based Mindfulness Intervention for Cancer Survivors: Protocol for a Randomized Controlled Trial. *JMIR Research Protocols,* 9(5), e15178.

Victorson, D., Hankin, V., Burns, J., Weiland, R., Maletich, C., Sufrin, N., Schuette, S., Gutierrez, B. and Brendler, C. (2017) Feasibility, acceptability and preliminary psychological benefits of mindfulness meditation training in a sample of men diagnosed with prostate cancer on active surveillance: results from a randomized controlled pilot trial. *Psycho-Oncology,* 26(8), 1155-1163.

stylefix

Appendix 4

This study has been approved by the Ulster University Ethics Committee

Qualtrics link:

<https://ulsterhealth.eu.qualtrics.com/jfe/form/SV_5ckh2k14byHxMWy>

**Participant Information Sheet**

**Study Title:**

**Gynaecology Exercise and Mindfulness Study (GEMS) : A feasibility trial evaluating the effectiveness of mindfulness compared to mindfulness and exercise on fatigue in gynaecology cancer.**

You are being invited to take part in a research study. Before you decide whether or not to take part, it is important that you understand what the research is for and what you will be asked to do. Please read the following information and do not hesitate to ask any questions about anything that might not be clear to you. Make sure that you are happy before you decide what to do. Thank you for taking the time to consider this invitatio3n.

**What is the purpose of this study?**

Previous research has shown that exercise and mindfulness on their own can help reduce cancer related fatigue and improve sleep, depression and quality of life. However most of this research has mainly involved women with breast cancer and to date no research has used both mindfulness and exercise together in women with gynaecological cancer. This study is seeking to address this lack of research.

**Why have I been invited?**

You have been asked to take part in this study because you have been previously treated for gynaecology cancer and are still experiencing fatigue. You will be part of a group of approximately forty women taking part in this study.

**Do I have to take part in the study?**

It is up to you to decide whether or not to take part. If you do decide to take part, an electronic copy of this information sheet will be sent to you to keep. You can have a week to consider this and then we will ask you to sign an electronic consent form. This electronic consent form can be accessed via a link that will be e-mailed to you. If you choose to take part, you can change your mind at any time and withdraw from the study without giving a reason.

**What do I need to take part?**

To take part you will need internet access and a device either a smart phone, tablet (such as an iPad ) or other similar device that can access the app.

**What will happen to me if I take part?**

The study will last for 8 weeks in total. It will all take place on a mobile application that you will be able to download through an online web page that will be sent to you in a link. You will be asked to access this mobile application regularly at a time that suits you. To establish whether there is any difference in effect on fatigue we will compare a mindfulness group (group A) with a mindfulness and exercise group (group B). To do this you will be randomly assigned (by chance) into one of the two groups. This makes sure that each group has a similar mix of people and ensures that the results of the study are not biased for any reason.

**What will I have to do?**

All participants will be asked to complete electronic questionnaires, a link for these questionaries will be e-mailed to you, this will happen at the beginning and end of the programme. This will allow us to compare your initial fatigue, sleep, anxiety, depression and quality of life levels with those at the end and help us determine the overall effectiveness of the programme. The assessments will include questionnaires for fatigue, sleep, anxiety, depression and quality of life. These will be available to fill in on line through a link that will be sent to you at the beginning and end of the programme and will take approximately 30 minutes to complete.

All participants must be willing to be randomly assigned (by chance) to either the intervention group A (mindfulness only) or intervention group B (mindfulness and exercise).

**Once you are assigned to a group either** the mindfulness only programme, or the mindfulness and exercise group you will be sent a link to the e-mail address you have provided for the secure web page. Once you have clicked this link and registered you may now download the mobile application which will give you access to the material for this study. Each week new material will be released, you can access all the material, videos, audio recordings and written information when it suits you. We ask you to work through them and to complete the exercises as instructed. We would encourage you to complete all of the weeks exercises and read or watch all the material. The length of time to complete this will vary from 30 minutes to an hour depending on the weeks material and goals you have set. There is also an electronic log where we ask you to record whether you have completed your exercise and mindfulness, so how many days you were able to practice mindfulness and/or exercise and for how long. During your first week the researcher will contact you to ensure that you were able to access everything and there were no problems. You and your researcher will agree a suitable time to be contacted weekly via telephone to discuss progress and any difficulties encountered.

After the programme you will be invited to attend an online group discussion lasting **one hour to one and a half hours** establish your overall thoughts on the programme. This is entirely optional but will be an opportunity for you to put forward your opinions as to how the programme could be improved in the future and your opinions of taking part in the exercise or mindfulness activities. The group session will be recorded to allow for analysis at a later date.

**What are the possible disadvantages and risks of taking part?**

The main effects that you may experience may be sore muscles following exercise this is known as delayed onset muscle soreness and should resolve itself. If this does not or becomes worse please contact the research team for further advice. Mindfulness can sometimes result in unpleasant feelings such as anxiety if this occurs and you are finding it distressing please discontinue the mindfulness practice and contact the research team or alternatively you find additional support at the links below in the useful links section. The risks of taking part in the study should be minimal as you will be screened for your suitability prior to taking part and videos and written information will be available to show you how to exercise and practice mindfulness safely.

**What are the side effects of taking part?**

As outlined in the previous paragraph, it is highly unlikely that you will experience any side effects to taking part in the programme as your suitability to take part will be determined prior to the study commencing. You will be given information at the beginning on when not to exercise or take part in mindfulness and how to safely exercise or practice mindfulness. All this information will be available on the mobile application and you will be asked to contact the research team if you experience any side effects related to you exercising or participating in mindfulness.

**What are the possible benefits of taking part?**

Previous studies involving people diagnosed with cancer suggest that taking part in an exercise or mindfulness programmes helps:

- Decrease fatigue
- Maintain or improve physical functioning
- Improve quality of life
- Reduce anxiety or depression
- Improve sleep

**What will happen if I don’t want to carry on with the study?**

This is a voluntary study and you are free to withdraw at any time without giving reasons.

If you decide not to continue, you can withdraw completely from the study. However, it is helpful for us to understand why you no longer participated and with your permission we would like to contact you on one occasion to find out why you no longer want to continue if you would be happy to share this with us. Gathering data from all participants regardless of participation is important for our overall findings.

All data obtained from this study will be retained for a minimum of 10 years.

**What if there is a problem?**

If you should have a concern about any aspect of this study, you should ask to speak to the researchers who will do their best to answer your questions (Contact number: 07984843441). If you wish to complain formally, you can through Ulster University complaints procedure.

In the unlikely event that something does go wrong and you are harmed during the research due to someone’s negligence, then you may have grounds for legal action and seek compensation against Ulster University.

**Will my taking part in this study be kept confidential?**

Yes, the research team have a duty of confidentiality to you as a research participant. Ulster University is the sponsor or managing organisation for this study and we will use information gathered from you and/or your records in order to carry it out. We will act as the data controller, which means that we are responsible for looking after your information and using it properly, as stipulated in GDPR and the Data Protection Act 2018. Ulster University will keep identifiable information about you for 10 years after the study has finished. You can find out more about how we look after your information at:

https://www.ulster.ac.uk/about/governance/compliance/gdpr

Data obtained will be entered and managed in an anonymous format and we will hold your contact details on a separate database. Data will be stored in computers which are password protected. These computers are based at Ulster University in a secure environment. Only members of the research team will have access to identifiable data. Data obtained will be stored for a minimum of ten years within Ulster University.

If you wish to raise a complaint on how we have handled your personal data, you can contact our Data Protection Officer who will investigate the matter. If you are not satisfied with our response or believe we are processing your personal data in a way that is not lawful you can complain to the Information Commissioner’s Office (ICO). Our Data Protection Officer is Eamon Mullan; you can contact him at e.mullan@ulster.ac.uk.

**What will happen to the results of the research study?**

Upon completion of the trial you will be provided with your individual results from the study. Following this you will be provided with a summary leaflet of the main findings of the study which will be based on all participants involved in the trial. Upon the study being accepted for publication in a journal you will be provided with this information. You will not be identifiable from any report or publication.

**Who is organising and funding the research?**

The Department of Economy has funded a qualified nurse to undertake this study as part of their further educational qualification for a Doctorate of Philosophy (PhD). This study will be written up as part of the PhD thesis which will be held at Ulster University library.

**Who has reviewed the study?**

All research within Ulster University is looked at by an independent group of people called a Research Ethics Committee to protect your safety, rights, well-being and dignity. This study has been reviewed and given favourable opinion by Ulster University Research Ethics Committee.

**Further information**

If you require further information about this study or advice on whether to participate please see contact details below:

**If you would like more information about this study?**

Contact Kairen McCloy at xxxxxx or e-mail:

**Should I participate?**

It is your choice whether you wish to participate, you may wish to discuss your decision with your family, friends or a healthcare professional.

**If I am unhappy with the study who should I contact?**

If you are unhappy with any aspect of the study please contact either:

Project Investigator: Kairen McCloy Chief Investigator: Dr Jackie Gracey

Research Governance: Nick Curry at

**Links to organisations if require further support:**

<https://www.macmillan.org.uk/cancer-information-and-support/get-help/emotional-help>

<https://cancerfocusni.org/patient-support/>

<https://actioncancer.org/>

<https://ulsterhealth.eu.qualtrics.com/jfe/form/SV_5ckh2k14byHxMWy>

**Appendix 11**

**This study has been approved by Ulster University Ethics Committee**

**Qualtrics link:**

<https://ulsterhealth.eu.qualtrics.com/jfe/form/SV_6n7jGWDOydJjcQS>

**PARTICIPANT INFORMATION SHEET**

**GYNAECOLOGY EXERCISE AND MINDFULNESS STUDY (GEMS)**

**MINDFULNESS FOCUS GROUP STUDY**

You are being invited to take part in the above study. Before you decide, it is important for you to understand why the focus group is being done and what it will involve. Please take time to read the following information explaining the study. Do ask if there is anything that is not clear or of you would like more information.

**What is a focus grou**p?

A focus group is a type of study that involves participants who share a similar background or experience. It is a small group discussion (usually six people or less), aimed at gaining an insight and better understanding of participant’s views or feelings on a specific topic. Participants are encouraged to speak freely, while sharing their experience and opinions with others in a group discussion.

**What is the purpose of this focus group?**

This focus group wishes to explore your experiences within the mindfulness programme you have just taken part in, to determine how it could be improved for the better.

**Do I have to take part?**

Participation is entirely voluntary, and you will be free to withdraw from the study at any time and without explanation. A decision to withdraw at any time, or a decision not to take part, will not affect the standard of care you receive at any time.

**What does the study involve?**

The focus groups session will last **approximately 1-1½ hours** and will take place online so internet access and a device such as a smart phone or tablet will be required to take part. Here you will be asked to discuss your opinions of the mindfulness programme with other participants. Each session will be led by a group facilitator. There are no right or wrong answers, and you are encouraged to talk freely about your individual experiences and opinions.

The discussion will be audio and video recorded to allow the information to be accurately transcribed and the content analysed. All of the information obtained, will be kept confidential. Ulster University is the sponsor or managing organisation for this study and we will use information gathered from you and/or your records in order to carry it out. We will act as the data controller, which means that we are responsible for looking after your information and using it properly, as stipulated in GDPR and the Data Protection Act 2018. Ulster University will keep information about you for 10 years after the study has finished. You can find out more about how we look after your information at:

<https://www.ulster.ac.uk/about/governance/compliance/gdpr>

No participants will be identifiable by the study. Once the entire study is complete, all the participants will receive a copy of the key findings identified. The study will be documented as part of a PhD thesis and written up for publication in relevant peer review journals.

If you wish to raise a complaint on how we have handled your personal data, you can contact our Data Protection Officer who will investigate the matter. If you are not satisfied with our response or believe we are processing your personal data in a way that is not lawful you can complain to the Information Commissioner’s Office (ICO). Our Data Protection Officer is Eamon Mullan; you can contact him at e.mullan@ulster.ac.uk.

**What are the possible benefits of taking part?**

We hope that your experiences will inform future mindfulness programmes aimed at benefiting other women with gynaecological cancer.

**What are the possible disadvantages and risks of taking part?**

Sometimes talking about experiences may make you feel upset or anxious if this occurs please do not continue if you do not feel able. A member of the team will assist you and additional support can be found in the links below in useful links section.

**Who has reviewed the study?**

The study has been reviewed by Research Ethics Committee Ulster University.

Contact for further information

Researcher: Kairen McCloy Tel: xxxx e-mail:

Chief Investigator: Dr Jackie Gracey

**Links to organisations if require further support:**

<https://www.macmillan.org.uk/cancer-information-and-support/get-help/emotional-help>

<https://cancerfocusni.org/patient-support/>

<https://actioncancer.org/>

Qualtrics Link:

<https://ulsterhealth.eu.qualtrics.com/jfe/form/SV_1HcWPWfjhYtgFVk>

**PARTICIPANT INFORMATION SHEET**

**GYNAECOLOGY EXERCISE AND MINDFULNESS STUDY (GEMS)**

**EXERCISE AND MINDFULNESS FOCUS GROUP STUDY**

You are being invited to take part in the above study. Before you decide, it is important for you to understand why the focus group is being done and what it will involve. Please take time to read the following information explaining the study. Do ask if there is anything that is not clear or of you would like more information.

**What is a focus grou**p?

A focus group is a type of study that involves participants who share a similar background or experience. It is a small group discussion (usually six people or less), aimed at gaining an insight and better understanding of participant’s views or feelings on a specific topic. Participants are encouraged to speak freely, while sharing their experience and opinions with others in a group discussion.

**What is the purpose of this focus group?**

This focus group wishes to explore your experiences within the mindfulness and exercise programme you have just taken part in, to determine how it could be improved for the better.

**Do I have to take part?**

Participation is entirely voluntary, and you will be free to withdraw from the study at any time and without explanation. A decision to withdraw at any time, or a decision not to take part, will not affect the standard of care you receive at any time.

**What does the study involve?**

The focus groups session will last **approximately 1-1½ hours** and will take place online so internet access and a device such as a smart phone or tablet will be required to take part.**.** Here you will be asked to discuss your opinions of the mindfulness and exercise programme with other participants. Each session will be led by a group facilitator. There are no right or wrong answers, and you are encouraged to talk freely about your individual experiences and opinions.

The discussion will be audio and video recorded to allow the information to be accurately transcribed and the content analysed. All of the information obtained, will be kept confidential. Ulster University is the sponsor or managing organisation for this study and we will use information gathered from you and/or your records in order to carry it out. We will act as the data controller, which means that we are responsible for looking after your information and using it properly, as stipulated in GDPR and the Data Protection Act 2018. Ulster University will keep identifiable information about you for 10 years after the study has finished. You can find out more about how we look after your information at:

<https://www.ulster.ac.uk/about/governance/compliance/gdpr>

No participants will be identifiable by the study. Once the entire study is complete, all the participants will receive a copy of the key findings identified. The study will be documented as part of a PhD thesis and written up for publication in relevant peer review journals.

If you wish to raise a complaint on how we have handled your personal data, you can contact our Data Protection Officer who will investigate the matter. If you are not satisfied with our response or believe we are processing your personal data in a way that is not lawful you can complain to the Information Commissioner’s Office (ICO). Our Data Protection Officer is Eamon Mullan; you can contact him at e.mullan@ulster.ac.uk.

**What are the possible benefits of taking part?**

We hope that your experiences will inform future mindfulness and exercise programmes aimed at benefiting other women with gynaecological cancer.

**What are the possible disadvantages and risks of taking part?**

Sometimes talking about experiences may make you feel upset or anxious if this occurs please do not continue if you do not feel able. A member of the team will assist you and additional support can be found in the links below in useful links section.

**Who has reviewed the study?**

The study has been reviewed by Research Ethics Committee Ulster University.

Contact for further information

Researcher: Kairen McCloy Tel: xxxx e-mail:

Chief Investigator: Dr Jackie Gracey

**Links to organisations if require further support:**

<https://www.macmillan.org.uk/cancer-information-and-support/get-help/emotional-help>

<https://cancerfocusni.org/patient-support/>

<https://actioncancer.org/>

**Appendix 10**

Invitation e-mail to Participants following GEMS study.

Date

Dear,

**Gynaecology Exercise and Mindfulness Study (GEMS).**

**Does mindfulness or mindfulness with exercise help manage cancer-related fatigue for women with a previous diagnoses of gynaecology cancer?**

You have now finished taking part in the mindfulness/ mindfulness and exercise programme. We would like to thank you for your participation to date. We would now also like to inform you about a group discussion that will take place to evaluate this programme. The aim of the discussion is to hear your thoughts about taking part and find out how the programme could be made better for other women with gynaecology cancer in the future, all thoughts and ideas are welcomed. This discussion will last approximately 1 -1½ hours and will take place on line via MS Teams. Guidance and help using MS Teams will be provided by the research team prior to the group discussion taking place. The session will include other women who have taken part in the study and be recorded for analysis. All information will be treated in the strictest of confidence. You will find further information about this group in the attached information sheet.

We hope to schedule the sessions for the near future, however no dates have been finalised. You will be contacted by our project manager within the following week, to find out if you are interested in taking part and to answer any questions that you may have. You are under no obligation to take part.

Thank you for taking the time to consider this request.

Kind Regards

Dr Jackie Gracey

Lecturer (Physiotherapy)

Telephone: xxxx

Appendix 5

This study has been approved by the Ulster University Ethics Committee

Qualtrics link to consent:

<https://ulsterhealth.eu.qualtrics.com/jfe/form/SV_5ckh2k14byHxMWy>

**Consent Form**:

Title of Project: GEMS (Gynaecology Exercise and Mindfulness Study): A feasibility trial evaluating the effectiveness of mindfulness compared to mindfulness and exercise on fatigue in women with gynaecology cancer.

Name of researcher: Kairen McCloy

Chief Investigator: Dr Jackie Gracey

Please indicate your consent to each point by typing your initials in the boxes

k

1. I confirm that I have been given and have read and understood the Participant Information Sheet (version 5 January 2022) for the above study and have asked and received answers to any questions raised.
2. I understand that my participation is voluntary and that I am free to withdraw from the study at any time, without giving a reason and without my rights being affected in any way. If I withdraw from the study, I understand all information collected up to that point will be retained in the study.

1. I understand that all information will be held securely and in confidence, unless I disclose information that is a risk to self or others and that all efforts will be made to ensure that I will not be identified as a participant in the study.
2. If I withdraw from the study I give permission to the research team to use the data that has already been collected
3. If I withdraw from the study, I give permission to the research team to contact me on one occasion to find out why I no longer want to continue
4. I agree to take part in the study.

Name of participant (print name)_______________________________________________________

Signature of participant ________________________________________________________

Date ________________________________________________________

Name of researcher ­­­­________________________________________________________

Signature of researcher ________________________________________________________

Date ________________________________________________________

Please email a photograph or scan of the signed consent form to the researcher at McCloy-K2@ulster.ac.uk

If you would like to receive a summary of the findings, please provide your e-mail:

­­­­_________________________________________________________________________________________

**Appendix 12**

**This study has been approved by the Ulster University Ethics Committee**

**Qualtrics link:**

<https://ulsterhealth.eu.qualtrics.com/jfe/form/SV_6n7jGWDOydJjcQS>

**Consent Form**

Title of Project: GEMS (Gynaecology Exercise and Mindfulness Study): Mindfulness Focus Group Study

Name of researcher: Kairen McCloy

Chief Investigator: Dr Jackie Gracey

Please indicate your consent to each point by typing your initials in the boxes

k

1. I confirm that I have been given and have read and understood the Participant Information Sheet (Version 5 January 2022)for the above study and have asked and received answers to any questions raised.

1. I understand that my participation is voluntary and that I am free to withdraw from the study at any time, without giving a reason and without my rights being affected in any way. If I withdraw from the study, I understand all information collected up to that point will be retained in the study.
2. I understand that anonymous direct quotations may be used in subsequent publications, however, no one will be able to identify me through the information presented.
3. I understand that the session of the focus group will be audio and video recorded and that quotations from this recording may be used.
4. I understand that all information will be held securely and in confidence, unless I disclose information that is a risk to self or others and that all efforts will be made to ensure that I will not be identified as a participant in the study.
5. I agree to take part in the study.

Name of participant (print name)_______________________________________________________

Signature of participant ________________________________________________________

Date ________________________________________________________

Name of researcher ­­­­________________________________________________________

Signature of researcher ________________________________________________________

Date ________________________________________________________

Please email a photograph or scan of the signed consent form to the researcher at McCloy-K2@ulster.ac.uk

If you would like to receive a summary of the findings, please provide your e-mail:

­­­­__________________________________________________________________________________________

**This study has been approved by the Ulster University Ethics Committee**

<https://ulsterhealth.eu.qualtrics.com/jfe/form/SV_1HcWPWfjhYtgFVk>

**Consent Form**

Title of Project: GEMS (Gynaecology Exercise and Mindfulness Study): Mindfulness & exercise Focus

Group Study

Name of researcher: Kairen McCloy

Chief Investigator: Dr Jackie Gracey

Please indicate your consent to each point by typing your initials in the boxes

k

1. I confirm that I have been given and have read and understood the Participant Information Sheet (Version 5 January 2022) for the above study and have asked and received answers to any questions raised.
2. I understand that my participation is voluntary and that I am free to withdraw from the study at any time, without giving a reason and without my rights being affected in any way. If I withdraw from the study, I understand all information collected up to that point will be retained in the study.
3. I understand that anonymous direct quotations may be used in subsequent publications, however, no one will be able to identify me through the information presented.
4. I understand that the session of the focus group will be audio and video recorded and that quotations from this recording may be used.
5. I understand that all information will be held securely and in confidence, unless I disclose information that is a risk to self or others and that all efforts will be made to ensure that I will not be identified as a participant in the study.
6. I agree to take part in the study.

Name of participant (print name)_______________________________________________________

Signature of participant ________________________________________________________

Date ________________________________________________________

Name of researcher ­­­­ ________________________________________________________

Signature of researcher ________________________________________________________

Date ________________________________________________________

Please email a photograph or scan of the signed consent form to the researcher at McCloy-K2@ulster.ac.uk

If you would like to receive a summary of the findings, please provide your e-mail:

­­­­__________________________________________________________________________________________

Appendix 3

Screening for potential participants interested in the Gynaecology exercise and mindfulness study (GEMS).

1. Are you over 18 years old? Yes No
2. Have you been diagnosed with a gynaecology cancer within the last 5 years? Yes No
3. On a number score with 0 being no fatigue and 10 being very fatigued please rate where your fatigue is currently:

0—1—2—3—4—5—6—7—8—9—10

No fatigue Moderate fatigue Severe fatigue

1. Currently how much exercise are you doing?

Less than 20 mins once per week Two times per week for 30 mins

Less than 20 mins 3 times per week

1. Are you able to walk independently without the use of a walking aid? Yes No
2. Are you currently practicing mindfulness or have completed a mindfulness programme previously? Yes No
3. Do you have any of the following medical conditions?

Unstable cardiovascular disease Yes No

Unstable Hypertension Yes No

Diabetes Yes No

Epilepsy Yes No

Arthritis Yes No

Fibromyalgia Yes No

Chronic fatigue syndrome Yes No

Multiple Sclerosis (MS) Yes No

Myalgic Encephalopathy (ME) Yes No

Lupus Yes No

1. Do you have access to a smartphone/tablet/computer and are you able to use these and/or have support to the access internet. Yes No

**Appendix 6**

**Demographics Questionnaire**

**Qualtrics link to survey:**

[**https://ulsterhealth.eu.qualtrics.com/jfe/form/SV_d0ekVA37YTIuE4e**](https://ulsterhealth.eu.qualtrics.com/jfe/form/SV_d0ekVA37YTIuE4e)

**Demographics form for Gynaecology Exercise and Mindfulness Study (GEMS) feasibility study** **evaluating the effectiveness of mindfulness or mindfulness and exercise on fatigue in women with gynaecology cancer.**

1. Gender:
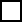
 Female
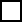
 Male
2. Date of Birth: / / .

d d m m m y y y y

1. Race (“X” ONLY one with which you MOST CLOSELY identify):

- White- English/Welsh/Scottish/Northern Irish/ British


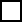
 White-Irish


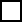
 White-Irish Traveller


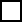
 Any other white background


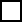
 Black/African/Caribbean/Black British-African


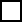
 Black/African/Caribbean/Black British-Caribbean


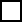
 Any other Black/African or Caribbean background


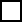
 Asian or Asian-British Indian


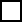
 Asian or Asian-British Pakistani


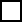
 Asian or Asian British- Bangladeshi

Asian or Asian British-Chinese


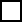


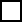
 Any other Asian background


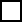
 Mixed or multiple ethnic backgrounds-White and Black Caribbean

Mixed or multiple ethnic backgrounds-White and Black African


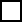


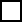
 Mixed or multiple ethnic backgrounds-White and Asian


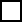
 Any other mixed or multiple ethnic backgrounds


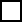
 Arab

Other ethnic background, please specify below


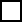


1. Marital status (“X” ONLY one):


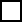
 Married


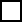
 Living with partner (not married)


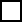
 Divorced


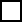
 Separated


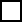
 Single


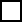
 Widowed


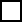
 Would rather not say


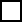
 Other

5. Type of gynaecology cancer (“X” ONLY one with which you MOST CLOSELY identify):


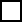
 Ovarian


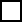
 Endometrial/Uterine


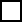
 Cervical


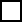
 Vaginal


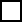
 Vulva


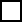
 Unknown or not reported

6. Employment status (“X” ONLY one with which you MOST CLOSELY identify):


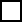
 Employed full time


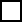
 Employed part time


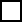
 Employed with varying hours


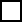
 Full-time student


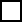
 Part time student

- Retired


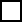
 Unemployed

- Other (please specify)

Job title:

5. Household income (“X” ONLY one with which you MOST CLOSELY identify):


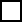
 £0-25000


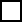
 £25001-50000


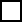
 £50001-75000


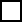
 £75001-100000


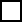
 Over £100000


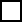
 Prefer not to say

5. Highest level of education (“X” ONLY one with which you MOST CLOSELY identify):


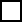
 Secondary school or equivalent


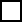
 Sixth form college or equivalent


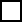
 Bachelor’s Degree


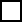
 Master’s Degree


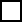
 Doctoral Degree


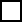
 Other (please specify)

Date Informed Consent Signed: / / .

d d m m m y y y y

Investigator Signature: / / ..

**Appendix 7**

**Qualtrics link to all the below outcome measures:** [**https://ulsterhealth.eu.qualtrics.com/jfe/form/SV_d0ekVA37YTIuE4e**](https://ulsterhealth.eu.qualtrics.com/jfe/form/SV_d0ekVA37YTIuE4e)

FACIT-F

Below is a list of statements that other people with your illness have said are important**. Please circle or mark one number per line to indicate your response as it applies to the past 7 days.**

|  | **PHYSICAL WELL-BEING** | **Not at all** | | **A little bit** | **Some-what** | **Quitea bit** | **Very much** |
| --- | --- | --- | --- | --- | --- | --- | --- |
|  |  |  |  |  |  |  |  |
| GP1 | I have a lack of energy | 0 | | 1 | 2 | 3 | 4 |
| GP2 | I have nausea | 0 | | 1 | 2 | 3 | 4 |
| GP3 | Because of my physical condition, I have trouble meeting the needs of my family | 0 | | 1 | 2 | 3 | 4 |
| GP4 | I have pain | 0 | | 1 | 2 | 3 | 4 |
| GP5 | I am bothered by side effects of treatment | 0 | | 1 | 2 | 3 | 4 |
| GP6 | I feel ill | 0 | | 1 | 2 | 3 | 4 |
| GP7 | I am forced to spend time in bed | 0 | | 1 | 2 | 3 | 4 |
|  | | | | | | | |
|  | **SOCIAL/FAMILY WELL-BEING** | **Not at all** | | **A little bit** | **Some-what** | **Quitea bit** | **Very much** |
|  |  |  |  |  |  |  |  |
| GS1 | I feel close to my friends | 0 | | 1 | 2 | 3 | 4 |
| GS2 | I get emotional support from my family | 0 | | 1 | 2 | 3 | 4 |
| GS3 | I get support from my friends | 0 | | 1 | 2 | 3 | 4 |
| GS4 | My family has accepted my illness | 0 | | 1 | 2 | 3 | 4 |
| GS5 | I am satisfied with family communication about my illness | 0 | | 1 | 2 | 3 | 4 |
| GS6 | I feel close to my partner (or the person who is my main support) | 0 | | 1 | 2 | 3 | 4 |
| Q1 | *Regardless of your current level of sexual activity, please answer the following question. If you prefer not to answer it, please mark this box and go to the next section.* | |  |  |  |  |  |
| GS7 | I am satisfied with my sex life | | 0 | 1 | 2 | 3 | 4 |

**Please circle or mark one number per line to indicate your response as it applies to the past 7 days.**

|  | **EMOTIONAL WELL-BEING** | **Not at all** | | **A little bit** | **Some-what** | **Quitea bit** | **Very much** |
| --- | --- | --- | --- | --- | --- | --- | --- |
|  |  |  |  |  |  |  |  |
| GE1 | I feel sad | | 0 | 1 | 2 | 3 | 4 |
| GE2 | I am satisfied with how I am coping with my illness | | 0 | 1 | 2 | 3 | 4 |
| GE3 | I am losing hope in the fight against my illness | | 0 | 1 | 2 | 3 | 4 |
| GE4 | I feel nervous | | 0 | 1 | 2 | 3 | 4 |
| GE5 | I worry about dying | | 0 | 1 | 2 | 3 | 4 |
| GE6 | I worry that my condition will get worse | | 0 | 1 | 2 | 3 | 4 |

|  | **FUNCTIONAL WELL-BEING** | **Not at all** | | **A little bit** | **Some-what** | **Quitea bit** | **Very much** |
| --- | --- | --- | --- | --- | --- | --- | --- |
|  |  |  |  |  |  |  |  |
| GF1 | I am able to work (include work at home) | | 0 | 1 | 2 | 3 | 4 |
| GF2 | My work (include work at home) is fulfilling | | 0 | 1 | 2 | 3 | 4 |
| GF3 | I am able to enjoy life | | 0 | 1 | 2 | 3 | 4 |
| GF4 | I have accepted my illness | | 0 | 1 | 2 | 3 | 4 |
| GF5 | I am sleeping well | | 0 | 1 | 2 | 3 | 4 |
| GF6 | I am enjoying the things I usually do for fun | | 0 | 1 | 2 | 3 | 4 |
| GF7 | I am content with the quality of my life right now | | 0 | 1 | 2 | 3 | 4 |

**Please circle or mark one number per line to indicate your response as it applies to the past 7 days.**

|  | **ADDITIONAL CONCERNS** | **Not at all** | | **A little bit** | **Some-what** | **Quite**  **a bit** | **Very much** |
| --- | --- | --- | --- | --- | --- | --- | --- |
|  |  |  |  |  |  |  |  |
| HI7 | I feel fatigued | | 0 | 1 | 2 | 3 | 4 |
| HI12 | I feel weak all over | | 0 | 1 | 2 | 3 | 4 |
| An1 | I feel listless (“washed out”) | | 0 | 1 | 2 | 3 | 4 |
| An2 | I feel tired | | 0 | 1 | 2 | 3 | 4 |
| An3 | I have trouble starting things because I am tired | | 0 | 1 | 2 | 3 | 4 |
| An4 | I have trouble finishing things because I am tired | | 0 | 1 | 2 | 3 | 4 |
| An5 | I have energy | | 0 | 1 | 2 | 3 | 4 |
| An7 | I am able to do my usual activities | | 0 | 1 | 2 | 3 | 4 |
| An8 | I need to sleep during the day | | 0 | 1 | 2 | 3 | 4 |
| An12 | I am too tired to eat | | 0 | 1 | 2 | 3 | 4 |
| An14 | I need help doing my usual activities | | 0 | 1 | 2 | 3 | 4 |
| An15 | I am frustrated by being too tired to do the things I want to do | | 0 | 1 | 2 | 3 | 4 |
| An16 | I have to limit my social activity because I am tired | | 0 | 1 | 2 | 3 | 4 |

FACT-G

Below is a list of statements that other people with your illness have said are important**. Please circle or mark one number per line to indicate your response as it applies to the past 7 days.**

|  | **PHYSICAL WELL-BEING** | **Not at all** | | **A little bit** | **Some-what** | **Quitea bit** | **Very much** |
| --- | --- | --- | --- | --- | --- | --- | --- |
|  |  |  |  |  |  |  |  |
| GP1 | I have a lack of energy | 0 | | 1 | 2 | 3 | 4 |
| GP2 | I have nausea | 0 | | 1 | 2 | 3 | 4 |
| GP3 | Because of my physical condition, I have trouble meeting the needs of my family | 0 | | 1 | 2 | 3 | 4 |
| GP4 | I have pain | 0 | | 1 | 2 | 3 | 4 |
| GP5 | I am bothered by side effects of treatment | 0 | | 1 | 2 | 3 | 4 |
| GP6 | I feel ill | 0 | | 1 | 2 | 3 | 4 |
| GP7 | I am forced to spend time in bed | 0 | | 1 | 2 | 3 | 4 |
|  | | | | | | | |
|  | **SOCIAL/FAMILY WELL-BEING** | **Not at all** | | **A little bit** | **Some-what** | **Quitea bit** | **Very much** |
|  |  |  |  |  |  |  |  |
| GS1 | I feel close to my friends | 0 | | 1 | 2 | 3 | 4 |
| GS2 | I get emotional support from my family | 0 | | 1 | 2 | 3 | 4 |
| GS3 | I get support from my friends | 0 | | 1 | 2 | 3 | 4 |
| GS4 | My family has accepted my illness | 0 | | 1 | 2 | 3 | 4 |
| GS5 | I am satisfied with family communication about my illness | 0 | | 1 | 2 | 3 | 4 |
| GS6 | I feel close to my partner (or the person who is my main support) | 0 | | 1 | 2 | 3 | 4 |
| Q1 | *Regardless of your current level of sexual activity, please answer the following question. If you prefer not to answer it, please mark this box and go to the next section.* | |  |  |  |  |  |
| GS7 | I am satisfied with my sex life | | 0 | 1 | 2 | 3 | 4 |

**Please circle or mark one number per line to indicate your response as it applies to the past 7 days.**

|  | **EMOTIONAL WELL-BEING** | **Not at all** | | **A little bit** | **Some-what** | **Quitea bit** | **Very much** |
| --- | --- | --- | --- | --- | --- | --- | --- |
|  |  |  |  |  |  |  |  |
| GE1 | I feel sad | | 0 | 1 | 2 | 3 | 4 |
| GE2 | I am satisfied with how I am coping with my illness | | 0 | 1 | 2 | 3 | 4 |
| GE3 | I am losing hope in the fight against my illness | | 0 | 1 | 2 | 3 | 4 |
| GE4 | I feel nervous | | 0 | 1 | 2 | 3 | 4 |
| GE5 | I worry about dying | | 0 | 1 | 2 | 3 | 4 |
| GE6 | I worry that my condition will get worse | | 0 | 1 | 2 | 3 | 4 |

|  | **FUNCTIONAL WELL-BEING** | **Not at all** | | **A little bit** | **Some-what** | **Quitea bit** | **Very much** |
| --- | --- | --- | --- | --- | --- | --- | --- |
|  |  |  |  |  |  |  |  |
| GF1 | I am able to work (include work at home) | | 0 | 1 | 2 | 3 | 4 |
| GF2 | My work (include work at home) is fulfilling | | 0 | 1 | 2 | 3 | 4 |
| GF3 | I am able to enjoy life | | 0 | 1 | 2 | 3 | 4 |
| GF4 | I have accepted my illness | | 0 | 1 | 2 | 3 | 4 |
| GF5 | I am sleeping well | | 0 | 1 | 2 | 3 | 4 |
| GF6 | I am enjoying the things I usually do for fun | | 0 | 1 | 2 | 3 | 4 |
| GF7 | I am content with the quality of my life right now | | 0 | 1 | 2 | 3 | 4 |

**Hospital Anxiety and Depression Scale (HADS)**

**Tick the box beside the reply that is closest to how you have been feeling in the past week.**

**Don’t take too long over you replies: your immediate is best.**

| **D** | **A** |  | **D** | **A** |  |
| --- | --- | --- | --- | --- | --- |
|  |  | **I feel tense or 'wound up':** |  |  | **I feel as if I am slowed down:** |
|  | 3 | Most of the time | 3 |  | Nearly all the time |
|  | 2 | A lot of the time | 2 |  | Very often |
|  | 1 | From time to time, occasionally | 1 |  | Sometimes |
|  | 0 | Not at all | 0 |  | Not at all |
|  |  |  |  |  |  |
|  |  | **I still enjoy the things I used to enjoy:** |  |  | **I get a sort of frightened feeling like 'butterflies' in the stomach:** |
| 0 |  | Definitely as much |  | 0 | Not at all |
| 1 |  | Not quite so much |  | 1 | Occasionally |
| 2 |  | Only a little |  | 2 | Quite Often |
| 3 |  | Hardly at all |  | 3 | Very Often |
|  |  |  |  |  |  |
|  |  | **I get a sort of frightened feeling as if**  **something awful is about to happen:** |  |  | **I have lost interest in my appearance:** |
|  | 3 | Very definitely and quite badly | 3 |  | Definitely |
|  | 2 | Yes, but not too badly | 2 |  | I don't take as much care as I should |
|  | 1 | A little, but it doesn't worry me | 1 |  | I may not take quite as much care |
|  | 0 | Not at all | 0 |  | I take just as much care as ever |
|  |  |  |  |  |  |
|  |  | **I can laugh and see the funny side of things:** |  |  | **I feel restless as I have to be on the move:** |
| 0 |  | As much as I always could |  | 3 | Very much indeed |
| 1 |  | Not quite so much now |  | 2 | Quite a lot |
| 2 |  | Definitely not so much now |  | 1 | Not very much |
| 3 |  | Not at all |  | 0 | Not at all |
|  |  | **Worrying thoughts go through my mind:** |  |  | **I look forward with enjoyment to things:** |
|  | 3 | A great deal of the time | 0 |  | As much as I ever did |
|  | 2 | A lot of the time | 1 |  | Rather less than I used to |
|  | 1 | From time to time, but not too often | 2 |  | Definitely less than I used to |
|  | 0 | Only occasionally | 3 |  | Hardly at all |
|  |  |  |  |  |  |
|  |  | **I feel cheerful:** |  |  | **I get sudden feelings of panic:** |
| 3 |  | Not at all |  | 3 | Very often indeed |
| 2 |  | Not often |  | 2 | Quite often |
| 1 |  | Sometimes |  | 1 | Not very often |
| 0 |  | Most of the time |  | 0 | Not at all |
|  |  |  |  |  |  |
|  |  | **I can sit at ease and feel relaxed:** |  |  | **I can enjoy a good book or radio or TV program:** |
|  | 0 | Definitely | 0 |  | Often |
|  | 1 | Usually | 1 |  | Sometimes |
|  | 2 | Not Often | 2 |  | Not often |
|  | 3 | Not at all | 3 |  | Very seldom |

Please check you have answered all the questions

Scoring:

Total score: Depression (D) ___________ Anxiety (A) ______________

# 0-7 = Normal 8-10 = Borderline abnormal (borderline case) 11-21 = Abnormal (case)

**Five Facet Mindfulness Questionnaire (FFMQ) Ruth A. Baer, Ph.D.**

**University of Kentucky**

------------------------------------------------------------------------------------------------------------------

**Please rate each of the following statements using the scale provided. Write the number in the blank that best describes your own opinion of what is generally true for you.**

| **1** | **2** | **3** | **4** | **5** |
| --- | --- | --- | --- | --- |
| **never or very**  **rarely true** | **rarely**  **true** | **sometimes true** | **often**  **true** | **very often or**  **always true** |

_____ 1. When I’m walking, I deliberately notice the sensations of my body moving.

_____ 2. I’m good at finding words to describe my feelings.

_____ 3. I criticize myself for having irrational or inappropriate emotions.

_____ 4. I perceive my feelings and emotions without having to react to them.

_____ 5. When I do things, my mind wanders off and I’m easily distracted.

_____ 6. When I take a shower or bath, I stay alert to the sensations of water on my body.

_____ 7. I can easily put my beliefs, opinions, and expectations into words.

_____ 8. I don’t pay attention to what I’m doing because I’m daydreaming, worrying, or otherwise distracted.

_____ 9. I watch my feelings without getting lost in them.

_____ 10. I tell myself I shouldn’t be feeling the way I’m feeling.

_____ 11. I notice how foods and drinks affect my thoughts, bodily sensations, and emotions.

_____ 12. It’s hard for me to find the words to describe what I’m thinking.

_____ 13. I am easily distracted.

_____ 14. I believe some of my thoughts are abnormal or bad and I shouldn’t think that way.

_____ 15. I pay attention to sensations, such as the wind in my hair or sun on my face.

_____ 16. I have trouble thinking of the right words to express how I feel about things _____ 17. I make judgments about whether my thoughts are good or bad.

_____ 18. I find it difficult to stay focused on what’s happening in the present.

_____ 19. When I have distressing thoughts or images, I “step back” and am aware of the thought or image without getting taken over by it.

_____ 20. I pay attention to sounds, such as clocks ticking, birds chirping, or cars passing.

_____ 21. In difficult situations, I can pause without immediately reacting.

FFMQ p. 2

| **1** | **2** | **3** | **4** | **5** |
| --- | --- | --- | --- | --- |
| **never or very**  **rarely true** | **rarely**  **true** | **sometimes true** | **often**  **true** | **very often or**  **always true** |

_____ 22. When I have a sensation in my body, it’s difficult for me to describe it because I can’t find the right words.

_____ 23. It seems I am “running on automatic” without much awareness of what I’m doing.

_____24. When I have distressing thoughts or images, I feel calm soon after.

_____ 25. I tell myself that I shouldn’t be thinking the way I’m thinking.

_____ 26. I notice the smells and aromas of things.

_____ 27. Even when I’m feeling terribly upset, I can find a way to put it into words.

_____ 28. I rush through activities without being really attentive to them.

_____ 29. When I have distressing thoughts or images I am able just to notice them without reacting.

_____ 30. I think some of my emotions are bad or inappropriate and I shouldn’t feel them. _____ 31. I notice visual elements in art or nature, such as colors, shapes, textures, or patterns

of light and shadow.

_____ 32. My natural tendency is to put my experiences into words.

_____ 33. When I have distressing thoughts or images, I just notice them and let them go.

_____ 34. I do jobs or tasks automatically without being aware of what I’m doing.

_____ 35. When I have distressing thoughts or images, I judge myself as good or bad,

depending what the thought/image is about.

_____ 36. I pay attention to how my emotions affect my thoughts and behavior.

_____ 37. I can usually describe how I feel at the moment in considerable detail.

_____ 38. I find myself doing things without paying attention.

_____ 39. I disapprove of myself when I have irrational ideas.

**FFMQ Scoring instructions**

For all items marked “R” the scoring must be reversed. Change 1 to 5, 2 to 4, 4 to 2, and 5 to 1 (3 stays unchanged). Then sum the scores for each subscale.

**Observing**

1, 6, 11, 15, 20, 26, 31, 36

**Describing**

2, 7, 12R, 16R, 22R, 27, 32, 37

**Acting with awareness**

5R, 8R, 13R, 18R, 23R, 28R, 34R, 38R

**Nonjudging of inner experience**

3R, 10R, 14R, 17R, 25R, 30R, 35R, 39R

# Nonreactivity to inner experience

4, 9, 19, 21, 24, 29, 33

**INTERNATIONAL PHYSICAL ACTIVITY QUESTIONNAIRE**

**(August 2002)**

**SHORT LAST 7 DAYS SELF-ADMINISTERED FORMAT**

# FOR USE WITH YOUNG AND MIDDLE-AGED ADULTS (15-69 years)

The International Physical Activity Questionnaires (IPAQ) comprises a set of 4 questionnaires. Long (5 activity domains asked independently) and short (4 generic items) versions for use by either telephone or self-administered methods are available. The purpose of the questionnaires is to provide common instruments that can be used to obtain internationally comparable data on health–related physical activity.

***Background on IPAQ***

The development of an international measure for physical activity commenced in Geneva in 1998 and was followed by extensive reliability and validity testing undertaken across 12 countries (14 sites) during 2000. The final results suggest that these measures have acceptable measurement properties for use in many settings and in different languages, and are suitable for national population-based prevalence studies of participation in physical activity.

## *Using IPAQ*

Use of the IPAQ instruments for monitoring and research purposes is encouraged. It is recommended that no changes be made to the order or wording of the questions as this will affect the psychometric properties of the instruments.

## *Translation from English and Cultural Adaptation*

Translation from English is supported to facilitate worldwide use of IPAQ. Information on the availability of IPAQ in different languages can be obtained at [www.ipaq.ki.se](http://www.ipaq.ki.se). If a new translation is undertaken we highly recommend using the prescribed back translation methods available on the IPAQ website. If possible please consider making your translated version of IPAQ available to others by contributing it to the IPAQ website. Further details on translation and cultural adaptation can be downloaded from the website.

# *Further Developments of IPAQ*

International collaboration on IPAQ is on-going and an ***International Physical Activity Prevalence Study*** is in progress. For further information see the IPAQ website.

## *More Information*

More detailed information on the IPAQ process and the research methods used in the development of IPAQ instruments is available at [www.ipaq.ki.se](http://www.ipaq.ki.se) and Booth, M.L. (2000). *Assessment of Physical Activity: An International Perspective*. Research Quarterly for Exercise and Sport, 71 (2): s114-20. Other scientific publications and presentations on the use of IPAQ are summarized on the website.

### INTERNATIONAL PHYSICAL ACTIVITY QUESTIONNAIRE

We are interested in finding out about the kinds of physical activities that people do as part of their everyday lives. The questions will ask you about the time you spent being physically active in the **last 7 days**. Please answer each question even if you do not consider yourself to be an active person. Please think about the activities you do at work, as part of your house and yard work, to get from place to place, and in your spare time for recreation, exercise or sport.

Think about all the **vigorous** activities that you did in the **last 7 days**. **Vigorous** physical activities refer to activities that take hard physical effort and make you breathe much harder than normal. Think *only* about those physical activities that you did for at least 10 minutes at a time.

1. During the **last 7 days**, on how many days did you do **vigorous** physical activities like heavy lifting, digging, aerobics, or fast bicycling?

_____ **days per week**

No vigorous physical activities ***Skip to question 3***

1. How much time did you usually spend doing **vigorous** physical activities on one of those days?

_____ **hours per day**

_____ **minutes per day**

Don’t know/Not sure

Think about all the **moderate** activities that you did in the **last 7 days**. **Moderate** activities refer to activities that take moderate physical effort and make you breathe somewhat harder than normal. Think only about those physical activities that you did for at least 10 minutes at a time.

1. During the **last 7 days**, on how many days did you do **moderate** physical activities like carrying light loads, bicycling at a regular pace, or doubles tennis? Do not include walking.

_____ **days per week**

No moderate physical activities ***Skip to question 5***

1. How much time did you usually spend doing **moderate** physical activities on one of those days?

_____ **hours per day**

_____ **minutes per day**

Don’t know/Not sure

Think about the time you spent **walking** in the **last 7 days**. This includes at work and at home, walking to travel from place to place, and any other walking that you have done solely for recreation, sport, exercise, or leisure.

5. During the **last 7 days**, on how many days did you **walk** for at least 10 minutes at a time?

_____ **days per week**

No walking ***Skip to question 7***

1. How much time did you usually spend **walking** on one of those days?

_____ **hours per day**

_____ **minutes per day**

Don’t know/Not sure

The last question is about the time you spent **sitting** on weekdays during the **last 7 days**. Include time spent at work, at home, while doing course work and during leisure time. This may include time spent sitting at a desk, visiting friends, reading, or sitting or lying down to watch television.

1. During the **last 7 days**, how much time did you spend **sitting** on a **week day**?

_____ **hours per day**

_____ **minutes per day**

Don’t know/Not sure

**This is the end of the questionnaire, thank you for participati**

Exercise Intervention

Home-based walking and strength exercise has been shown to not only be effective for cancer-related fatigue but also feasible and acceptable (Payne *et al.* 2008; Yeo *et al.* 2012). Indeed the current research team have demonstrated the positive effects with women who have gynaecology cancer in a previous study and this work will further develop on these findings (Donnelly *et al.* 2011a; Blaney *et al.* 2013)

The following gives a brief description of the weekly content for the home-based walking and strength intervention:

The overall aim of the programme is to achieve a goal of 30 minutes of walking 3 times per week and 2-3 strength sessions, with major muscle groups per week by the end of the 8-week intervention (Donnelly *et al.* 2011b; Gokal *et al.* 2016). These goals are suggestions and this programme will use an inductive approach to goal setting which will be decided through collaboration with participants and individual goal setting. A goal setting diary will be provided to participants to aid them to set and monitor goals throughout the study intervention. This approach has been used in previous research conducted by this team in ‘Back on Track’ and ‘Exact’ trials (Gracey *et al.* 2016; Gracey, J. H. *et al.* 2021).

**Week 1:** Walk continuously for 10 minutes once per day for 2 days per week. The strengthening and toning exercise will aim for 1 set of between 8-15 repetitions for 1 to 2 days per week of either wall press or sit to standing exercise.

**Week 2:** Walk continuously for 10 minutes once per day for 2 to 3 days per week. The strengthening and toning exercise will aim for 2 sets of between 8-15 repetitions for 1 to 2 days per week of either wall press or sit to standing exercise.

**Week 3:** Walk continuously for 10 minutes once per day for 3 days per week. The strengthening and toning exercise will aim for 2 or 3 sets of between 8-15 repetitions for 2 days per week of either wall press or sit to standing exercise.

**Week 4:** Walk continuously for 10 minutes once per day for 3 days per week. An additional goal for participants who feel able would be to add another 10-minute walk on 2 of the days. The strengthening and toning exercise will aim for 1 set of between 8-15 repetitions for 1 to 2 days per week of either lateral raises or side leg raises.

**Week 5:** Walk continuously for 10 minutes once per day for 3 days per week. An additional goal for participants who feel able would be to add another 10-minute walk on 2 of the days. The strengthening and toning exercise will aim for 2 sets of between 8-15 repetitions for 1 to 2 days per week of either lateral raises or side leg raises.

**Week 6:** Walk continuously for 10 minutes twice per day for 3 days per week. An additional goal for participants who feel able would be to add another 10-minute walk on 2 of the days. The strengthening and toning exercise will aim for 2 or 3 sets of between 8-15 repetitions for 2 days per week of either lateral raises or side leg raises.

**Week 7:** Walk continuously for 10 minutes twice per day for 3 days per week. An additional goal for participants who feel able would be to add another 10-minute walk on 2 of the days Strengthening and toning exercise will aim for 2 sets of between 8-15 repetitions for 1 to 2 days per week of either arm curls or assisted knee bends.

**Week 8:** Walk continuously for 10 minutes three times per day, or if able twice per day for 15 minutes each or once per day for 30 minutes continuously for 3 days per week. Strengthening and toning exercises will aim for 2 or 3 sets of between 8-15 repetitions for 2 days per week of either arm curls or assisted knee bends.

Participants will be encouraged to increase the intensity of the exercise dependent on the reported perceived experience of exertion using the Borg scale. This scale has a maximum level of 20 with 6 meaning no effort and 20 maximal effort (Thorsen *et al.* 2005) Participants will be asked to maintain exercise intensity to a level of 13 to 15 which is comparable to slightly strenuous and strenuous. Information on this scale along with exercise information will be available on the mobile app (appendix a). This will be supplemented with either audio or video demonstrations of individual strength exercises and explanations of walking intensity (appendix a). Participants will be asked to keep exercise logs recording the time and intensity of the walk using the Borg scale along with the number of sets and repetitions of strength exercises performed (appendix a). Participants will be contacted by the researcher every week at a time agreed between participant and researcher, this weekly contact will be to assess if there are any issues and set goals for the following week. Participants will be given the contact details of the researcher if they have any questions or concerns regarding the intervention. Participants will be educated as to what side effects to expect and any that they should be reporting to the research team. The researcher, Kairen McCloy, has completed a strength and conditioning course to enable her to safely educate and discuss goals with participants. A distress protocol is in place and will be followed if required. (appendix b). Alongside this guidance on managing participants that may have bone metastasis will be taken form the Macmillan guide ‘Physical Activity for People with Metastatic Bone Disease’ (Macmillan ). A risk assessment has been performed and interventions to reduce risk have been implemented.

Mindfulness

Mindfulness will be based on the mindfulness-based stress reduction developed by Kabat-Zinn (Jon Kabat-Zinn, Thich Nhat Hanh 2013). This will involve 8 weeks of weekly web-based session and home practice of formal and informal mindfulness practice which will include body scan, sitting, walking, mountain and loving-kindness practice (Lengacher *et al.* 2012; Johns *et al.* 2015; Reich *et al.* 2017). On line, web-based or app delivered mindfulness although in its infancy for this intervention, has its advantages. It can reach a wider population, helps with barriers such as child care, work schedules and is flexible. It also has the added cost-saving not only to the health care systems but also to the participants while still able to maintain the intervention experience through this newer electronic less researched and reviewed platform (Subnis *et al.* 2020). The practitioner delivering this intervention has completed training on an accredited course run by the Irish Mindfulness Academy.

The following gives a brief description of the weekly content for the mindfulness intervention:

**Week 1:** Introduction to mindfulness. Automatic pilot and cultivating awareness. Introduction to body scan practice. Homework body scan mediation and eating one meal mindfully.

**Week 2:** The association of moods and thoughts. The barriers to practice. Introduction to breathing mindfully. Homework pleasant events diary and alternating between breathing and body scan practice.

**Week 3:** Attitudes of mindfulness such as non-judging and acceptance. Introduction to sitting practice. Homework sitting practice and a gratitude journal.

**Week 4:** Staying in the present. Being aware of mind wondering. Reactions to unpleasant experiences and coping with stress. Introduction of walking practice and STOP practice. Homework alternate between body scan, sitting and walking practice. Awareness and acceptance of negative thoughts.

**Week 5:** Allowing and letting go. Letting things be the way they are with compassion and acceptance. Introduction to the loving-kindness practice. Homework loving-kindness practice or sitting practice.

**Week 6:** Thoughts are not facts. How these are different and the impact these have on thought patterns and behaviours. Introduction to mountain practice and 3 minute breath practice. Homework continues with a practice of choice and gratitude journaling.

**Week 7:** Taking care of yourself. Looking at links between mood and activity. The triggers and behaviours that nourish and deplete us. Homework continues with the home practice of choice. Aware of triggers that nourish or deplete and affect mood.

**Week 8:** Completion of the course and adapting to suit the real world. Reviewing the material learnt over the last 7 weeks and how to adapt this into life. Homework chooses own mindful practice. Reflections and implementations of future mindfulness.

The above intervention will be delivered through a secure mobile application platform. Participants that have completed the screening and consent process and have been randomised will be sent the link to access a web portal via the e-mail they have provided at screening. The portal once logged in will give the participants instructions on how to download the mobile application. This will be an asynchronous intervention so participants may access the system in their own time and on whichever device suits them. Completion of each weeks module will be encouraged before participants move to the following week. The estimated time to complete each week's online session will be between 1-2 hours with the homework assignments varying between 10 minutes in the initial weeks to up to 25 mins for 5-6 days per week by week 8, this can be adapted to suit the individual schedule (appendix a). Logging of mindfulness practice will be encouraged. Participants will be contacted by the researcher every week at a time agreed between participant and researcher at start of the study, this weekly contact will be to assess if there are any issues and set goals for the following week. Participants will be given the contact details of the researcher if they have any questions or concerns regarding the intervention. Participants will be encouraged to report any issues to the research team. A distress protocol is in place and will be followed if required. (appendix b).

**Appendix a**

**Description of the contents and material of mobile application.**

**Week 1 material & schedule for app**

| Mindfulness app participant view | Resources |
| --- | --- |
| Home page with introduction by the research team | Video (will insert once recorded) |
| This first weeks audio recording, which is 2:23 minutes long it is an introduction to fatigue and cancer (mindfulness) that participants can listen to as often as they would like. It is a short introduction to fatigue and its impact following a diagnosis of cancer and treatment for cancer. | Script for audio:   |
| Week 1 introduction part 1 what is mindfulness.  A short audio 4:35 minutes long that introduces participants to what mindfulness is and introduction to practices. Will look at the benefits of mindfulness and the terms auto pilot and what that means. | Script for audio:   |
| Week 1 introduction part 2 how to practice mindfulness. This short audio 4:34 minutes describes the practice of mindfulness what to do while practicing. The barriers participants may face and how to overcome these. | Script please see above in Week 1 Introduction part 1. |
| This is a short formal practice, 4:18 minutes long that focuses on the body. Participants are encouraged to practice 4-5 days per week. | Script for audio:   |
| This practice is similar to the above practice but is slightly longer, 14:42 minutes. Focuses on each body part a little longer before moving on to the next. Participants are encouraged to practice 4-5 days per week, either of the mindfulness practices can be used by participants. | Script for audio:   |
| Log of mindfulness practice  This will be a log for participants to record their practice for the week. Will be drop down menus for easy recording. |  |

| Exercise app participant view | Resources |
| --- | --- |
| Home page with introduction by the research team short video 2:13 minutes with Dr Gracey introducing the study and some background. What the study will entail and welcoming participants to the study. |  |
| Introduction to fatigue and cancer (exercise) 1 short audio 4:32 and 1 video 4:01 minutes long. Includes what may causes fatigue and the effects it has. How exercise and mindfulness may help. What will be involved in the study. | Script for audio:   |
| Getting started  These initial audios and videos explain what things to be thinking about when stating the programme. Explains equipment needed, places to walk, chairs and shoes that may make completing the program easier. The pros and cons and barriers that participants may experience and how to overcome some of these. How to walk safely and what to do if injured. There are 2 short videos and 1 audio getting started: 4:07 minutes is an audio, choosing a chair 2:46 minutes and choosing shoes 2:13 minutes are both videos. | Script for audio:   |
| Warm up  This document demonstrates the exercise to perform in a warm before attempting any strength and toning exercise. Why it is important to do these and how avoid injury. The warm up should take approximately 5 minutes. | Word doc   |
| Strengthening and toning  This 3:41 minutes audio describes what strength and toning exercise are and what muscle groups they will work. Describes the safety around performing these. What repetitions and sets are. The adaptions that can be made and how to and when to progress these on. | Word doc for strengthening & toning   |
| Walking programme  This section describes the walking programme the safety around this. The aim and targets for the 8 weeks of the programme for walking. The pace and the need for rest and recovery. Borg scale (word doc) is described and how participants will be using it during the study. The audio is 2:24 minutes long and describes the walking part of the program. The video is 2:04 minutes long and also gives a over view of the walking component. | Script for audio: The Borg scale:   |
| Week 1 Goals for walking and strength examples of goals that participants can use or adapt to set goals for this week. | Word doc ? display this in area of app where participants will be able to set their own goals, possibly using a drop down menu so can pick how many walks, steps and times for the week same for the strength so how many reps/sets of what exercise and how many times for that week.   |
| Week 1-3 Seated to standing exercise. This video is 1:20 minutes long and demonstrates this exercise how to progress it, if it is too easy and how to regress it if too hard. |  |
| Week 1-3 wall press  This video is 1:34 minutes long and demonstrates this exercise how to progress it, if it is too easy and how to regress it if too hard. | word doc for demo of wall press   |
| Log of exercise and walking activity. | Manual log with drop down of walking and minutes. For strength a drop down of type of exercise and sets and reps. Borg scale so participants can slide across at how much effort the exercise activity exerted. |

**Week 2 material & schedule for app**

| Mindfulness app participant view | Resources |
| --- | --- |
| This 5:03 minutes long audio describes how thoughts affect us and will focus a little more on thoughts and how they affect emotion and behaviour. Types of barriers faced when practicing mindfulness and how to overcome. | Script for audio:   |
| This weeks practice is breathe practice this short 4:09 minutes long practice is the guided formal practice. Participants encouraged to practice 4-5 days this week. | Breathing practice script:   |
| This is the longer breathe practice 13:48 minutes long. This allows participants to make the choice of practice that would most suit them. Participants encouraged to practice 4-5 days this week. | Word doc script for audio:   |
| Log of mindfulness practice. | Manual log of type and whether short/long practice. |

| Exercise app participant view | Resources |
| --- | --- |
| Week 2 Goals for walking and strength, examples of goals that participants can use or adapt to set goals for this week. | Word doc ? display this in app before starting exercise so participants can see and can refer back to.   |
| Week 1-3 Seated to standing exercise demonstration same as previous week. | Please see last week for resources. |
| Week 1-3 wall press demonstration same as previous week. | Please see last week for resources. |
| Log of exercise and walking activity included is a sample log where participants will log their activity for that day/week. | Manual log with drop down of walking and minutes. For strength a drop down of type of exercise and sets and reps. Borg scale so participants can slide across at how much effort the exercise activity exerted. |

**Week 3 material and schedule for app**

| Mindfulness app participant view | Resources |
| --- | --- |
| This audio which is 9:43 minutes long describes the intentions when practicing mindfulness. It then describes attitudes and how both intentions and attitudes effect mindfulness practice and how important they are for mindfulness practice. | Script for audio:   |
| This week formal practice is the sitting practice, the shorter version is 9:44 in length. Participants encouraged to practice this or any of the other practices introduced so far for 4-5 days this week. | Script fpr audio:   |
| This is the longer sitting practice at 17:42 minutes, again allowing participants to choose which practice would suit them and can be used alternatively with any of the other practices introduced so far. Participants are encouraged to practice for 4-5 days this week. | Script for audio:   |
| Log of mindfulness practice. | Manual log of type and whether short/long practice. |
| Exercise app participant view | Resources |
| Week 3 Goals for walking and strength an example of goals that participants can use or adapt to set goals for this week. | Word doc ? display this in app before starting exercise so participants can see and can refer back to.   |
| Week 1-3 Seated to standing exercise demonstration same as previous weeks. | Please see previous week resources. |
| Week 1-3 wall press demonstration same as previous weeks. | Please see previous week resources. |
| Log of exercise and walking activity included is a sample log where participants will log their activity for that day/week. | Manual log with drop down of walking and minutes. For strength a drop down of type of exercise and sets and reps. Borg scale so participants can slide across at how much effort the exercise activity exerted. |

**Week 4 material and schedule for app**

| Mindfulness app participant view | Resources |
| --- | --- |
| This week’s audio is 7:53 minutes long and describes staying in the present moment and reactions to unpleasant feelings and coping with stress. It describes the stress response and some of the physical and emotional feelings may experience with stress and learning to recognise them so by becoming more aware of them can learn to deal with them sooner. | Script for audio:   |
| This weeks’ formal practice is a walking practice, it guides participants through a walk and how mindfulness is present during this. It is 10:26 minutes long. Participants are encouraged to practice 4-5 days per week but can use any of the practices introduced so far. | Script for audio:   |
| Log of mindfulness practice | Manual log of type and whether short/long practice. |
| Exercise app participant view | Resources |
| Week 4 Goals for walking and strength examples of goals that participants can use or adapt to set goals for this week. | Word doc ? display this in app before starting exercise so participants can see and can refer back to.   |
| Week 4-6 lateral raises exercise demonstration of this week’s exercise. The video is 1:06 minutes long and demonstrates this exercise how to progress it, if it is too easy and how to regress it if too hard. |  |
| Week 4-6 leg raises demonstration of this week’s exercise. The video is 1:23 minutes long and demonstrates this exercise how to progress it, if it is too easy and how to regress it if too hard. |  |
| Log of exercise and walking activity included is a sample log where participants will log their activity for that day/week. | Manual log with drop down of walking and minutes. For strength a drop down of type of exercise and sets and reps. Borg scale so participants can slide across at how much effort the exercise activity exerted. |

**Week 5 material and schedule for app**

| Mindfulness app participant view | Resources |
| --- | --- |
| This week’s audio is 6:22 minutes long and introduces another attitude, the attitude of kindness and compassion along with letting go or sometimes this can be called non-attachment. This can help participants to understand and acknowledge feelings toward themselves and others instilling a sense of calmness that can help deal with and regulate other emotions. | Script for audio:   |
| This week’s formal practice is a loving kindness practice. It is 14:26 minutes long. Participants are encouraged to practice 4-5 days this week but can practice any of the practices introduced so far. | Script for audio:   |
| Log of mindfulness practice. | Manual log of type and whether short/long practice. |
| Exercise app participant view | Resources |
| Week 5 Goals for walking and strength examples of goals that participants can use or adapt to set goals for this week. | Word doc ? display this in app before starting exercise so participants can see and can refer back to.   |
| Week 4-6 lateral raises exercise demonstration same as last weeks. | Please see last week’s resources. |
| Week 4-6 leg raises demonstration same as last weeks. | Please see last week’s resources. |
| Log of exercise and walking activity included is a sample log where participants will log their activity for that day/week. | Manual log with drop down of walking and minutes. For strength a drop down of type of exercise and sets and reps. Borg scale so participants can slide across at how much effort the exercise activity exerted. |

**Week 6 material and schedule for app**

| Mindfulness app participant view | Resources |
| --- | --- |
| This audio is 7:31 minutes long, it describes thoughts and how they are not facts. It looks at how thoughts effects our behaviour and emotions more in depth and ties in week twos information also how changing this will not only change our brains but also our behaviour and how we interpret and react to things. | Script for audio:   |
| This weeks’ formal practice include a short 3 minute practice that can be used by participants when feeling overwhelmed. The mountain practice is also introduced and is 11:48 minutes long practice. It uses imagery in this practice which some participants may find easier to relate to. Participants are encouraged to practice 4-5 days this week but can use any of the practices along with the new ones introduced this week.  To do script for 3 minute breath | Script for audio:   |
| Log of mindfulness practice. | Manual log of type and whether short/long practice. |
| Exercise app participant view | Resources |
| Week 6 Goals for walking and strength examples of goals that participants can use or adapt to set goals for this week. | Word doc ? display this in app before starting exercise so participants can see and can refer back to.   |
| Week 4-6 lateral raises exercise demonstrations same as last week. | Please see last week’s resources. |
| Week 4-6 leg raises demonstration same as last week. | Please see last week’s resources. |
| Log of exercise and walking activity included is a sample log where participants will log their activity for that day/week. | Manual log with drop down of walking and minutes. For strength a drop down of type of exercise and sets and reps. Borg scale so participants can slide across at how much effort the exercise activity exerted.   |

**Week 7 material and schedule for app**

| Mindfulness app participant view | Resources |
| --- | --- |
| This weeks’ audio is 7:08 minutes long and describes more closely how mood can effect activity. Explores triggers that may lead to behaviours such as negative thinking and how mindfulness can help manage these. Also examines activities that can deplete and nourish and further help manage mood and behaviour. | Script for audio:   |
| This weeks formal practice is any of the practices already described, participants may choose the practices for this week. Participants will be encouraged to try to practice 4-5 days this week. | Please see previous audio and scripts already described. |
| Log of mindfulness practice. | Manual log of type and whether short/long practice. |
| Exercise app participant view | Resources |
| Week 7 Goals for walking and strength examples of goals that participants can use or adapt to set goals for this week. | Word doc ? display this in app before starting exercise so participants can see and can refer back to.   |
| Week 7-8 arm curls demonstration of this week’s exercise. The video is 1:12 minutes long and demonstrates this exercise how to progress it if it is too easy and how to regress it if too hard. |  |
| Week 7-8 assisted knee bends demonstration of this week’s exercise. The video is 1:09 minutes long and demonstrates this exercise how to progress it, if it is too easy and how to regress it if too hard. |  |
| Log of exercise and walking activity included is a sample log where participants will log their activity for that day/week. | Manual log with drop down of walking and minutes. For strength a drop down of type of exercise and sets and reps. Borg scale so participants can slide across at how much effort the exercise activity exerted. |

**Week 8 material and schedule for app**

| Mindfulness app participant view | Resources |
| --- | --- |
| This week an overview of the previous weeks will be described. Participants will be encouraged to adapt mindfulness practice into daily practice. | Audio and word doc. |
| This week’s formal practice is any of the practices already described participants may choose which they wish to do. Participants will be encouraged to try and practice for 4-5 days this week. | Please see previous audio and scripts already described |
| Log of mindfulness practice. | Manual log of type and whether short/long practice. |
| Exercise app participant view | Resources |
| Week 8 Goals for walking and strength examples of goals that participants can use or adapt to set goals for this week. | Word doc ? display this in app before starting exercise so participants can see and can refer back to.   |
| Week 7-8 arm curls same as last week’s demonstration. | Please see last week’s resources. |
| Week 7-8 assisted knee beds same as last week’s demonstration. | Please see last week’s resources. |
| Log of exercise and walking activity included is a sample log where participants will log their activity for that day/week. | Manual log with drop down of walking and minutes. For strength a drop down of type of exercise and sets and reps. Borg scale so participants can slide across at how much effort the exercise activity exerted. |
|  |  |

**Appendix b**

Distress protocol for physical events for participants in intervention study.

If not resolved or increased researcher will seek advice from physiotherapist (JG) or (JM)

If symptoms resolved and feel willing can recommence study.

If assessment demonstrates effects researcher will speak to physiotherapist in research team (JG) or JM) for advice

If symptoms not resolved or increased to refer for further medical advice.

If symptoms resolved and feel willing can recommence study.

If effects do warrant further medical advice participants will be referred eg to GP or ED if needed.

If effects do not warrant further medical advice then researcher advice appropriate measures.

Following physiotherapist advice researcher will advise participants to either of the following :

If assessment demonstrates that effects were mild advice rest and review at agreed timepoint.

Researcher (qualified nurse) performs an assessment either via telephone or web link (zoom).

Participant calls researcher experiencing a physical event such as muscle soreness, new or increasing pain, increase in fatigue.

Distress protocol for emotional distress and stress for participants in intervention study.

Purpose

This distress protocol has been developed for participants that may become upset whilst taking part in the mindfulness intervention of this research project. For some, participating in mindfulness may result in unpleasant feelings such as anxiety (Van Dam *et al.* 2018). In the event that participants become distressed or upset, the following actions will be taken.

Support

Participants will be contacted weekly by researcher as part of the inductive approach of this project. This weekly contact will give the participant and researcher opportunity to establish if any distressing issues have risen, if issues arise outside of this weekly contact participants will also be encouraged to contact the research team or organisations that participants will be made aware of at study entry.

This assessment will be made by the researcher (qualified nurse with advance communication training). If participants feel able to continue reassurance will be given, if participants unable to continue than signposting to appropriate organisations will be made and documentation of when the distress protocol is used will be kept.

Follow up with participants will be made. The research team will be informed and documentation will be completed.

Flow diagram :Adapted from: Draucker CB, Martsolf DS & Poole C (2009) Developing distress protocols for research on sensitive topics. Archives of Psychiatric Nursing 23 (5) 343-350

Participant are called or call the researcher experiencing emotional distress or stress.

Researcher (qualified nurse) performs assessment either via telephone or web link (zoom).

Inform the research team of event and complete any appropriate documentation (Ethics)

If participant feels able to continue give reassurance and allow to continue within study.

If participant feels unable to continue offer referral to either GP for support or to another organisation such as Macmillan, Action Cancer or Cancer Focus for appropriate support.

Follow up call with participants.

Distress protocol for researcher during intervention study.

There is a potential of physical and psychological impact on the researcher during the period of recruitment into the intervention study.

A mechanism for debriefing with the PI and research team should be established to provide support during the recruitment phase of the study.

Encourage the researcher to seek counselling and other support (available through University or GP) in addition to debriefing and supervision should she experience increased distress as a result of engagement in research activities.

Adapted from: Draucker CB, Martsolf DS & Poole C (2009) Developing distress protocols for research on sensitive topics. Archives of Psychiatric Nursing 23 (5) 343-350

References.

Blaney, J.M., Lowe-Strong, A., Rankin-Watt, J., Campbell, A. and Gracey, J.H. (2013) Cancer survivors' exercise barriers, facilitators and preferences in the context of fatigue, quality of life and physical activity participation: a questionnaire-survey. *Psycho-Oncology,* 22(1), 186-194.

Donnelly, C.M., Blaney, J.M., Lowe-Strong, A., Rankin, J.P., Campbell, A., McCrum-Gardner, E. and Gracey, J.H. (2011a) A randomised controlled trial testing the feasibility and efficacy of a physical activity behavioural change intervention in managing fatigue with gynaecological cancer survivors. *Gynecologic Oncology,* 122(3), 618-624.

Donnelly, C.M., Blaney, J.M., Lowe-Strong, A., Rankin, J.P., Campbell, A., McCrum-Gardner, E. and Gracey, J.H. (2011b) A randomised controlled trial testing the feasibility and efficacy of a physical activity behavioural change intervention in managing fatigue with gynaecological cancer survivors. *Gynecologic Oncology,* 122(3), 618-624.

Gokal, K., Wallis, D., Ahmed, S., Boiangiu, I., Kancherla, K. and Munir, F. (2016) Effects of a self-managed home-based walking intervention on psychosocial health outcomes for breast cancer patients receiving chemotherapy: a randomised controlled trial. *Supportive Care in Cancer,* 24(3), 1139-1166.

Gracey, J.H., McDermott, L., Murphy, M., Rankin, J. and McNeilly, A.M. (2021) The feasibility and acceptability of a home based exercise intervention for colorectal cancer survivors: ‘EXACT’ – EXercise And Colorectal Cancer Trial. *Physical Therapy and Rehabilitation,* 8(1), 3. Available at: <http://www.hoajonline.com/phystherrehabil/2055-2386/8/3>

Gracey, Payne, C. ( 1 ), Watson, M. ( 2 ), Rankin, J. ( 3 ) and Dunwoody, L. ( 4 ). (2016) Translation research: 'Back on Track', a multiprofessional rehabilitation service for cancer-related fatigue. *BMJ Supportive and Palliative Care,* 6(1), 94-96.

Johns, S.A., Brown, L.F., Monahan, P.O., Tong, Y., Kroenke, K. and Beck-Coon, K. (2015) Randomized controlled pilot study of mindfulness-based stress reduction for persistently fatigued cancer survivors. *Psycho-Oncology,* 24(8), 885-893.

Jon Kabat-Zinn, Thich Nhat Hanh. (2013) *Full Catastrophe Living: Using the Wisdom of your body and mind to face stress, pain and illness.* Revised Edition ed. New York: Bantam.

Lengacher, Reich, Post-White, Moscoso, Shelton, Barta, Le and Budhrani. (2012) Mindfulness based stress reduction in post-treatment breast cancer patients: an examination of symptoms and symptom clusters. *Journal of Behavioral Medicine,* 35(1), 86-94.

Macmillan. *Physical Activity for People with Metastatic Bone Disease.* [Accessed 24/09/21].

Payne, J.K., Held, J., Thorpe, J. and Shaw, H. (2008) Effect of exercise on biomarkers, fatigue, sleep disturbances, and depressive symptoms in older women with breast cancer receiving hormonal therapy. *Oncology Nursing Forum,* 35(4), 635-642.

Reich, R.R., Lengacher, C.A., Alinat, C.B., Kip, K.E., Paterson, C., Ramesar, S., Han, H.S., Ismail-Khan, R., Johnson-Mallard, V., Moscoso, M., Budhrani-Shani, P., Shivers, S., Cox, C.E., Goodman, M. and Park, J. (2017) Mindfulness-Based Stress Reduction in Post-treatment Breast Cancer Patients: Immediate and Sustained Effects Across Multiple Symptom Clusters. *Journal of Pain & Symptom Management,* 53(1), 85-95.

Subnis, U.B., Farb, N.A.S., Piedalue, K.L., Speca, M., Lupichuk, S., Tang, P.A., Faris, P., Thoburn, M., Saab, B.J. and Carlson, L.E. (2020) A Smartphone App–Based Mindfulness Intervention for Cancer Survivors: Protocol for a Randomized Controlled Trial. *JMIR Research Protocols,* 9(5), e15178.

Thorsen, L., Skovlund, E., Strømme, S.B., Hornslien, K., Dahl, A.A. and Fosså, S.D. (2005) Effectiveness of physical activity on cardiorespiratory fitness and health-related quality of life in young and middle-aged cancer patients shortly after chemotherapy. *Journal of Clinical Oncology,* 23(10), 2378-2388.

Van Dam, N.,T., van Vugt, M.,K., Vago, D.R., Schmalzl, L., Saron, C.D., Olendzki, A., Meissner, T., Lazar, S.W., Kerr, C.E., Gorchov, J., Fox, K.C.R., Field, B.A., Britton, W.B., Brefczynski-Lewis, J. and Meyer, D.E. (2018) Mind the Hype: A Critical Evaluation and Prescriptive Agenda for Research on Mindfulness and Meditation. *Perspectives on Psychological Science : A Journal of the Association for Psychological Science,* 13(1), 36-61.

Yeo, T.P., Burrell, S.A., Sauter, P.K., Kennedy, E.P., Lavu, H., Leiby, B.E. and Yeo, C.J. (2012) A Progressive Postresection Walking Program Significantly Improves Fatigue and Health-Related Quality of Life in Pancreas and Periampullary Cancer Patients. *Journal of the American College of Surgeons,* 214(4), 463-475.

stylefix

Appendix 9

COM-B and BCW

As outlined earlier the COM-B model along with the BCW will be used to effectively implement both interventions of exercise and mindfulness. The Behaviour Change Taxonomy v1 (BCT) will be used to further refine the interventions used, all of which will relate to the constructs of the COM-B model (Michie, S. ( 1 ) *et al.* 2011; Michie, Susan *et al.* 2011).

Within the capability construct of the model the intervention functions that will be employed for both the interventions in this study will be training, enablement and education (Michie, S. ( 1 ) *et al.* 2011). For the physical capability participants will be given training through the online platform on how to perform both interventions. Psychological capabilities will be around education and the health consequences of performing the interventions, which will be provided in written and audio form on the mobile application. The BCT that these interventions relate to include information about health consequences, emotional consequences and information on how to perform and demonstration of an intervention. Together both the capabilities and BCT will enable the participants to perform the interventions (West *et al.* 2019).

Physical and Social opportunity would use the interventions environmental restructuring and enablement. Physical opportunity would include identifying the barriers that may be present that would inhibit the performing of the interventions and then developing strategies to overcome this (West *et al.* 2019) . This would be problem solving, goal setting and action planning within the BCT taxonomy . For social opportunity this could involve both the practical or emotional aspects of the BCT (Michie *et al.* 2011). Participants could through the problem solving identify that child care is a barrier and family support may help to overcome this barrier and therefore enable them to perform the intervention successfully.

Motivation construct for this studies interventions will use the intervention functions incentivisation and persuasion (West *et al.* 2019). For automatic motivation the use of persuasion that the intervention will have other positive social rewards such as alone time or time with family. This social reward can also be seen as incentivisation where by feedback is given to participants on the completion of goals. This mechanism can enhance reflective motivation and together with establishing the pros and cons of the behaviour can further enhance motivation. Other interventions such as self-monitoring through diaries and prompts and cues by text or e-mail reminding participants of how well they are progressing and also to perform the intervention can also enable motivation (Michie *et al.* 2011)

References.

Michie, S. ( 1 ), van Stralen, M. M. ( 2 ) and West, R. ( 3 ). (2011) The behaviour change wheel: A new method for characterising and designing behaviour change interventions. *Implementation Science,* 6(1)

Michie, S., Ashford, S., Sniehotta, F.F., Dombrowski, S.U., Bishop, A. and French, D.P. (2011) A refined taxonomy of behaviour change techniques to help people change their physical activity and healthy eating behaviours: The CALO-RE taxonomy. *Psychology & Health,* 26(11), 1479-1498.

West, Michie, Atkins, Chadwick and Loerncatto. (2019) *Achieving behaviour change
A guide for local government and
partners.* London: PHE publications.

stylefix

Appendix 13

Focus group semi-structured question guide.

- What were your initial feelings/reactions to joining an exercise/mindfulness study?
- What were your main reasons for wanting to take part in this study?
- Can you tell us about what you thought of taking part in an online programme?
- Can you tell us what did you like the most about the programme?
- What did you like the least about the programme?
- What were some of the benefits that you experienced while participating in the intervention?
- What were some of the barriers that you experienced while participating in the intervention?
- Can you think of any ways that the programme can be improved?
- How could you continue the practices learned during this intervention for the long term?
- Is there anything else you would like to add regarding your experience of taking part in this programme?

**Appendix 14**

**Study Timeline**

|  | **STUDY PERIOD** | | | | | | | |
| --- | --- | --- | --- | --- | --- | --- | --- | --- |
|  | **Enrolment** | **Allocation** | **Post-allocation** | | | | | **Close-out** |
| **TIMEPOINT**** | ***-t_1_*** | **0** | ***t_1_*** |  |  |  | ***t_3_*** | ***t_x_*** |
| **ENROLMENT:** |  |  |  |  |  |  |  |  |
| **Eligibility screen** | X |  |  |  |  |  |  |  |
| **Informed consent** | X |  |  |  |  |  |  |  |
| **Allocation** |  | X |  |  |  |  |  |  |
| **INTERVENTIONS:** |  |  |  |  |  |  |  |  |
| ***Mindfulness only*** |  |  |  |  |  |  |  |  |
| ***Mindfulness and Exercise.*** |  |  |  |  |  |  |  |  |
| **ASSESSMENTS:** |  |  |  |  |  |  |  |  |
| ***Demographics*** | X | X |  |  |  |  |  |  |
| ***FACIT-F**** |  | X |  |  |  |  |  | X |
| ***FACT-G***** |  | X |  |  |  |  |  | X |
| ***PSQI****** |  | X |  |  |  |  |  | X |
| ***FFM-Q†*** |  | X |  |  |  |  |  | X |
| ***HADS†**** |  | X |  |  |  |  |  | X |
| ***IPAQ-SF††*** |  | X |  |  |  |  |  | X |

* The Functional Assessment of Chronic Therapy- Fatigue (FACIT-F)

** The Functional Assessment of Cancer Therapy General (FACT-G)

*** Pittsburgh Sleep Quality Index (PSQI)

†Five Facet Mindfulness Questionnaire Short Form (FFMQ-SF)

†*Hospital Anxiety and Depression Score (HADS)

††International physical activity questionnaire short form (IPAR-Q -S

Appendix 15

Distress protocol for focus groups

Purpose

This distress protocol has been developed for those participants that may become upset during participation in focus groups. In the event that a participant becomes distressed the following action will be taken:

Privacy will be maintained at all times by the research team. If a participant becomes distressed, upset or emotional during the focus group they will be given the option of taking a break from the group. They will be accompanied by one of the facilitators to a break out room if they wish to take a break. The participant will be reminded that they can with draw from the study and their decision will be respected.

The participant will be supported by the one of the facilitator who has experience of conducting focus groups and is a qualified physiotherapist. If participants feel able to re-join the group they can re enter the focus group. If not and they feel or the facilitator feel they are unable they can terminate their participation in the group. Participant consent will be sought for onward referral if necessary to supporting organisation such as their GP or Clinical Nurse Specialist. Information for other supporting organisation will also be provided such as Macmillan Cancer Support or Cancer Focus NI.

The researcher will keep a record of when the distress protocol is used and actions taken, this will be reported to Chief Investigator.

*Distress protocol flow diag*

Participant returns to focus group and continue to observe for distress

Distress recognised

Participant given opportunity to take a break or terminate participation in focus group

Interview terminated.

Support offered. Verbal consent obtained for onward referral if appropriate

Record discussion
