# Supplementary material for: The effectiveness of mindfulness alone compared to exercise and mindfulness on fatigue in women with gynaecology cancer (GEMS): Protocol for a randomised feasibility trial
Source: PLoS One. 2023 Oct 26;18(10):e0278252. doi: 10.1371/journal.pone.0278252 (PMC10602305; doi:10.1371/journal.pone.0278252)
Supplement: S2 File — Trial registration data (DOCX) [file pone.0278252.s003.docx]

**Trial registration data:**

Primary registry and trial identifying number: NCT05561413

Date of registration in primary registry: September 20 2022

Secondary identifying numbers: CN-02467186

Source of monetary or material support: Department for the Economy Northern Ireland

Primary sponsor: Ulster University

Contact for public enquires: Dr Jackie Gracey

Contact for scientific enquires: Professor Ciara Hughes

Public Title: Gynaecology Exercise and Mindfulness Study (GEMS)

Scientific title: Randomised Controlled Feasibility Trial Evaluating the Effectiveness of Mindfulness Compared to Exercise and Mindfulness on Fatigue in Women with Gynaecology Cancer

Countries of recruitment: UK

Health condition or problems : Gynaecology cancer, cancer related fatigue

Intervention: Intervention: Mindfulness and exercise Active comparator: Mindfulness

Key inclusion and exclusion criteria: Ages eligible for study: > 18 years Inclusion: Women diagnosed with gynaecology cancer, still experiencing fatigue > 4 on NRS scale Exclusion: Currently practicing mindfulness, diagnosis of schizophrenia, bipolar, psychosis risk, existing medical condition inhibits safe participation.

Study type: Interventional. Allocation: Randomised parallel 1:1 Primary purpose: feasibility

Date of first enrolment: June 2022

Target sample size: 40

Recruitment status: Recruiting

Primary outcomes: Feasibility: eligibility, retention, attrition and adherence

Key Secondary outcomes: Fatigue, quality of life, sleep, psychological outcome: anxiety, depression, mindfulness and physical activity
