# Supplementary material for: Correction: The effectiveness of mindfulness alone compared to exercise and mindfulness on fatigue in women with gynaecology cancer (GEMS): Protocol for a randomised feasibility trial
Source: PLoS One. 2026 Jan 2;21(1):e0340148. doi: 10.1371/journal.pone.0340148 (PMC12758691; doi:10.1371/journal.pone.0340148)
Supplement: S2 File — (PDF) [file pone.0340148.s002.pdf]

PLOS One

STUDY PROTOCOL

The effectiveness of mindfulness alone compared to exercise and mindfulness on fatigue in women with gynaecology cancer (GEMS): Protocol for a randomised feasibility trial

Kairen McCloy[1☯], Ciara Hughes[1☯], Lynn Dunwoody[2☯], Joanne Marley[1☯], Ian Cleland[3☯], Federico Cruciani[3☯], Catherine Saunders[3☯], Jackie Gracey[1☯*]

1 Institute of Nursing and Health Research, Ulster University, Newtownabbey, United Kingdom, **2** Psychology Research Institute, Ulster University, Coleraine, United Kingdom, **3** School of Computing, Ulster University, Newtownabbey, United Kingdom

☯ These authors contributed equally and approved the final manuscript.
* jh.gracey@ulster.ac.uk

Check for updates

OPEN ACCESS

Citation: McCloy K, Hughes C, Dunwoody L, Marley J, Cleland I, Cruciani F, et al. (2023) The effectiveness of mindfulness alone compared to exercise and mindfulness on fatigue in women with gynaecology cancer (GEMS): Protocol for a randomised feasibility trial. PLoS One 18(10): e0278252. https://doi.org/10.1371/journal.pone.0278252

Editor: Xian-liang Liu, Charles Darwin University, AUSTRALIA

Received: December 17, 2022

Accepted: April 7, 2023

Published: October 26, 2023

Copyright: © 2023 McCloy et al. This is an open access article distributed under the terms of the Creative Commons Attribution License, which permits unrestricted use, distribution, and reproduction in any medium, provided the original author and source are credited.

Data availability statement: No datasets were generated or analyses during the current study. All relevant data from this study will be made available upon study completion.

Abstract

Background

In 2020 Globocan reported nearly 1.4 million new cases of gynaecology cancer worldwide. Cancer related fatigue has been identified as a symptom that can be present for gynaecology cancer patients many years after treatment. The current evidence around the management of this symptom suggests that exercise has the most positive outcome. However, some ambiguity remains around the evidence and whether it can address all areas of fatigue effectively. More recently, other interventions such as mindfulness have begun to show a favourable response to the management of symptoms for cancer patients. To date there has been little research that explores the feasibility of using both these interventions together in a gynaecology cancer population. This study aims to explore the feasibility of delivering an intervention that involves mindfulness and mindfulness and exercise and will explore the effect of this on fatigue, sleep, mood and quality of life.

Methods and design

This randomised control trial will assess the interventions outcomes using a pre and post design and will also include a qualitative process evaluation. Participants will be randomised into one of 2 groups. One group will undertake mindfulness only and the other group will complete exercise and mindfulness. Both groups will use a mobile application to complete these interventions over 8 weeks. The mobile app will be tailored to reflect the group the participants have drawn during randomisation. Self-reported questionnaire data will be assessed at baseline prior to commencing intervention and at post intervention. Feasibility will be assessed through recruitment,

Funding: This study is being undertaken as part of a PhD studentship (KMCC) at Ulster Univeristy and funded by the Department for the Economy (DfE) studentship. The funders had and will not have a role in study design, data collection and analysis, decision to publish, or preparation of the manuscript.

Competing interests: The authors have declared that no competing interests exist.

adherence, retention and attrition. Acceptability and participant perspective of participation (process evaluation), will be explored using focus groups.

Discussion

This trial will hope to evidence and demonstrate that combination of two interventions such as mindfulness and exercise will further improve outcomes of fatigue and well-being in gynaecology cancer. The results of this study will be used to assess (i) the feasibility to deliver this type of intervention to this population of cancer patients using a digital platform; (ii) assist this group of women diagnosed with cancer to manage fatigue and other symptoms of sleep, mood and impact their quality of life.

Trial registration

ClinicalTrials.gov (NCT05561413)

Introduction

Gynaecology cancer encompasses 5 main types, ovarian, endometrial, cervical, vaginal, and vulva [1,2]. In the United Kingdom (UK) 21,493 new cases of gynaecological cancer are diagnosed every year [2]. Treatment modalities such as surgery, chemotherapy or radiotherapy can be effective in managing and treating these cancers but may lead to unwanted long term side effects. One side effect is fatigue, the prevalence of which, for all types of cancer, was identified as 52%. For the gynaecology cancer population, this varied between 17–33%, with many women experiencing cancer related fatigue (CRF) years after treatment [3,4]. CRF has been described not only as a physical sensation, but also has emotional and cognitive symptoms suggesting that it is multi-dimensional [5]. To date, few studies have evaluated interventions that target all dimensions of fatigue, suggesting a multi-modal approach that addresses all aspects is required.

Mechanisms

Risk factors for CRF, include depression and insomnia suggesting that the presence of these are not only correlated but may also have an impact on the levels and severity of CRF [6]. Indeed, the term 'cluster symptom' has recently been adopted to describe this and the most common symptoms in this cluster have been identified as depression, insomnia, and fatigue [7]. This suggests that managing fatigue may require the incorporation of interventions that deal with the multiple symptoms of depression, insomnia, and fatigue collectively [8,9].

Existing knowledge

Research indicates that exercise can have a positive effects on CRF [10–15]. However, the effect of exercise on CRF and quality of life (QoL) remains unclear [1,16] with some studies having reported positive outcomes and others demonstrating no

change [15,17,18]. This ambiguity may suggest that exercise alone may not be enough to ameliorate CRF and improve QoL and a multidimensional approach may be required.

Despite the evidence of the positive effect of exercise on CRF, adherence to exercise remains a problem, with less than 20% of women with ovarian cancer exercising regularly following treatment [19]. Barriers to exercise include time constraints, cost, weather, side effects of treatment or exercise such as fatigue, social aspects, not being aware of physical activity guidelines, fatigue, and psychological barriers, such as motivation [20,21]. In contrast, the facilitators to exercise identified within the literature were improved physical and mental wellbeing, decreased feelings of stress, enjoyment, satisfaction, improved levels of CRF and control over health [22–24].

Research indicates that mindfulness can have a positive effects on both CRF and QoL [25–29]. Mindfulness involves being intentionally aware of the present moment and doing this without judgement. Through practice participants develop an awareness of current emotions and thoughts with compassion and kindness which in turn will lead to better control of cognition, emotion and behaviour [30]. However, some of the effect sizes for mindfulness on psychological and physical outcomes such as depression, anxiety, fatigue were small, and did not reach minimal clinical significance. Despite this, it seems that mindfulness had positive effects on psychological distress, which is defined as an emotion that is not pleasant [31–33]. This is of importance as psychological distress and particularly symptoms of anxiety, depression, sleep, stress and quality of life have been shown to improve following the introduction of mindfulness [34–36]. Some of these symptoms are also present within the 'cluster symptoms' previously identified suggesting that there may also be an improvement in CRF alongside these symptoms.

There is evidence that combining exercise and other non-pharmacological interventions such as cognitive behavioural therapy or relaxation therapy have a significant positive effect on CRF [37]. Indeed it could be argued that yoga or Tai Chi could be classified as a combination of exercise and mindfulness as it is a form of low-intensity exercise and uses breathing and inner awareness some of the elements of mindfulness [38]. However, awareness is only one aspect of mindfulness and to be truly mindful other aspects of intention and non-judgement need to be present. Also, the variability in the styles and length of yoga interventions and the lack of yoga as a specific intervention for CRF makes it difficult to assess its effectiveness for CRF [39]. Whereas mindfulness and exercise within this study follow a more structured format allowing for gradual increase and evaluation for participants.

Digital interventions for health have grown in number with over 50,000 medical or healthcare available on Google Play or the Apple Apps Store [40]. For cancer patients apps can have various aims including to provide education, identify and mange symptoms, lifestyle promotion and functional exercise [41]. A recent review identified how digital and interactive health interventions had a positive effect on physical and psychological symptoms on women with breast cancer. This review identified interventions such as music therapy, physical training such as walking, swimming, resistance training, dance, support and education and how they were delivered through a digital platform successfully and showed a favourable response [42]. Mindfulness when delivered digitally has also been shown to be feasible and result in a positive outcome [25,43,44]. Suggesting that the delivery of various interventions for cancer patients can be achieved remotely increasing the scale and inclusivity of a wider population.

Need for a trial

The majority of studies that involve exercise or mindfulness for the management of CRF and psychological distress are in the breast cancer population making it difficult to generalise findings to other cancer populations [45,46]. Both exercise and mindfulness have individually as interventions demonstrated positive effects on CRF, anxiety, depression, and sleep. Whether mindfulness and particularly adding mindfulness to an exercise regime for managing fatigue in women with gynaecology cancer is of benefit, is yet to be explored. Therefore, the aim of this study is to assess how feasible and acceptable the digital delivery of interventions of, mindfulness, and exercise, will be and if there is any effect on CRF for those with gynaecology cancer.

Theoretical model

It has been suggested that for the successful development and implementation of complex interventions a theoretical model should be used [47]. For the interventions within this study the COM-B model [48] was used as to support engagement in exercise and/or mindfulness. This model denotes that for behaviour to occur, three conditions must be met, the individual must have the Capability (both physical and psychological), the Opportunity (social and environmental) and the Motivation (reflective and automatic) to engage in the desired behaviour(s). The COM-B model is a hub that sits within the Behaviour Change Wheel (BCW), which was synthesised from previous frameworks and enhances the development of behavioural interventions [49,50]. The second layer of the BCW includes nine intervention functions (education, persuasion, incentivisation, coercion, training, enablement, modelling, environment restructuring and restrictions), which are linked to the 93 behaviour change techniques (BCTs), as described in the Behaviour Change Techniques version 2 [51]. BCTs are the smallest active ingredients of an intervention, documenting them in a standardised way can aid intervention description and replication [52]. The delivery of intervention functions is supported by the outer layer of the BCW, which includes policy categories of environment/social planning; communication/marketing, fiscal measures, legislation, regulation, service provision and guidelines. The interventions within this study used a range of intervention functions in combination which may then impact the constructs of capability, opportunity and motivation and were linked to appropriate BCTs (Box 1).

Box 1. Description of intervention functions* and behaviour change techniques† used in the interventions

Reflective

- *Education:* through app the information on the health consequences of exercise how may improve symptoms of fatigue, sleep, mood. The pros and cons of the activities (BCT group 5 and 9)

Mindfulness Intervention

Capability

- *Education:* audio and written instructions through app will guide participation to perform the activities (BCT group 6) and within the mobile app written and audio information on the health benefits and consequences (BCT group 5).
- *Training:* formal practices within the app along with weekly contact to enable an action plan to perform the practice (BCT group 1), *enabling* the regular practice which will then lead to habit forming (BCT group 8).

Opportunity

Social opportunity

- *Environmental restructuring:* family support to enable participants to complete activities along with finding an environment that is *quite* and suitable for practice (BCT group3).

Motivation

Automatic

- *Incentivisation*: given through feedback as praise and encouragement of activities and completion of goals (group 2).

Reflective

- *Education:* through app the information on the health consequences of exercise how may improve symptoms of fatigue, sleep, mood. The pros and cons of the activities (BCT group 5 and 9)

*Nine intervention functions identified in the Behaviour Change Wheel framework: education, persuasion, incentivisation, coercion, training, restriction, environmental restructuring, modelling, enablement [48].

†Behaviour change techniques as grouped in BCT Taxonomy (v1): 93 hierarchically-clustered techniques [53,54].

Methods and design

Primary aim

To explore the feasibility of conducting an 8-week mindfulness and home-based walking and strength training compared to a mindfulness only programme for the management of CRF in women with gynaecology cancer delivered through an online application. The following objectives will assess feasibility outcomes:

1. To assess participant intervention adherence, study retention and attrition rate.
2. Explore the acceptability of using a mobile application for the delivery and data collection of the exercise and mindfulness programme.
3. Explore the perspectives of participants concerning satisfaction (e.g., intervention and delivery) perceived value, barriers and facilitators of the digital programme.

Hypotheses of efficacy. Compared to active comparator of mindfulness, the combined intervention of mindfulness and exercise will be superior in reducing fatigue and improving mood, sleep and quality of life.

Secondary aim

To evaluate the effect of an 8 week exercise and mindfulness programme on cancer related fatigue as a primary clinical outcome and the effect on anxiety, depression, sleep and QoL as a secondary clinical outcome in an digital setting.

Study design

The Gynaecology Exercise Mindfulness Study (GEMS) will be a randomised controlled open label study. This trial is registered with clinicaltrials.gov NCT05561413, the delay in registration was due to Covid 19 and ongoing iterations with the mobile app development. The authors confirm that all ongoing and related trials for this intervention are registered. Blinding to this type of study and intervention is not possible, as outcomes are self-reported and in order to complete the programme participants will need to be aware of the arm drawn. Randomisation will be performed as block randomisation using 1:1 ratio. Participants will be randomised into the intervention group, mindfulness and exercise, or the active comparator group mindfulness only. This will allow assessment of effectiveness of the intervention for both groups on the outcome measures.

Following the intervention participants will be invited to join an online focus groups which will form the qualitative evaluation.

Study setting

The GEMS trial will be conducted remotely through a mobile application interface allowing recruitment across the UK and asynchronous delivery of the intervention. This app is a protype that has been developed in collaboration with the School of Computing at Ulster University. Pilot testing of this app has taken place with the 5 user testers and adaptations to the app have been made based on this.

Measurements. Primary measures include the feasibility and acceptability of the study and will be assessed in line with the MRC guidance on developing and evaluating complex interventions [47]. Participant-reported outcome measures

(PROMs) will be collected for secondary outcomes through a battery of 6 validated questionnaires described below. Estimated completion time of questionnaires is 15–20 minutes. See Fig 1 for an overview of schedule of enrolment, interventions and assessments. At baseline, participants will also complete demographic information such as age, cancer type, education, income, and education

Primary outcome measures. Adherence:- -interventions will run over 8 weeks. The completion of 4 weeks for mindfulness and 30 minutes of home-based exercise weekly for two-thirds (67%) of the duration of the trial will be regarded as adherence to the program. These levels of adherence are based on previous studies that have used the same levels [30,55]. This will be assessed through the electronic logs that participants will be encouraged to complete along with the app data information- on frequency and duration of participant engagement with each session.

Attrition:- Following a review of web-based mindfulness intervention studies, it is estimated that the attrition rate will be approximately 20% with 10% to 15% of missing data [56,57].

Time to recruit, recruitment and retention rates will be gathered through administrative data during (pre)screening, enrolment, allocation and follow-up. Reasons for non-participation, intervention/study dropouts will be collected when possible. Acceptability of data collection will be retrieved through missing data within questionnaires.

Acceptability of the programme will be assessed using focus groups this will include participants experience on the virtual delivery and data collection along with their experience and reflections on the exercise and mindfulness components of the intervention.

Secondary outcome measures. Fatigue:- The Functional Assessment of Chronic Therapy- Fatigue (FACIT-F) subscale is a 13- item questionnaire that uses a 5 point Likert scale and has been validated for use with various types of cancer and treatments [57,58]. A lower score indicates a greater degree of fatigue, with scores less than 20 representing severe fatigue and scores greater than 45 as normal [14]

Quality of life- The Functional Assessment of Cancer Therapy General (FACT-G) is a 28- item scale. It assesses physical, social, emotional and functional wellbeing. [59]. Higher scores indicate better quality of life [30]

Psychological outcomes will be assessed using the Hospital Anxiety and Depression Scale (HADS) [60]. The HADS consists of 14 questions, 7 each for assessing anxiety and depression [61]. A HADS score of greater than 11 is more defining of anxiety and depression, with a reduction of 3 points indicating a clinical improvement [62].

Sleep- The Pittsburgh Sleep Quality Index (PSQI) is a 19 items questionnaire that measures 7 domains of sleep, scored on a 0–3 scale over 1 month [63]. PSQI scores can range from 0–21, higher scores indicate poorer sleep quality [64].

Mindfulness- The 24 item Five Facet Mindfulness Questionnaire Short Form (FFMQ-SF) is a 5 point Likert Scale which assess mindfulness in daily life. It measures 5 sub-scales: observing, describing, acting with awareness, non-judgement of inner experience and non-reacting to inner experience. Higher scores indicate more mindful in everyday life [65,66]

Participants exercise behaviour will be assessed using the International physical activity questionnaire short form (IPAR-Q -SF). This questionnaire has 7 questions which assess the intensity, frequency and duration of exercise that participants engaged in over the last week.

Data collection and procedure

Data collection and procedure within the study are presented in the flow chart below (Fig 2).

Recruitment. Recruitment to the study will be facilitated through cancer charities who will reach out to their service users via social media, forums and groups. The advert will have e-mail contact details for the research team where potential participants can self-refer for further information. When potential participants contact the research team for information the participant information sheet can be e-mailed to them. Those who wish to take part in the study will be screened for eligibility, which will assess levels of fatigue, current levels of physical activity, and co-morbidities including

Study Timeline

	STUDY PERIOD							
	Enrolment	Allocation	Post-allocation					Close-out
TIMEPOINT**	$-t_1$	0	t_1				t_3	t_x
**ENROLMENT:**								
**Eligibility screen**	X							
**Informed consent**	X							
**Allocation**		X						
**INTERVENTIONS:**								
Mindfulness only								
Mindfulness and Exercise.								
**ASSESSMENTS:**								
Demographics	X	X						
FACIT-F*		X						X
FACT-G**		X						X
PSQI***		X						X
FFM-Q†		X						X
HADS†*		X						X
IPAQ-SF††		X						X

* The Functional Assessment of Chronic Therapy- Fatigue (FACIT-F)

** The Functional Assessment of Cancer Therapy General (FACT-G)

*** Pittsburgh Sleep Quality Index (PSQI)

†Five Facet Mindfulness Questionnaire Short Form (FFMQ-SF)

†*Hospital Anxiety and Depression Score (HADS)

††International physical activity questionnaire short form (IPAR-Q -S

Fig 1. Schedule of enrolment, interventions, and assessment.

https://doi.org/10.1371/journal.pone.0278252.g001

physical and psychological health. Those who meet the criteria will receive an e-mail link to complete the consent form through Qualtrics© online software. Once consented participants will receive a further Qualtrics© link, containing the online questionnaire (PROMs) that must be filled out (once before randomisation at baseline and then at 8 weeks post intervention).

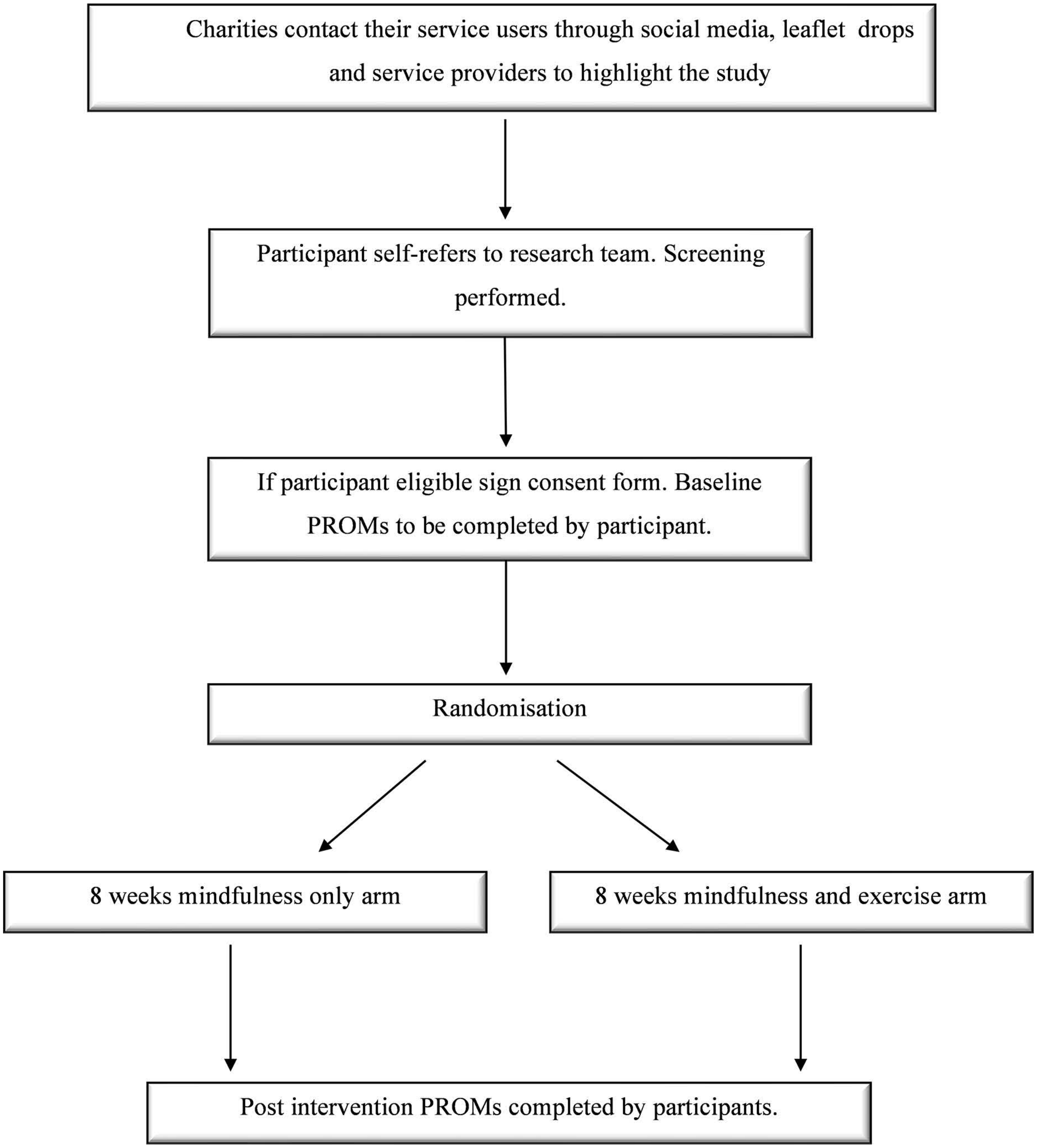

Fig 2. Flow chart of study logistics and data collection.

https://doi.org/10.1371/journal.pone.0278252.g002

Allocation. Participants will be randomly allocated to either group through 1:1 allocation. Block randomisation will be used with fixed block sizes of 10 to ensure an even distribution of groups will be achieved. Study randomisation will be done through Study Randomizer (2017) a web based randomisation service. One member of the research team (KMCC) will carry out all randomisation. Once enrolled all baseline questionnaires (PROMs) have been completed Study Randomizer will be used for allocation to groups [67]. Participants will be notified of group via e-mail.

Drop out and data retention. If a participant no longer wishes to take part in the study, they can inform the research team of their decision either through e-mail or phone. The study team will contact the participant who has withdrawn either by e-mail or phone to ask if they are willing to provide a reason for withdrawal. In line with institutional requirements, data will be retained for 10 years.

Inclusion Criteria. Participants must fulfil the following criteria to be eligible for inclusion in the study:

- Women diagnosed with gynaecology cancer Over the age of 18
- Still be experiencing fatigue at level 4 or above when assessed on a 10 point single-item scale where 0 = 'no fatigue' and 10 = 'greatest possible fatigue' [68]
- Currently sedentary (exercising < once a week for 20 minutes at a vigorous intensity or, two times per week for 30 minutes at moderate intensity or < 20 minutes three times per week, for the past 6 months).
- A minimum of 3 months post-treatment and up to 5 years.

Exclusion Criteria.

- Currently actively and regularly practising mindfulness
- Have a confirmed diagnoses of schizophrenia spectrum disorder, bipolar disorder, post-traumatic stress disorder, or risk factors for psychosis (e.g., personality disorder)
- Have an existing medical condition that may inhibit safe participation in the exercise part of the intervention study.

Interventions

Exercise intervention. The exercise component of the study is based around home-based walking and strength and conditioning exercise which have been shown to not only be effective for cancer-related fatigue but also feasible and acceptable [69,70]. Indeed, the research team have demonstrated the positive effects with women who have gynaecology cancer in a previous study and this work will further progress these findings [15,22]. The overall aim of the programme is to achieve a goal of 30 minutes of walking 3 times per week and 2–3 strength sessions, with major muscle groups per week by the end of the 8-week intervention [15,71]. These goals are suggestions, and this programme will use an inductive approach to goal setting which will be decided through collaboration with participants and individual goal setting. A goal setting diary will be provided to participants to aid them to set and monitor goals throughout the study. Alongside this participants can set goals within the mobile app. This approach has been used in previous research conducted by this team in 'Back on Track' and 'EXACT' trials [72,73].

All materials including audio, video and written information and demonstrations of individual strength exercises, for the study will be delivered through the mobile app which participants can download following randomisation. This programme is delivered asynchronously so participants can commence the programme as soon as they download the app and choose when they wish to complete the interventions. This also allows for rolling recruitment and no requirement for a set number of participants to be recruited and randomised before any participants can commence the study. Participants will be asked to report their activities daily through the app, a daily reminder will be sent to aid reporting. Participants will be contacted by the researcher every week at a mutually agreed time. Contact will be used to assess if there are any issues, set goals for the following week and capture any missing data a standardised questionnaire will be used to assess this. The researchers that will be making the weekly contact are qualified oncology nurses and physiotherapists with experience and knowledge of issues that may arise and therefore will assess and escalate if required. Further training in mindfulness was also undertaken to ensure awareness of potential issues (KMCC). A distress protocol which has been approved by the ethical committee is in place and will be adhered to. Engagement and usage of the app will be captured, and this data will be analysed feasibility of delivering this type of intervention through this digital medium.

Mindfulness. The mindfulness component of this study will be based on the mindfulness-based stress reduction developed by Jon Kabat-Zinn [74]. This will involve 8 weeks of homebased formal and informal mindfulness practice which will include body scan, sitting, walking, mountain and loving-kindness practice [27,28,75]. Similar to the exercise interventions all mindfulness practices and materials are within the app allowing participants to access these when it suits them. Each week new materials and mindfulness practices will be added to the app allowing participants to build the practice as the weeks progress. The estimated time to complete each weekly online session will be between 1–2 hours with daily practices varying between 10 minutes in the initial weeks to up to 25 mins for 5–6 days per week by week 8, this can be adapted to suit the individual schedule and goals which will be discussed with participants during the weekly contact call. Logging of mindfulness practice will be encouraged through the daily app reminder. All participants will be screened to ensure there are no contraindications to undertaking mindfulness. Even though it is generally well tolerated, there is a possibility of side effects such as increased anxiety, participants will be made aware of this and encouraged to report any issues to the research team [76]. A distress protocol which has been approved by the ethical committee is in place and will be endured.

Digital procedure. Participants will be sent written detailed information on how to download the mobile app. The first step involves registration via a portal which allows the secure collecting of app data. This will generate an e-mail which gives a link to the downloading of the app dependent on the device the participant uses [77]. Once app is downloaded, logged in and opened participants will view a home page which will give a running total of activity/mindfulness material accessed and walking/exercise goals achieved. The home tab also has a send report feature where participants can upload reports of the activity for the day. The other tabs at the top include activity and mindfulness which once clicked on will display the list of material for these weeks and a visual graph which enables self-monitoring. Within the activity tab participants can set their goals and also send reports via this area. The app is designed to take into account randomisation so that participants will only be able to view the content relevant to the group they drew during randomisation. The data entered by the participants will be able to be viewed by the study team via an administration portal allowing for monitoring and feedback to participants [77]. (Fig 3. screen shots of app).

Reprinted from [78] under a CC BY licence, with permission from Cold Spring Harbour Laboratory Press, original copyright 2022.

Process evaluation

Following completion of the 8-week intervention, participants will be invited to take part in focus groups to allow evaluation of acceptability and satisfaction of taking part in the study from the participants perspective. As the whole intervention will be conducted remotely, focus groups will be facilitated online via Microsoft Teams. This format for focus group has certain reported advantages, such as reduced dropouts, more relaxing for participants resulting in better communication, cost effectiveness, time saving in travel and the ability to include participants who live in more remote areas from the study centre [79]. The number and size of groups will be dependent on trial recruitment numbers and continue until data saturation, which will be reached when no new information is generated [80]. Separate groups will take place for each intervention group to ensure that each group can share and reflect on their experience of the intervention they took part in. Probe open-ended questions shaped by relevant literature and study objectives will be used to keep the discussion flowing. All focus group discussion will be audio and video recorded, then transcribed verbatim for analysis [81]

Focus groups data will be subjected to Thematic Analysis. This type of analysis is flexible in that it allows for the in-depth identification and interpretation of themes [82,83]. The phases of the analysis will include: familiarising with data, generating initial codes, searching for themes, reviewing themes, defining and naming themes and integrating these themes into a final report for publication [84].

Sample size. The sample size for this study is based upon feasibility and review of similar studies. The literature indicates that feasibility studies of similar interventions and methodologies within the cancer population recruit on average

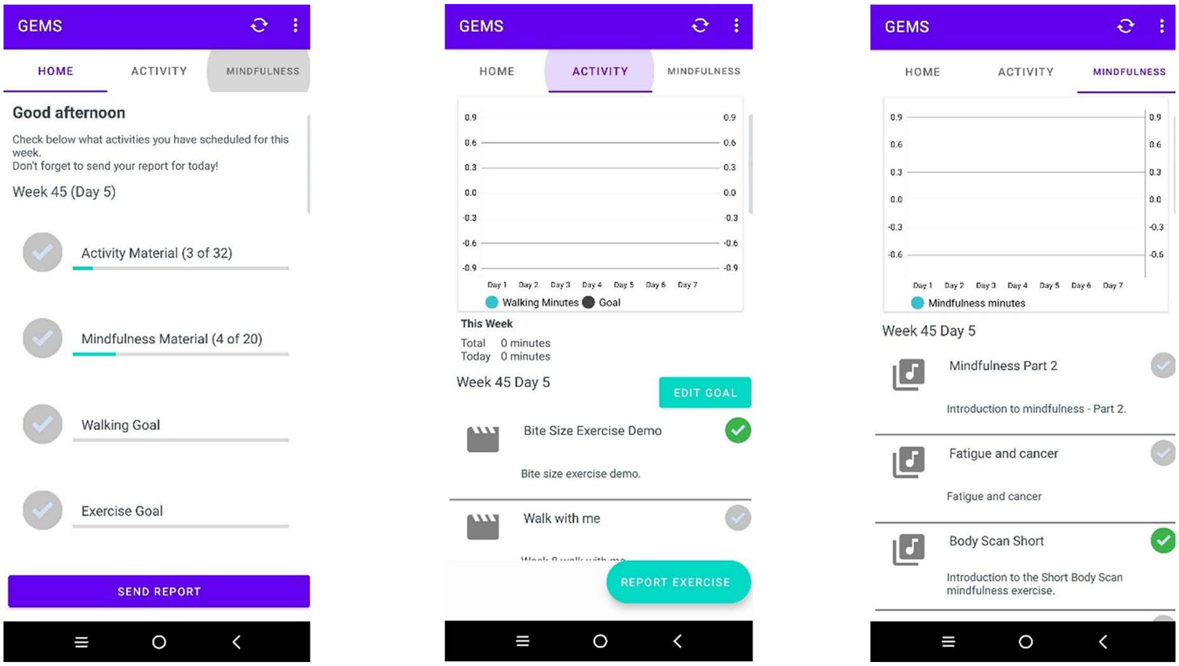

Fig 3. Mobile app screen shots.

https://doi.org/10.1371/journal.pone.0278252.g003

40 participants in total [62,85,86]. Therefore, it is anticipated that this study will recruit a total of 40 participants with 20 in each arm of the study.

Statistical analysis

As this is a feasibility study descriptive statistics will be performed to produce mean values, standard deviation and median for attrition, adherence and retention rates. Adherence will be assessed by the total number of days divided by the total number of available days expressed as a percentage together with the data that will be captured via the app which will assess the numbers and frequencies of participants' engagement with the app material (videos, audios, goal setting and diary enter). Studies have suggested that dose may have an impact on outcomes with greater adherence showing more favourable outcomes [87]. The relationship between app adherence, dose and outcomes will be explored in this study.

Attrition will be calculated by the number of participants dropping out for any reason divided by the total number randomised and expressed as a percentage. Retention will be calculated by the number and proportion of participants completing the intervention which will be number of weeks completed divided by the number of possible weeks within the intervention (8 weeks) expressed as a percentage.

Although this study is not powered to detect clinically significant changes in outcomes measures, we will collect data on the outcomes of fatigue, depression, anxiety, sleep and QoL to determine whether intervention resulted in any change that would be feasible for use in a larger evaluation. For fatigue, the Functional Assessment of Chronic Illness Therapy-fatigue (FACIT) will be used. The scoring for this outcome is a range of 0–52 with a higher score indicating less fatigue. Items 7 and 8 are reversed scored, missing data is accounted for by multiplying the total raw score by 13 and dividing by the number of items answered. For anxiety and depression, the Hospital Anxiety and Depression Scale (HADS) uses 7 questions each to assess anxiety and depression. The range of score are 0–21 with 0–7 being normal, 8–10 borderline abnormal,

11–21 abnormal. Missing data will use the half rule so if half the questionnaire is answered this will minimise missing data [88]. The Pittsburgh Sleep Quality Index will be used to measure sleep the score range is 0–21 with higher score indicating worse sleep. The scale has 7 sub-components of sleep; duration of sleep, sleep disturbance, sleep latency, dysfunction due to sleep, sleep efficiency, sleep quality and sleep medication. The 7 components are weighted equally on a scale of 0 (no difficulty) to 3 (severe difficulty) the sub-components are totalled to give an overall sleep score [63]. The Functional Assessment for Cancer Therapy-General was used to assess QoL. This questionnaire measures the quality of life through four domains; physical, social, emotional and functional well-being on 5 point Likert scale with 0 (not at all) to 4 (very much). The score range is between 0–108 with higher score indicating a better quality of life. The subscale scores are calculated first by reversing negatively stated items and then summing the scores for that subscale. Missing items are dealt with by multiplying the score by the number of questions with that scale and dividing this by the items answered. A template is provided or for each measure with a framework that guides the process for items that are reverse score required and handling of missing data [89]

Data management and protection

Participants will be allocated a unique code so that anonymity is protected. All data will be stored on a secure server at Ulster University. Databases will be encrypted, as will any transferred data and held securely in a repository. The key to the participant IDs will be held by the Chief Investigator. Data will be kept on password-protected computers that can only be accessed by the research team. Access to the anonymised data will use authentication and be traceable by login details. Participants will be made aware that confidentiality will be maintained but the research team will be unable to prevent other members that participate in online focus groups to adhere to this confidentiality agreement. Participants will also be informed that if they withdraw from this part of the study that once data has been anonymised it may be difficult to remove it.

Ethics. Ethical approval has been granted by Ulster University Research Ethics Committee (REC.21.0076).

Dissemination Policy. The findings of the research will be communicated with those who participated in the study either via email or a local event. The researcher will disseminate the projects findings and outputs via oral and poster presentations at local, national and international conferences and publications in peer-reviewed journal papers.

Discussion

Fatigue continues to be a distressing symptom for many cancer patients either pre diagnosis, diagnosis, during and post treatment. For some it continues to be a long term symptom that effects all aspects of life including social, biological, financial and emotionally leading to reduced quality of life. The current evidenced-based advice and interventions offered for managing this symptom include exercise, nutrition advice, sleep hygiene, or prioritising, pacing and planning activities [90]. In recent years other interventions such as mindfulness, have been shown to have a favourable responses for CRF, which in turn also demonstrated a positive effect on stress and sleep all of which are identified as part of the cluster symptom for fatigue [91]. However, there appears to be little research that examine the use of more than one intervention simultaneously. This study will not only explore whether this is feasible but also if the simultaneous delivery of interventions results in any additionally effect. Furthermore, the use of an active comparator will be of value, as it has been suggested previously that a usual care or wait list control can result in a greater result in the experimental arm [35]. Additionally, it has been indicated that assessing the levels of fatigue prior to entering a study can have an impact on the effectiveness of the intervention. This was demonstrated in a meta-analysis that examined the effectiveness of physical activity on fatigue for colorectal cancer patients. It found that levels of fatigue in some of the included studies within this review were equivalent to the normal population which resulted in participant outcome measures showing little improvement therefore described as a ceiling effect for the interventions [45]. Therefore, this study has been designed around these concepts and will be both assessing levels of fatigue as part of the screening process and incorporating mindfulness as the active comparator arm.

This study will also use a digital platform for delivering this intervention. By exploring the feasibility of delivering the intervention through this platform it may also make it feasible in the future to diversify this app and its delivery for other types of cancer populations and possibly management of other symptoms.

Supporting information

S1 Checklist. SPIRIT 2013 checklist: Recommended items to address in a clinical trial protocol and related documents.
(DOC)

S1 File. Study protocol.
(DOCX)

S2 File. Trial registration data
(DOCX)

Author contributions

Conceptualization: Kairen McCloy, Ciara Hughes, Lynn Dunwoody, Joanne Marley, Ian Cleland.

Federico Cruciani, Catherine Saunders, Jackie Gracey.

Funding acquisition: Ciara Hughes, Lynn Dunwoody, Joanne Marley, Jackie Gracey.

Investigation: Kairen McCloy, Jackie Gracey.

Methodology: Kairen McCloy, Ciara Hughes, Lynn Dunwoody, Joanne Marley, Ian Cleland.

Federico Cruciani, Catherine Saunders, Jackie Gracey.

Project administration: Kairen McCloy, Ciara Hughes, Lynn Dunwoody, Joanne Marley.

Jackie Gracey.

Resources: Kairen McCloy, Ciara Hughes, Lynn Dunwoody, Joanne Marley, Jackie Gracey.

Software: Ian Cleland, Federico Cruciani, Catherine Saunders.

Supervision: Ciara Hughes, Lynn Dunwoody, Joanne Marley, Jackie Gracey.

Writing – original draft: Kairen McCloy.

Writing – review & editing: Kairen McCloy, Ciara Hughes, Lynn Dunwoody, Joanne Marley.

Ian Cleland, Federico Cruciani, Catherine Saunders, Jackie Gracey.

References

1. Lin K-Y, Frawley HC, Denehy L, Feil D, Granger CL. Exercise interventions for patients with gynaecological cancer: a systematic review and meta-analysis. Physiotherapy. 2016;102(4):309–19. https://doi.org/10.1016/j.physio.2016.02.006 PMID: 27553642
2. Gynaecological Cancer Research Charity. 2020. Accessed 2021 April 22. https://eveappeal.org.uk/
3. Sekse RJT, Hufthammer KO, Vika ME. Fatigue and quality of life in women treated for various types of gynaecological cancers: a cross-sectional study. J Clin Nurs. 2015;24(3–4):546–55. https://doi.org/10.1111/jocn.12647 PMID: 25040957
4. Ma Y, He B, Jiang M, Yang Y, Wang C, Huang C, et al. Prevalence and risk factors of cancer-related fatigue: A systematic review and meta-analysis. Int J Nurs Stud. 2020;111:103707. https://doi.org/10.1016/j.ijnurstu.2020.103707 PMID: 32920423
5. de Raaf PJ, de Klerk C, van der Rijt CCD. Elucidating the behavior of physical fatigue and mental fatigue in cancer patients: a review of the literature. Psychooncology. 2013;22(9):1919–29. https://doi.org/10.1002/pon.3225 PMID: 23147803
6. Bower JE. Cancer-related fatigue--mechanisms, risk factors, and treatments. Nat Rev Clin Oncol. 2014;11(10):597–609. https://doi.org/10.1038/nrclinonc.2014.127 PMID: 25113839
7. He X, Ng MSN, Wang X, Guo P, Li L, Zhao W, et al. A Dance Program to Manage A Fatigue-Sleep Disturbance-Depression Symptom Cluster among Breast Cancer Patients Receiving Adjuvant Chemotherapy: A Feasibility Study. Asia Pac J Oncol Nurs. 2021;8(3):337–9. https://doi.org/10.4103/2347-5625.308677 PMID: 33850968

8. Naraphong W, Lane A, Schafer J, Whitmer K, Wilson BRA. Exercise intervention for fatigue-related symptoms in Thai women with breast cancer: A pilot study. Nurs Health Sci. 2015;17(1):33–41. https://doi.org/10.1111/nhs.12124 PMID: 24636322
9. Poort H, de Rooij BH, Uno H, Weng S, Ezendam NPM, van de Poll-Franse L, et al. Patterns and predictors of cancer-related fatigue in ovarian and endometrial cancers: 1-year longitudinal study. Cancer. 2020;126(15):3526–33. https://doi.org/10.1002/cncr.32927 PMID: 32436610
10. Cramp F, Byron-Daniel J. Exercise for the management of cancer-related fatigue in adults. Cochrane Database of Systematic Reviews. 2012;(11).
11. Tian L, Lu HJ, Lin L, Hu Y. Effects of aerobic exercise on cancer-related fatigue: a meta-analysis of randomized controlled trials. Support Care Cancer. 2016;24(2):969–83. https://doi.org/10.1007/s00520-015-2953-9 PMID: 26482381
12. Juvet LK, Thune I, Elvsaas IKØ, Fors EA, Lundgren S, Bertheussen G, et al. The effect of exercise on fatigue and physical functioning in breast cancer patients during and after treatment and at 6 months follow-up: A meta-analysis. Breast. 2017;33:166–77. https://doi.org/10.1016/j.breast.2017.04.003 PMID: 28415013
13. Schuler MK, Hentschel L, Kisel W, Kramer M, Lenz F, Hornemann B, et al. Impact of Different Exercise Programs on Severe Fatigue in Patients Undergoing Anticancer Treatment—A Randomized Controlled Trial. J Pain Symptom Manage 2017;53(1):57–66.
14. Sheehan P, Denieffe S, Murphy NM, Harrison M. Exercise is more effective than health education in reducing fatigue in fatigued cancer survivors. Support Care Cancer. 2020;28(10):4953–62. https://doi.org/10.1007/s00520-020-05328-w PMID: 32020356
15. Donnelly CM, Blaney JM, Lowe-Strong A, Rankin JP, Campbell A, McCrum-Gardner E. A randomised controlled trial testing the feasibility and efficacy of a physical activity behavioural change intervention in managing fatigue with gynaecological cancer survivors. Gynecol Oncol. 2011;122(3):618–24.
16. Velthuis MJ, Agasi-Idenburg SC, Aufdemkampe G, Wittink HM. The effect of physical exercise on cancer-related fatigue during cancer treatment: a meta-analysis of randomised controlled trials. Clin Oncol (R Coll Radiol). 2010;22(3):208–21. https://doi.org/10.1016/j.clon.2009.12.005 PMID: 20110159
17. Maqbali M, Hughes C, Dunwoody L, Rankin JP, Hacker ED, Gracey J. Exercise Interventions to Manage Fatigue in Women With Gynecologic Cancer: A Systematic Review. Oncol Nurs Forum. 2019;46(1):71–82. https://doi.org/10.1188/19.ONF.71-82 PMID: 30547952
18. Jiang M, Ma Y, Yun B, Wang Q, Huang C, Han L. Exercise for fatigue in breast cancer patients: An umbrella review of systematic reviews. Int J Nurs Sci. 2020;7(2):248–54. https://doi.org/10.1016/j.ijnss.2020.03.001 PMID: 32685623
19. Joly F, Ahmed-Lecheheb D, Kalbacher E, Heutte N, Clarisse B, Grellard JM, et al. Long-term fatigue and quality of life among epithelial ovarian cancer survivors: a GINECO case/control VIVROVAIRE I study. Ann Oncol. 2019;30(5):845–52. https://doi.org/10.1093/annonc/mdz074 PMID: 30851097
20. Hardcastle SJ, Maxwell-Smith C, Kamarova S, Lamb S, Millar L, Cohen PA. Factors influencing non-participation in an exercise program and attitudes towards physical activity amongst cancer survivors. Support Care Cancer. 2018;26(4):1289–95. https://doi.org/10.1007/s00520-017-3952-9 PMID: 29090387
21. Lavallée JF, Abdin S, Faulkner J, Husted M. Barriers and facilitators to participating in physical activity for adults with breast cancer receiving adjuvant treatment: A qualitative metasynthesis. Psychooncology. 2019;28(3):468–76. https://doi.org/10.1002/pon.4980 PMID: 30657225
22. Blaney JM, Lowe-Strong A, Rankin-Watt J, Campbell A, Gracey JH. Cancer survivors' exercise barriers, facilitators and preferences in the context of fatigue, quality of life and physical activity participation: a questionnaire-survey. Psychooncology. 2013;22(1):186–94. https://doi.org/10.1002/pon.2072 PMID: 23296635
23. Gho SA, Munro BJ, Jones SC, Steele JR. Perceived exercise barriers explain exercise participation in Australian women treated for breast cancer better than perceived exercise benefits. Phys Ther. 2014;94(12):1765–74.
24. Clifford BK, Mizrahi D, Sandler CX, Barry BK, Simar D, Wakefield CE, et al. Barriers and facilitators of exercise experienced by cancer survivors: a mixed methods systematic review. Support Care Cancer. 2018;26(3):685–700. https://doi.org/10.1007/s00520-017-3964-5 PMID: 29185105
25. Lengacher CA, Reich RR, Ramesar S, Alinat CB, Moscoso M, Cousin L, et al. Feasibility of the mobile mindfulness-based stress reduction for breast cancer (mMBSR(BC)) program for symptom improvement among breast cancer survivors. Psychooncology. 2018;27(2):524–31. https://doi.org/10.1002/pon.4491 PMID: 28665541
26. Johns SA, Brown LF, Beck-Coon K, Talib TL, Monahan PO, Giesler RB, et al. Randomized controlled pilot trial of mindfulness-based stress reduction compared to psychoeducational support for persistently fatigued breast and colorectal cancer survivors. Support Care Cancer. 2016;24(10):4085–96. https://doi.org/10.1007/s00520-016-3220-4 PMID: 27189614
27. Johns SA, Brown LF, Beck-Coon K, Monahan PO, Tong Y, Kroenke K. Randomized controlled pilot study of mindfulness-based stress reduction for persistently fatigued cancer survivors. Psychooncology. 2015;24(8):885–93. https://doi.org/10.1002/pon.3648 PMID: 25132206
28. Reich RR, Lengacher CA, Alinat CB, Kip KE, Paterson C, Ramesar S, et al. Mindfulness-Based Stress Reduction in Post-treatment Breast Cancer Patients: Immediate and Sustained Effects Across Multiple Symptom Clusters. J Pain Symptom Manage. 2017;53(1):85–95. https://doi.org/10.1016/j.jpainsymman.2016.08.005 PMID: 27720794
29. Carlson LE. Mindfulness meditation for younger breast cancer survivors: a randomized controlled trial. Breast diseases. 2015;26(4):294–6.
30. Park S, Sato Y, Takita Y, Tamura N, Ninomiya A, Kosugi T, et al. Mindfulness-Based Cognitive Therapy for Psychological Distress, Fear of Cancer Recurrence, Fatigue, Spiritual Well-Being, and Quality of Life in Patients With Breast Cancer-A Randomized Controlled Trial. J Pain Symptom Manage. 2020;60(2):381–9. https://doi.org/10.1016/j.jpainsymman.2020.02.017 PMID: 32105790

31. Cramer H, Lauche R, Paul A, Dobos G. Mindfulness-based stress reduction for breast cancer-a systematic review and meta-analysis. Curr Oncol. 2012;19(5):e343-52. https://doi.org/10.3747/co.19.1016 PMID: 23144582
32. Haller H, Winkler MM, Klose P, Dobos G, Kümmel S, Cramer H. Mindfulness-based interventions for women with breast cancer: an updated systematic review and meta-analysis. Acta Oncol. 2017;56(12):1665–76. https://doi.org/10.1080/0284186X.2017.1342862 PMID: 28686520
33. Cillessen L, Johannsen M, Speckens AEM, Zachariae R. Mindfulness-based interventions for psychological and physical health outcomes in cancer patients and survivors: A systematic review and meta-analysis of randomized controlled trials. Psychooncology. 2019.
34. Lin L, Lin L, Tzeng G, Huang Y, Tai J, Chen Y. Effects of mindfulness-based therapy for cancer patients: A systematic review and meta-analysis. J Clin Psychol Med Settings. 2022.
35. Johns SA, Tarver WL, Secinti E, Mosher CE, Stutz PV, Carnahan JL, et al. Effects of mindfulness-based interventions on fatigue in cancer survivors: A systematic review and meta-analysis of randomized controlled trials. Crit Rev Oncol Hematol. 2021;160:103290. https://doi.org/10.1016/j.critrevonc.2021.103290 PMID: 33675902
36. McCloy K, Hughes C, Dunwoody L, Marley J, Gracey J. Effects of mindfulness-based interventions on fatigue and psychological wellbeing in women with cancer: A systematic review and meta-analysis of randomised control trials. Psychooncology. 2022;31(11):1821–34. https://doi.org/10.1002/pon.6046 PMID: 36221152
37. Hilfiker R, Meichtry A, Eicher M, Nilsson Balfe L, Knols RH, Verra ML, et al. Exercise and other non-pharmaceutical interventions for cancer-related fatigue in patients during or after cancer treatment: a systematic review incorporating an indirect-comparisons meta-analysis. Br J Sports Med. 2018;52(10):651–8. https://doi.org/10.1136/bjsports-2016-096422 PMID: 28501804
38. Song J, Wang T, Wang Y, Li R, Niu S, Zhuo L, et al. The Effectiveness of Yoga on Cancer-Related Fatigue: A Systematic Review and Meta-Analysis. Oncol Nurs Forum. 2021;48(2):207–28. https://doi.org/10.1188/21.ONF.207-228 PMID: 33600394
39. Haussmann A, Schmidt ME, Illmann ML, Schröter M, Hielscher T, Cramer H, et al. Meta-Analysis of Randomized Controlled Trials on Yoga, Psychosocial, and Mindfulness-Based Interventions for Cancer-Related Fatigue: What Intervention Characteristics Are Related to Higher Efficacy?. Cancers (Basel). 2022;14(8):2016. https://doi.org/10.3390/cancers14082016 PMID: 35454922
40. Healthcare apps available Apple App Store 2022. Available at https://www.statista.com/statistics/779910/health-apps-available-ios-worldwide/ Accessed 2022 October 24.
41. Cai T, Huang Y, Zhang Y, Lu Z, Huang Q, Yuan C. Mobile health applications for the care of patients with breast cancer: A scoping review. Int J Nurs Sci. 2021;8(4):470–6. https://doi.org/10.1016/j.ijnss.2021.07.003 PMID: 34631997
42. Obrero-Gaitan E, Cortes-Perez I, Calet-Fernandez T, Garcia-Lopez H, Lopez Ruiz M, del Carmen, et al. Digital and interactive health interventions minimize the physical and psychological impact of breast cancer, increasing women's quality of life: A systematic review and meta-analysis. Cancers. 2022;14(17):4133.
43. Compen F, Bisseling E, Schellekens M, Donders R, Speckens A, van der Lee M, et al. Face-to-face and internet-based mindfulness-based cognitive therapy compared with treatment as usual in reducing psychological distress in patients with cancer: A multicenter randomized controlled trial. Journal of Clinical Oncology. 2018;36(23):2413–21. https://doi.org/10.1200/JCO.2018.36.23.2413
44. Bruggeman-Everts F, Wolvers MDJ, van de Schoot R, Vollenbroek-Hutten M, Van der Lee M a r i j e L. Effectiveness of two web-based interventions for chronic cancer-related fatigue compared to an active control condition: results of the "Fitter na kanker" randomized controlled trial. J Med Internet Res. 2015;19(10).
45. Brandenbarg D, Korsten JHWM, Berger MY, Berendsen AJ. The effect of physical activity on fatigue among survivors of colorectal cancer: a systematic review and meta-analysis. Supportive Care in Cancer. 2018;26(2):393–403.
46. Stelten S, Hoedjes M, Kenter GG, Kampman E, Huijsmans RJ, van Lonkhuijzen LR, et al. Rationale and study protocol of the Physical Activity and Dietary intervention in women with OVArian cancer (PADOVA) study: a randomised controlled trial to evaluate effectiveness of a tailored exercise and dietary intervention on body composition, physical function and fatigue in women with ovarian cancer undergoing chemotherapy. BMJ Open. 2020;10(11):e036854. https://doi.org/10.1136/bmjopen-2020-036854 PMID: 33148722
47. Skivington K, Matthews L, Simpson SA, Craig P, Baird J, Blazeby JM, et al. A new framework for developing and evaluating complex interventions: update of Medical Research Council guidance. BMJ. 2021;374:n2061. https://doi.org/10.1136/bmj.n2061 PMID: 34593508
48. Michie S, van Stralen MM, West R. The behaviour change wheel: a new method for characterising and designing behaviour change interventions. Implement Sci. 2011;6:42. https://doi.org/10.1186/1748-5908-6-42 PMID: 21513547
49. Cowdell F, Dyson J. How is the theoretical domains framework applied to developing health behaviour interventions? A systematic search and narrative synthesis. BMC Public Health. 2019;19(1):1180. https://doi.org/10.1186/s12889-019-7442-5 PMID: 31455327
50. Howlett N, Schulz J, Trivedi D, Troop N, Chater A. A prospective study exploring the construct and predictive validity of the COM-B model for physical activity. Journal of Health Psychology. 2019;24(10):1378–91.
51. Abraham C, Michie S. A taxonomy of behavior change techniques used in interventions. Health Psychol. 2008;27(3):379–87. https://doi.org/10.1037/0278-6133.27.3.379 PMID: 18624603
52. Howlett N, Trivedi D, Troop NA, Chater AM. What are the most effective behaviour change techniques to promote physical activity and/or reduce sedentary behaviour in inactive adults? A systematic review protocol. BMJ Open. 2015;5(8):e008573. https://doi.org/10.1136/bmjopen-2015-008573 PMID: 26246078

PLOS One

53. Yuan Y, Lin L, Xie C, Lu Y, Liang J, Qi Y, et al. Effectiveness comparisons of various psychosocial therapies for cancer-related fatigue: A Bayesian network meta-analysis. J Affect Disord. 2022.

54. BCT Taxonomy (v1): 93 hierarchically-clustered techniques. Available at file:///C:/Users/B00766410/Downloads/BCTTv1_PDF_version.pdf. Accessed 2022 October 2.

55. Kim JY, Lee MK, Lee DH, Kang DW, Min JH, Lee JW, et al. Effects of a 12-week home-based exercise program on quality of life, psychological health, and the level of physical activity in colorectal cancer survivors: a randomized controlled trial. Support Care Cancer. 2019;27(8):2933–40. https://doi.org/10.1007/s00520-018-4588-0 PMID: 30564936

56. Subnis UB, Farb NA, Piedalue K-AL, Speca M, Lupichuk S, Tang PA, et al. A Smartphone App-Based Mindfulness Intervention for Cancer Survivors: Protocol for a Randomized Controlled Trial. JMIR Res Protoc. 2020;9(5):e15178. https://doi.org/10.2196/15178 PMID: 32390591

57. Carlson LE, Subnis UB, Piedalue K-AL, Vallerand J, Speca M, Lupichuk S, et al. The ONE-MIND Study: Rationale and protocol for assessing the effects of ONlinE MINDfulness-based cancer recovery for the prevention of fatigue and other common side effects during chemotherapy. Eur J Cancer Care (Engl). 2019;28(4):e13074. https://doi.org/10.1111/ecc.13074 PMID: 31056784

58. Al Maqbali M, Hughes C, Gracey J, Rankin J, Dunwoody L, Hacker E. Quality assessment criteria: psychometric properties of measurement tools for cancer related fatigue. Acta Oncol. 2019;58(9):1286–97. https://doi.org/10.1080/0284186X.2019.1622773 PMID: 31204538

59. Cella DF, Tulsky DS, Gray G, Sarafian B, Linn E, Bonomi A, et al. The Functional Assessment of Cancer Therapy scale: development and validation of the general measure. J Clin Oncol. 1993;11(3):570–9. https://doi.org/10.1200/JCO.1993.11.3.570 PMID: 8445433

60. Zigmond AS, Snaith RP. The hospital anxiety and depression scale. Acta Psychiatr Scand. 1983;67(6):361–70. https://doi.org/10.1111/j.1600-0447.1983.tb09716.x PMID: 6880820

61. Spahn G, Choi K-E, Kennemann C, Lüdtke R, Franken U, Langhorst J, et al. Can a multimodal mind-body program enhance the treatment effects of physical activity in breast cancer survivors with chronic tumor-associated fatigue? A randomized controlled trial. Integr Cancer Ther. 2013;12(4):291–300. https://doi.org/10.1177/1534735413492727 PMID: 23766391

62. Eyles C, Leydon GM, Hoffman CJ, Copson ER, Prescott P, Chorozoglou M, et al. Mindfulness for the self-management of fatigue, anxiety, and depression in women with metastatic breast cancer: a mixed methods feasibility study. Integr Cancer Ther. 2015;14(1):42–56. https://doi.org/10.1177/1534735414546567 PMID: 25161198

63. Buysse DJ, Reynolds CF 3rd, Monk TH, Berman SR, Kupfer DJ. The Pittsburgh Sleep Quality Index: a new instrument for psychiatric practice and research. Psychiatry Res. 1989;28(2):193–213. https://doi.org/10.1016/0165-1781(89)90047-4 PMID: 2748771

64. Yu C-H, Wang T-J, Chang C-L, Liang S-Y, Wu S-F, Liu C-Y, et al. Healthy life styles, sleep and fatigue in endometrial cancer survivors: A cross-sectional study. J Clin Nurs. 2020;29(7–8):1372–80. https://doi.org/10.1111/jocn.15189 PMID: 31970842

65. Johns VonAh D, Brown LF, Beck-Coon K, Talib TL, Alyea JM, et al. Randomized controlled pilot trial of mindfulness-based stress reduction for breast and colorectal cancer survivors: effects on cancer-related cognitive impairment. Journal of cancer survivorship 2016;10(3):437-448.

66. Compen FR, Bisseling EM, Speckens AEM, Van der Lee ML, Adang EMM, Donders ART. Study protocol of a multicenter randomized controlled trial comparing the effectiveness of group and individual internet-based Mindfulness-Based Cognitive Therapy with treatment as usual in reducing psychological distress in cancer patients: The BeMind study. BMC Psychology. 2015;3(1).

67. Study Randomizer. 2017. Accessed 2022 February 8. Available at https://www.studyrandomizer.com

68. Schwartz AL, Meek PM, Nail LM, Fargo J, Lundquist M, Donofrio M, et al. Measurement of fatigue. determining minimally important clinical differences. J Clin Epidemiol. 2002;55(3):239–44. https://doi.org/10.1016/s0895-4356(01)00469-3 PMID: 11864794

69. Payne JK, Held J, Thorpe J, Shaw H. Effect of exercise on biomarkers, fatigue, sleep disturbances, and depressive symptoms in older women with breast cancer receiving hormonal therapy. Oncol Nurs Forum. 2008;35(4):635–42.

70. Yeo TP, Burrell SA, Sauter PK, Kennedy EP, Lavu H, Leiby BE, et al. A progressive postresection walking program significantly improves fatigue and health-related quality of life in pancreas and periampullary cancer patients. J Am Coll Surg. 2012;214(4):463–75; discussion 475-7. https://doi.org/10.1016/j.jamcollsurg.2011.12.017 PMID: 22321518

71. Gokal K, Wallis D, Ahmed S, Boiangiu I, Kancherla K, Munir F. Effects of a self-managed home-based walking intervention on psychosocial health outcomes for breast cancer patients receiving chemotherapy: a randomised controlled trial. Support Care Cancer. 2016;24(3):1139–66. https://doi.org/10.1007/s00520-015-2884-5 PMID: 26275768

72. Gracey JH, Watson M, Payne C, Rankin J, Dunwoody L. Translation research: “Back on Track”, a multiprofessional rehabilitation service for cancer-related fatigue. BMJ Support Palliat Care. 2016;6(1):94–6. https://doi.org/10.1136/bmjspcare-2014-000692 PMID: 25526904

73. Gracey JH, McDermott L, Murphy M, Rankin J, McNeilly AM. The feasibility and acceptability of a home based exercise intervention for colorectal cancer survivors: ‘EXACT’ – EXercise And Colorectal Cancer Trial. Physical Therapy and Rehabilitation. 2021;8(1):3.

74. Kabat-Zinn J, TNH. Full Catastrophe Living: Using the Wisdom of Your Body and Mind to Face Stress, Pain and Illness. Revised Edition ed. New York: Bantam. 2013.

75. Lengacher CA, Reich RR, Post-White J, Moscoso M, Shelton MM, Barta M, et al. Mindfulness based stress reduction in post-treatment breast cancer patients: an examination of symptoms and symptom clusters. J Behav Med. 2012;35(1):86–94. https://doi.org/10.1007/s10865-011-9346-4 PMID: 21506018

76. Van Dam NT, van Vugt MK, Vago DR, Schmalzl L, Saron CD, Olendzki A, et al. Mind the Hype: A Critical Evaluation and Prescriptive Agenda for Research on Mindfulness and Meditation. Perspect Psychol Sci. 2018;13(1):36–61. https://doi.org/10.1177/1745691617709589 PMID: 29016274

77. A reusable software platform for delivery of digital interventions. In: 14th International Conference on Ubiquitous Computing and Ambient Intelligence, 2022.
78. Mccloy K, Hughes C, Dunwoody L, Marley J, Cleland I, Cruciani F, et al. Evaluating the effectiveness of mindfulness alone compared to exercise and mindfulness on fatigue in women with gynaecology cancer (GEMS): Protocol for a randomised feasibility trial. medRxiv. 2022. https://doi.org/2022.11.14.22282320
79. Halliday M, Mill D, Johnson J, Lee K. Let's talk virtual! Online focus group facilitation for the modern researcher. Research in Social and Administrative Pharmacy. 2021.
80. Goei LPS, Lopez V, Klainin-Yobas P. Exploring the perceptions of cancer survivors of a mindfulness intervention at a tertiary hospital in Singapore: a descriptive qualitative study. Supportive Care in Cancer. 2020.
81. Barbour RS. Doing focus groups. London: SAGE Publications. 2008.
82. Holloway I, Galvin K. Qualitative Research in Nursing and Healthcare. Hoboken: John Wiley & Sons, Incorporated. 2016.
83. Castleberry A, Nolen A. Thematic analysis of qualitative research data: Is it as easy as it sounds?. Curr Pharm Teach Learn. 2018;10(6):807–15. https://doi.org/10.1016/j.cptl.2018.03.019 PMID: 30025784
84. Braun C. Using thematic analysis in psychology. Qualitative Research in Psychology. 2006;3(2):77–101.
85. Victorson D, Hankin V, Burns J, Weiland R, Maletich C, Sufrin N, et al. Feasibility, acceptability and preliminary psychological benefits of mindfulness meditation training in a sample of men diagnosed with prostate cancer on active surveillance: results from a randomized controlled pilot trial. Psychooncology. 2017;26(8):1155–63. https://doi.org/10.1002/pon.4135 PMID: 27145355
86. Price-Blackshear M, Pratscher SD, Oyler DL, Bettencourt A, Armer JM, Udmuangpia T. Online couples mindfulness-based intervention for young breast cancer survivors and their partners: A randomized-control trial. J Psychosoc Oncol. 2020;38(5):592–611.
87. Cillessen L, van de Ven MO, Compen FR, Bisseling EM, van der Lee ML, Speckens AE. Predictors and Effects of Usage of an Online Mindfulness Intervention for Distressed Cancer Patients: Usability Study. J Med Internet Res. 2020;22(10):e17526. https://doi.org/10.2196/17526 PMID: 33006567
88. Bell ML, Fiero MH, Fairclough DL, Butow PN. Handling missing items in the Hospital Anxiety and Depression Scale (HADS): A simulation study. BMC Research Notes. 2016;9(1).
89. Scoring. Accessed 2023 April 20. Available at https://www.facit.org/scoring
90. Cillessen L, van de Ven MO, Compen FR, Bisseling EM, van der Lee ML, Speckens AEM. Predictors and effects of usage of an online mindfulness intervention for distressed cancer patients: usability study. Journal of Medical Internet Research. 2020;22(10).
91. Tiredness (fatigue). Macmillan Cancer Support. Available at https://www.macmillan.org.uk/cancer-information-and-support/impacts-of-cancer/tiredness. 2018. Accessed 2022 June 8.
